# Expanding Coefficient: A Parameter To Assess the Stability of Induced-Fit Complexes

**DOI:** 10.1021/acs.orglett.1c00165

**Published:** 2021-02-16

**Authors:** Carmen Talotta, Gerardo Concilio, Margherita De Rosa, Annunziata Soriente, Carmine Gaeta, Antonio Rescifina, Pablo Ballester, Placido Neri

**Affiliations:** † Dipartimento di Chimica e Biologia “A. Zambelli”, Università di Salerno, via Giovanni Paolo II, 132, I-84084, Fisciano (Salerno), Italy; ‡ Dipartimento di Scienze del Farmaco e della Salute, Università di Catania, viale Andrea Doria, 6, I-95125 Catania, Italy; § Institute of Chemical Research of Catalonia (ICIQ), The Barcelona Institute of Science and Technology (BIST), Av. Països Catalans, 16, 43007 Tarragona, Spain

## Abstract

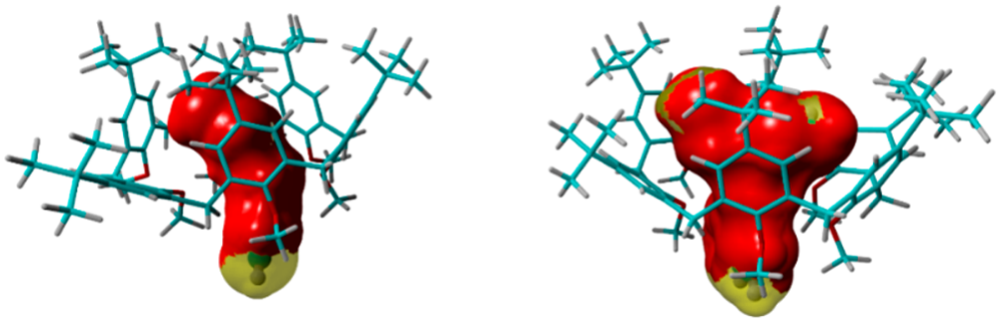

Here we propose a new parameter, the Expanding Coefficient (EC), that can be correlated with the thermodynamic stability of supramolecular complexes governed by weak secondary interactions and obeying the induced-fit model. The EC values show a good linear relationship with the log *K*_app_ of the respective pseudorotaxane complexes investigated. According to Cram’s Principle of Preorganization, the EC can be considered an approximate mechanical measure of the host’s reorganization energy cost upon adopting the final bound geometry.

Molecular recognition is a fundamental phenomenon at the ground of every biological function.^1^ Its fundamental relevance was recognized since the early days of chemical and biological sciences,^2^ and afterward, many studies have been devoted to comprehending its underlying principles and explaining the origin of the amazing selectivity^3^ or affinity^4^ observed in some instances. Two main models are currently adopted to describe a molecular recognition complexation: (i) the lock-and-key model^5^ and (ii) the induced-fit complexation.^6^ In the first one, the degree of geometrical (steric) fitting between a rigid, undeformable receptor and substrate is evaluated, while, in the second one, the best match of flexible counterparts is considered after their mutual adaptation.

In the field of synthetic receptors, a single value parameter, the Packing Coefficient (PC), defined as the ratio between the van der Waals volume of the hosted guest and the volume of the host cavity,^7^ was introduced by Rebek and Mecozzi to simplify the assessment of the complex stability for rigid, lock-and-key-like systems. It was found that the optimal PC value, the fraction of occupied volume, is 0.55 ± 0.09 (55%)^7^ in the liquid state when weak intermolecular interactions (dispersion forces, van der Waals interactions) are present. The PC can increase (up to 70–84%) when stronger interactions (hydrogen bonds, solvation effects) come into play^8a,8b^ or can decrease (down to 40%) when gaseous molecules are involved.^8c,8d^ This simple rule has been validated in many synthetic host–guest systems,^9^ has been used to explain reactivity results,^10^ and has also been extended to biological receptors.^11^

From the above definitions, it is evident that the PC parameter cannot be applied to induced-fit systems because the void receptor cavity volume tends to adapt to that of the guest to give the best fitting with it.^12^ Therefore, it is expected that the PC value of induced-fit complexes calculated for the final adapted geometry will always overcome the 55% rule (vide infra), independently from their actual stability, thus vanishing any comparison.

It is evident that another useful and straightforward rule is necessary to assess the stability of induced-fit complexes in order to predict the ideal host–guest couple. We propose here a new single-value parameter to address such point.

The induced-fit system chosen for this work is the pseudorotaxane complex formed by hexamethoxy-*p*-*tert*-butylcalix[6]arene^13^**1** and alkylbenzylammonium axles **2a**–**k**^**+**^ (Scheme 1). In previous studies,^14^ we have demonstrated that this kind of dialkylammonium axles can thread the calixarene cavity when coupled to the weakly interacting **BARF**^**–**^ (or **TFPB**^–^) “superweak anion” to give the **2^+^**⊂**1** pseudorotaxanes (Scheme 1). In addition, we have found that the aromatic cavity mostly prefers to host the alkyl portion of the axle with respect to the benzyl one (the so-called “*endo*-alkyl rule”).^14a−14e^

**Scheme 1:**
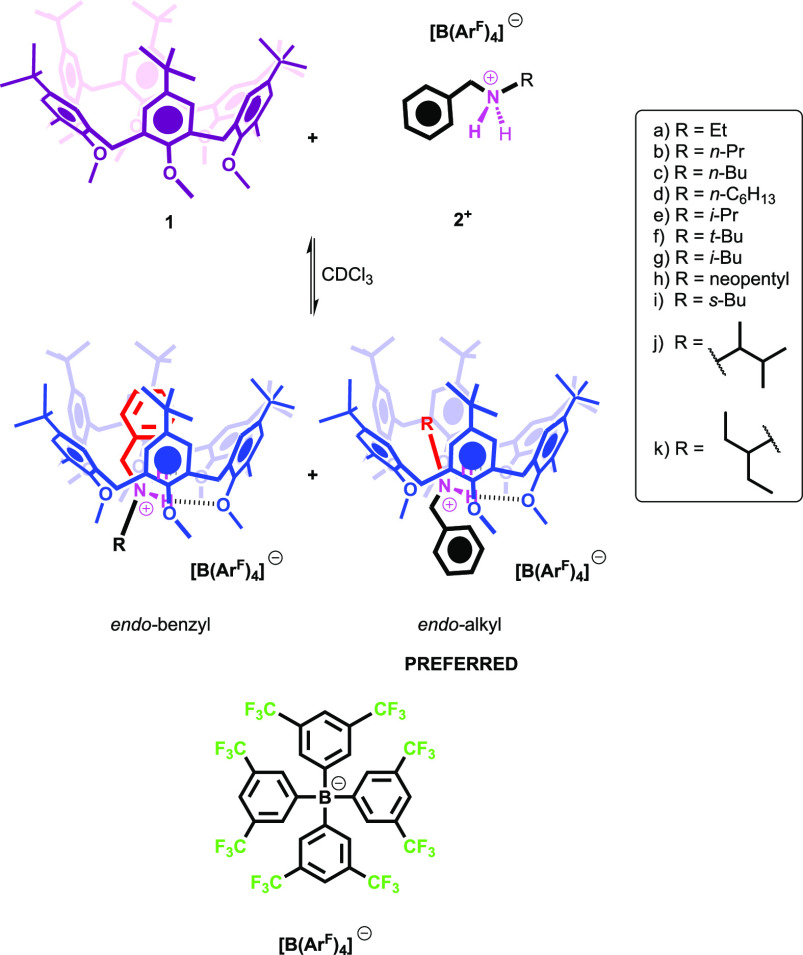
Threading of Calix[6]arene **1** with Alkylbenzylammonium Axles **2a**–**k**^+^

The aromatic walls of receptor **1** are freely rotating through the macrocyclic annulus, and thus they adapt their relative orientation to give the best interactional fitting with guest **2**. As a result, a less-symmetrical induced-fit complex is obtained in which each aromatic ring is differently inclined toward the cavity (*vide infra*). Since the PhCH_2_NH_2_^+^ moiety is common to all the **2a**–**k**^**+**^ axles and since it is external to the aromatic calix-cavity, we can assume that the stability constant of **2^+^**⊂**1** pseudorotaxanes could be directly linked to the best interactional fitting of the alkyl moiety of **2** inside the aromatic cavity of **1**.

Based on these considerations, the question arises as to whether the shape and dimension of the hosted alkyl moiety can influence the effectiveness of complexation: is there any cavity-filling effect? Is there a maximum or an optimal filling? Is there any quantitative parameter in suitable agreement with the experimental results?

The threading abilities of alkylbenzylammonium cations **2a**–**k**^**+**^ toward calix[6]arene **1** (Scheme 1) were studied by ^1^H NMR titration experiments (CDCl_3_, 298 K) by mixing equimolar quantities of **1** and **2** in CDCl_3_ (3.8 mM solution) (Figures S24–S45).^14a^ With all the axles **2a**–**k**^**+**^, the pseudo[2]rotaxane stereoisomer with *endo*-alkyl stereochemistry was preferentially formed (see the Supporting Information for further details), following the *endo*-alkyl rule^14e^ previously reported. The apparent association constants (Table 1) for the formation of pseudo[2]rotaxanes **2a**–**k**^**+**^⊂**1** were determined by integration of the slowly exchanging ^1^H NMR signals for both free and complexed hosts (see Supporting Information (SI)).^15^ In some instances, competition experiments with known pseudo[2]rotaxanes were also used (see SI).

**Table 1:** Apparent Association Constants of **2**^+^⊂**1** Pseudorotaxanes and Their PC, CC, and EC Parameters

Axle	*K*_app_ (M^–1^)^a^	log *K*_app_	PC (%)	CC (%)	EC	Δ*G*_Reorg_ (kcal/mol)
**2a^+^**⊂**1**	(1.1 ± 0.2) × 10^6^	6.04	72	70	5.5	19.2
**2b^+^**⊂**1**	(4.8 ± 0.8) × 10^3^	3.68	100	72	7.2	28.0
**2c^+^**⊂**1**	(6.5 ± 0.9) × 10^4^	4.81	88	74	8.0	28.8
**2d^+^**⊂**1**	(2.4 ± 0.6) × 10^3^	3.38	94	71	10.1	32.0
**2e^+^**⊂**1**	(4.2 ± 0.6) × 10^4^	4.62	74	71	7.2	25.5
**2f^+^**⊂**1**	(3.6 ± 0.5) × 10^2^	2.56	82	77	9.5	36.9
**2g^+^**⊂**1**	(5.1 ± 0.6) × 10^3^	3.71	88	74	9.5	37.6
**2h^+^**⊂**1**	(1.7 ± 0.6) × 10^3^	3.23	86	75	10.8	31.9
**2i^+^**⊂**1**	(6.9 ± 0.8) × 10^3^	3.84	89	75	8.4	31.6
**2j^+^**⊂**1**	(2.9 ± 0.5) × 10^3^	3.46	87	76	10.5	35.3
**2k^+^⊂1**	(2.7 ± 0.4) × 10^2^	2.43	97	80	11.6	41.1

a The apparent association constant values were determined by mixing equimolar quantities of host and guest in CDCl_3_ (3.8 mM solution each, 298 K) by using the following methods: (i) ^1^H NMR competition experiment (**2a**^**+**^, **2b**^**+**^, **2c**^**+**^, and **2e**^**+**^); (ii) quantitative ^1^H NMR experiment using 1,1,2,2-tetrachloroethane as the internal standard (**2k**^**+**^); (iii) integration of free and complexed ^1^H NMR signals of host or guest (**2d**^**+**^, **2f**^**+**^, **2g**^**+**^, **2h**^**+**^, **2i**^**+**^, and **2j**^**+**^).

At this point, to test the initial hypothesis, the log *K*_app_ data reported in Table 1 were correlated with the PC of the calix-cavity in pseudo[2]rotaxanes **2a**–**k**^**+**^⊂**1** obtained using the DFT-optimized structures at the B3LYP-D3/6-31G(d,p) level of theory (D3 stands for Grimme’s dispersion correction energy term^16^ and has been already used for DFT calculations in calixarene-based pseudorotaxane structures^17^). As expected, close inspection of the data reported in Table 1 revealed that the PC of the calix-cavity in pseudo[2]rotaxane **2a**–**k**^**+**^⊂**1** structures overcomes the above-mentioned 55% rule (*PC* range = 72–100%) and the correlation coefficient of the linear fitting is low (*R*^2^ = 0.38, Figure S46), indicating that there is a low correlation between the two parameters.

In Figure 1a–c, the superimposed calix[6]arene-wheels of the optimized structures **2**^**+**^⊂**1** are reported (see also Figure S47). Inspection of Figure 1a reveals that the calix[6]arene-wheels adopt two main conformations upon complexation: the cone-1,3,5-out (**2a**–**c**^**+**^⊂**1** and **2e**–**j**^**+**^⊂**1**; see Figure 1b) and the cone-1,4-out (**2d**^**+**^⊂**1** and **2k**^**+**^⊂**1**; see Figure 1c). Moreover, it is evident that, during the induced-fit recognition process, the calix-cavity deforms, allowing the change of the void volume of the receptor.

**Figure 1:**
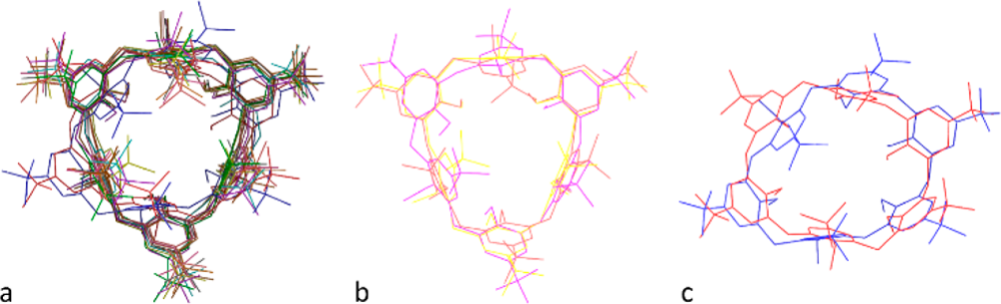
DFT-optimized structures, at B3LYP-D3/6-31G(d,p) level of theory, of the following: (a) superimposed calix[6]arene-wheels of **2**^**+**^⊂**1** pseudorotaxanes (global minimum); (b) only **2a**^**+**^⊂**1**, **2b**^**+**^⊂**1**, and **2j**^**+**^⊂**1**; (c) only **2d**^**+**^⊂**1** and **2k**^**+**^⊂**1**.

Close inspection of Figure 1b,c reveals a remarkable change of inclination of all the aromatic rings (−21° from yellow to coral, and +23° from yellow to purple, on average, respectively) of **1**, in pseudo[2]rotaxanes **2a**^**+**^⊂**1**, **2b**^**+**^⊂**1**, and **2k**^**+**^⊂**1**. Thus, the conformational freedom of **1** ensures the best fit around the alkyl portion of guest **2a**^**+**^, **2b**^**+**^, or **2k**^**+**^ to establish an extended area of contact between them. In other words, the aromatic walls of calixarene host **1** move to wrap the guest and maximize the secondary interactions with it.

From the above data, it is evident that another useful and straightforward rule is necessary to assess the stability of these induced-fit complexes in order to predict the ideal cavity-filling effect. Initially, we reasoned that the maximization of weak secondary interactions should be parallel to the maximization of the contact surface between host and guest; therefore, we studied a new surface-based single-value parameter to address such point. With this aim, we considered the *Contacting Coefficient* (CC, eq 1) defined as the ratio between the molecular surface of the guest in close contact with the cavity surface (*S*_Contact_) of the host, and the total surface of the guest (*S*_Guest_).
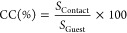
1This new parameter does not consider the host cavity volume and could be applied to host–guest processes that follow the induced-fitting mechanism. In addition, the CC parameter should be more directly related to the thermodynamic stability of the complex because it considers the host–guest contacting surface, which should be related to the extension of van der Waals and C–H···π interactions between them.

Starting from the complexes’ DFT-optimized structures, the molecular surfaces of the guest inside the cavity (*S*_Guest_) were computed by YASARA software, which also permits the direct measure of the contact surface between guest and host (*S*_Contact_). From the ratio of those surfaces, CC (%) values of 70, 74, and 80 were calculated, through eq 1, for **2a**^**+**^⊂**1**, **2c**^**+**^⊂**1**, and **2k**^**+**^⊂**1** pseudo[2]rotaxanes (Table 1), respectively. The *S*_contact_ is represented in red in Figure S48, while the *S*_Guest_ indicates the total molecular surface of the guest. Close inspection of Figure S48 reveals that, in addition to the *S*_contact_ (in red), there are free portions of the guest’s molecular surface not in contact with the calixarene cavity (in yellow).

Unfortunately, the CC of the whole set has only a discrete correlation coefficient (*R*^2^ = 0.54, Figure S49).

At this point, we decided to evaluate another single-value geometrical parameter, which could take more directly into account the energy cost associated with the host reorganization upon induced-fit complexation. Therefore, we considered the *Expanding Coefficient* (EC, eq 2) defined as the ratio between the final and the initial cavity volumes of the host, i.e., the volume of the host cavity after the complexation (*V*_complexed___Host_ at the global minimum) and that of the host cavity before the complexation (*V*_free___Host_ at the global minimum).
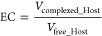
2The actual values of *V*_complexed_Host_ and *V*_free_Host_ were measured by using the DFT-optimized structures of the separated host and guest for all the **2**^**+**^⊂**1** complexes (see the SI)^18^ with the Caver software. From these values, the corresponding ECs were then calculated (Table 1), and a linear regression analysis was performed with the pertinent log *K*_app_ data. As shown in Figure 2, a good correlation coefficient (*R*^2^ = 0.74) was obtained, demonstrating good linearity between the new EC parameter and the complex’s thermodynamic stability. It is evident that the EC parameter is now less affected than CC by the structural differences of the variously branched alkyl chains of **2a**–**k**^**+**^.

**Figure 2:**
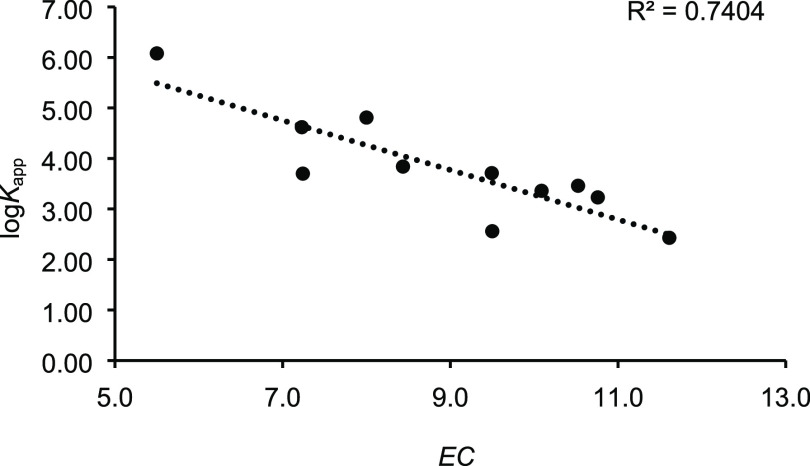
Linear regression analysis of ECs vs log *K*_app_ values for **2**^**+**^⊂**1** alkylbenzylammonium-based pseudorotaxane complexes.

The good correlation performance of the geometrical EC parameter induced us to consider its possible physical meaning. In fact, this EC can be considered an approximate geometrical measure of the energy cost paid by the host when it reorganizes itself from the initial lowest-energy conformation to the final geometry adopted in the complex. A higher EC value implies a higher deformation of the host, which in turn implies a higher energetical cost. From another point of view, the EC can be considered an approximate indirect inverse measurement of the preorganization of the host for the complexation of the given guest. The importance of this reorganization cost was first recognized by Cram,^3c^ who stated that “preorganization is a central determinant of binding power” leading to the formalization of the *Principle of Preorganization*, which states that “the more highly hosts and guests are organized for binding, and the lower the solvation before their complexation, the more stable will be their complexes”.

To verify the correctness of this “reorganization” point of view, we decided to calculate the energy of the host **1** in its bound conformation by performing a single-point calculation on each **2**^**+**^⊂**1** complex after taking away the **2**^**+**^ guest. The difference between this single-point bound conformation energy and the lowest energy of **1** gives a *ΔG*_Reorg_ value, which can be considered the theoretically computed energetical cost for the above-mentioned reorganization of the host, from the initial lowest-energy conformation to the final geometry adopted in the complex. The *ΔG*_Reorg_ values computed for all the **2a**–**k**^**+**^⊂**1** complexes are reported in Table 1. Interestingly, these data seem to be in accord with the “reorganization” point of view, and indeed a good linear correlation (*R*^2^ = 0.79) was found by regression analysis between *ΔE*_Reorg_ vs log *K*_app_ values (Figure S50). In summary, this analysis indicates that, under the above assumption of weak intermolecular interactions (dispersion forces, van der Waals interactions), the primary determinant to the stability of induced-fit complexes will be the degree of deformation with respect to the ground conformation.

In conclusion, we have defined the EC new parameter that can be correlated with the thermodynamic stability of supramolecular complexes governed by weak secondary interactions that obey the induced-fit model. The EC values show a good linear relationship with the log *K*_app_ of the respective pseudorotaxane complexes. This EC can be considered an approximate mechanical measure of the reorganization energy cost paid by the host upon changing from the initial free lowest-energy conformation to the final bound geometry in the complex. This conclusion is in accordance with the Principle of Preorganization, by which the reorganization cost is a central determinant of binding power. We believe that the EC parameter can be of general applicability in all those instances in which no new strong intermolecular interactions (e.g., H-bonds) are generated during the induced-fit process.^19^ We anticipate future studies to test the ECs applicability in different systems, including the biological ones.

## Supporting Information Available

Experimental details, characterization data, NMR spectra, calculations details, and Cartesian coordinates (PDF)
