# Supplementary material for: Expanding Coefficient: A Parameter To Assess the Stability of Induced-Fit Complexes
Source: Org Lett. 2021 Feb 16;23(5):1804–8. doi: 10.1021/acs.orglett.1c00165 (PMC8028309; doi:10.1021/acs.orglett.1c00165)
Supplement: Supplementary file 1 — ol1c00165_si_001.pdf [file ol1c00165_si_001.pdf]

Supporting Information for

Expanding Coefficient: A Parameter to Assess the Stability of Induced-Fit Complexes

Carmen Talotta,[†,*] Gerardo Concilio,[†] Margherita De Rosa,[†] Annunziata Soriente,[†] Carmine Gaeta,[†] Antonio Rescifina,[‡,*] Pablo Ballester,[‖,*] and Placido Neri[†,*]

[†] Dipartimento di Chimica e Biologia "A. Zambelli", Università di Salerno, via Giovanni Paolo II, 132, I-84084, Fisciano (Salerno), Italy

[‡] Dipartimento di Scienze del Farmaco e della Salute, Università di Catania, viale Andrea Doria, 6, I-95125 Catania, Italy

[‖] Institute of Chemical Research of Catalonia (ICIQ), The Barcelona Institute of Science and Technology (BIST), Av. Països Catalans, 16, 43007-Tarragona, Spain.

Table of Contents

Experimental Procedures S4
General comments S4
Chart S1 S5
Synthesis of **$2a^{+}$·[B(Ar^{F})$_4$]$^{-}$** S6
^{1}H and ^{13}C NMR spectra of **$2a^{+}$·[B(Ar^{F})$_4$]$^{-}$** S8
Synthesis of **$2b^{+}$·[B(Ar^{F})$_4$]$^{-}$** S10
^{1}H and ^{13}C NMR spectra of **$2b^{+}$·[B(Ar^{F})$_4$]$^{-}$** S12
Synthesis of **2c-g,i^{+}·[B(Ar^{F})$_4$]$^{-}$** S14
^{1}H and ^{13}C NMR spectra of **$2c^{+}$·[B(Ar^{F})$_4$]$^{-}$** S18
^{1}H and ^{13}C NMR spectra of **$2d^{+}$·[B(Ar^{F})$_4$]$^{-}$** S20
^{1}H and ^{13}C NMR spectra of **$2e^{+}$·[B(Ar^{F})$_4$]$^{-}$** S22
^{1}H and ^{13}C NMR spectra of **$2f^{+}$·[B(Ar^{F})$_4$]$^{-}$** S25
^{1}H and ^{13}C NMR spectra of **$2g^{+}$·[B(Ar^{F})$_4$]$^{-}$** S27
^{1}H and ^{13}C NMR spectra of **$2i^{+}$·[B(Ar^{F})$_4$]$^{-}$** S29
Synthesis of **$2h^{+}$·[B(Ar^{F})$_4$]$^{-}$** S31
^{1}H and ^{13}C NMR spectra of **$2h^{+}$·[B(Ar^{F})$_4$]$^{-}$** S33
Synthesis of **2j,k^{+}·[B(Ar^{F})$_4$]$^{-}$** S35
^{1}H and ^{13}C NMR spectra of **$2j^{+}$·[B(Ar^{F})$_4$]$^{-}$** S37
^{1}H and ^{13}C NMR spectra of **$2k^{+}$·[B(Ar^{F})$_4$]$^{-}$** S39
General procedure for the preparation of Pseudo[2]rotaxanes **2a–k^{+}⊂1** S41
Scale-up of procedure for the preparation of Pseudo[2]rotaxane **$2a^{+}$⊂1** S41
^{1}H NMR Spectrum of **$2a^{+}$⊂ 1** S42
^{1}H NMR Spectrum of **$2b^{+}$⊂ 1** S43
^{1}H NMR Spectrum of **$2c^{+}$⊂ 1** S44
^{1}H NMR Spectrum of **$2d^{+}$⊂ 1** S45
^{1}H NMR Spectrum of **$2e^{+}$⊂ 1** S46
^{1}H NMR Spectrum of **$2f^{+}$⊂ 1** S47
^{1}H NMR Spectrum of **$2g^{+}$⊂ 1** S48
^{1}H NMR Spectrum of **$2h^{+}$⊂ 1** S49
^{1}H NMR Spectrum of **$2i^{+}$⊂ 1** S50
^{1}H NMR Spectrum of **$2j^{+}$⊂ 1** S51
^{1}H NMR Spectrum of **$2k^{+}$⊂ 1** S52
^{1}H NMR determination of K_{ass} values S53
Apparent association constants of **2a-k^{+}⊂1** pseudorotaxanes S54
K_{ass} value of **$2a^{+}$⊂ 1** S55
K_{ass} value of **$2b^{+}$⊂ 1** S56
K_{ass} value of **$2c^{+}$⊂ 1** S57

K_{ass} value of **2d**$^{+}\subset$ **1** S58
K_{ass} value of **2e**$^{+}\subset$ **1** S59
K_{ass} value of **2f**$^{+}\subset$ **1** S60
K_{ass} value of **2g**$^{+}\subset$ **1** S61
K_{ass} value of **2h**$^{+}\subset$ **1** S62
K_{ass} value of **2i**$^{+}\subset$ **1** S63
K_{ass} value of **2j**$^{+}\subset$ **1** S64
K_{ass} value of **2k**$^{+}\subset$ **1** S65
Computational details S67
Cartesian coordinates of the full optimized structures for the complexes **2a–k**$^{+}\subset$**1** S71

Experimental Procedures

General comments

ESI(+)-MS measurements were performed on a Micromass Bio-Q triple quadrupole mass spectrometer equipped with electrospray ion source, using a mixture of H_2O/CH_3CN (1:1) and 5% HCOOH as solvent. Flash chromatography was performed on Merck silica gel (60, 40-63 □m). All chemicals were reagent grade and were used without further purification. Anhydrous solvents were purchased from Aldrich. When necessary compounds were dried in vacuo over $CaCl_2$. Reaction temperatures were measured externally. Reactions were monitored by TLC on Merck silica gel plates (0.25 mm) and visualized by UV light, or by sprying with H_2SO_4-$Ce(SO_4)_2$ or phosphomolybdic acid. Derivative **1** and sodium tetrakis[3,5-bis(trifluoromethyl)phenyl]borate ($[B(Ar^F)_4]Na$)[1] were synthesized according to literature procedures and they are not reported here in this section. 1D NMR spectra were recorded on a Bruker Avance-400 spectrometer [400 (^{1}H) and 100 MHz (^{13}C)], Bruker Avance-300 spectrometer [300 (1H) and 75 MHz (^{13}C)], and Bruker Avance-250 spectrometer [250 (1H) and 63 MHz (^{13}C)]; chemical shifts are reported relative to the residual solvent peak ($C\mathit{H}Cl_3$: □ 7.26, $\mathit{C}DCl_3$: □ 77.23; $CD_3O\mathit{H}$: □ 4.87, $\mathit{C}D_3OD$: □ 49.0).

[1] H. Nishida, N. Takada, M. Yoshimura, T. Sonoda, H. Kobayashi, *Bull. Chem. Soc. Jpn.* **1984**, *57*, 2600-2604.

Chart S1

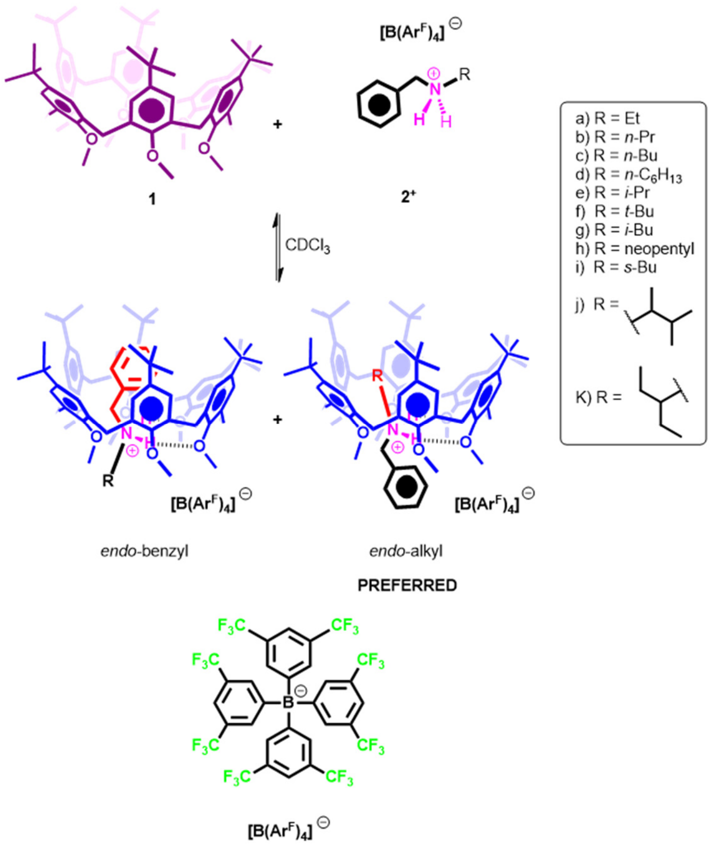

Chart S1. Threading of alkylbenzylammonium axles **2a–k**$^{+}$ with calix[6]arene macrocycle **1**. **[B(Ar^{F})$_4$]$^{-}$**: Tetrakis[3,5-bis(triFluoromethyl)Phenyl]Borate.$^{-}$

Synthesis of 2a⁺·[B(Ar^F)$_4$]⁻

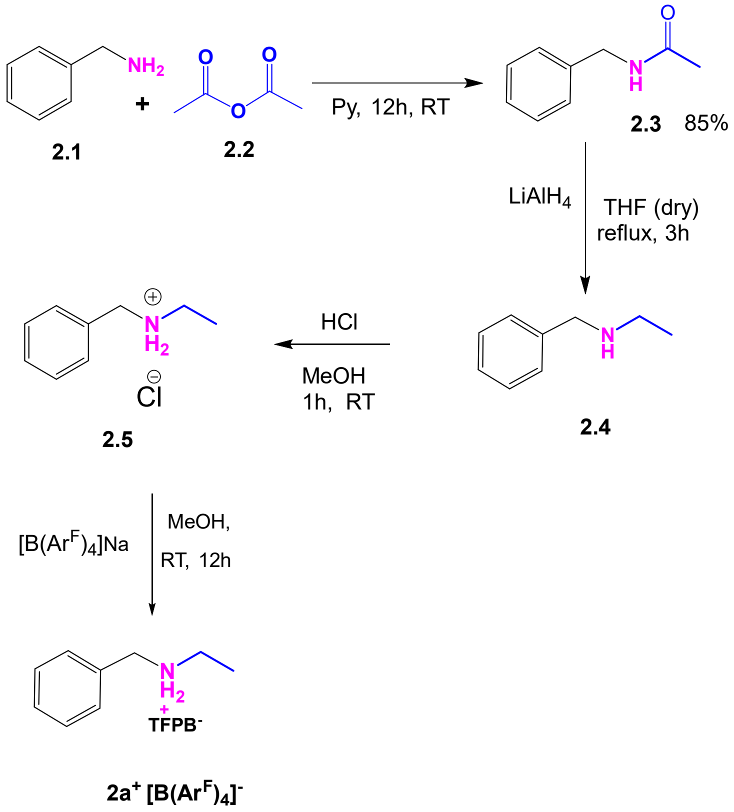

Scheme S1. Synthesis of **2a⁺·[B(Ar^F)$_4$]⁻**

To benzylamine **2.1** (10.0 mmol) was added acetic anhydride **2.2** (10.0 mmol) and pyridine (0.50 mL) and the reaction mixture was kept overnight under stirring at room temperature. The excess of anhydride was removed under reduced pressure to give compound **2.3** (1.2 g; 85%). In a three necked flask and under nitrogen atmosphere, compound **2.3** (7.7 mmol) was dissolved in anhydrous THF (70 mL) and $LiAlH_4$ (18.0 mmol) was added at 0° C. The resulting mixture was stirred at room temperature for 2 h and at reflux for 2 h. The reaction was quenched by pouring the mixture in 1.0 *N* HCl (0.10 L) and the organic material was extracted with AcOEt. The organic layer was washed with water, dried over $MgSO_4$ and the solvent was removed under reduced pressure to give derivative **2.4** as a yellow viscous liquid. The crude product (8.0 mmol) was dissolved in Et_2O (20 mL) at room temperature and an aqueous solution of HCl (37% w/w, 8.0 mmol) was added dropwise. The mixture was kept under stirring for 1 h, until the formation of a white precipitate. The solid was collected by filtration, purified by crystallization with hexane/MeOH and dried under vacuum, to give derivative **2.5** as a white solid.

Derivative **2.5** (0.15 mmol) was dissolved in dry MeOH (5.0 mL), then sodium tetrakis[3,5-bis(trifluoromethyl)phenyl]borate (0.16 mmol) was added and the mixture was kept under stirring overnight in the dark. The solvent was removed and deionized water was added, obtaining a brown precipitate that was filtered off and dried under vacuum to give **$2a^{+}$·[B(Ar^{F})$_4$]$^{-}$.**

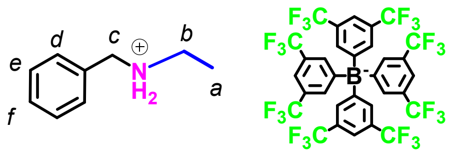

2a^{+}·[B(Ar^{F})$_4$]$^{-}$

2a^{+}·[B(Ar^{F})$_4$]$^{-}$: (yield: 0.1310 g, 0.13 mmol, 80% calculated with respect to derivative **2.5**). **ESI(+) MS**: m/z = 136.14 (M^{+}). **^{1}H NMR** (400 MHz, $CDCl_3$, 298 K): □ 7.61 (s, 8H, $ArH^{[B(ArF)_4]^-}$), 7.54 (s, 4H, $ArH^{[B(ArF)_4]^-}$), 7.51 (d, J = 7.43 Hz, 1H, H_f), 7.44 (d, J = 7.32 Hz, 2H, H_e), 7.21 (d, J = 7.43 Hz, 2H, H_d), 4.06 (bs, 2H, H_c), 3.06 (bs, 2H, H_b), 1.24 (t, J = 7.22 Hz, 3H, H_a); **^{13}C NMR** (100 MHz, CD_3OD, 298 K): □ 162.2, 161.7, 161.2, 160.7, 134.4, 131.2, 129.5, 129.4, 129.3, 129.2, 128.9, 128.6, 128.4, 125.7, 123.0, 120.3, 117.1, 50.6, 42.4, 10.0. Anal. Calcd for $C_{41}H_{26}BF_{24}N$: C 49.27, H 2.62, N 1.40. Found: C 49.26, H 2.61, N 1.41

.

^{1}H and ^{13}C NMR spectra of 2a^{+}·[B(Ar^{F})$_4$]$^{-}$

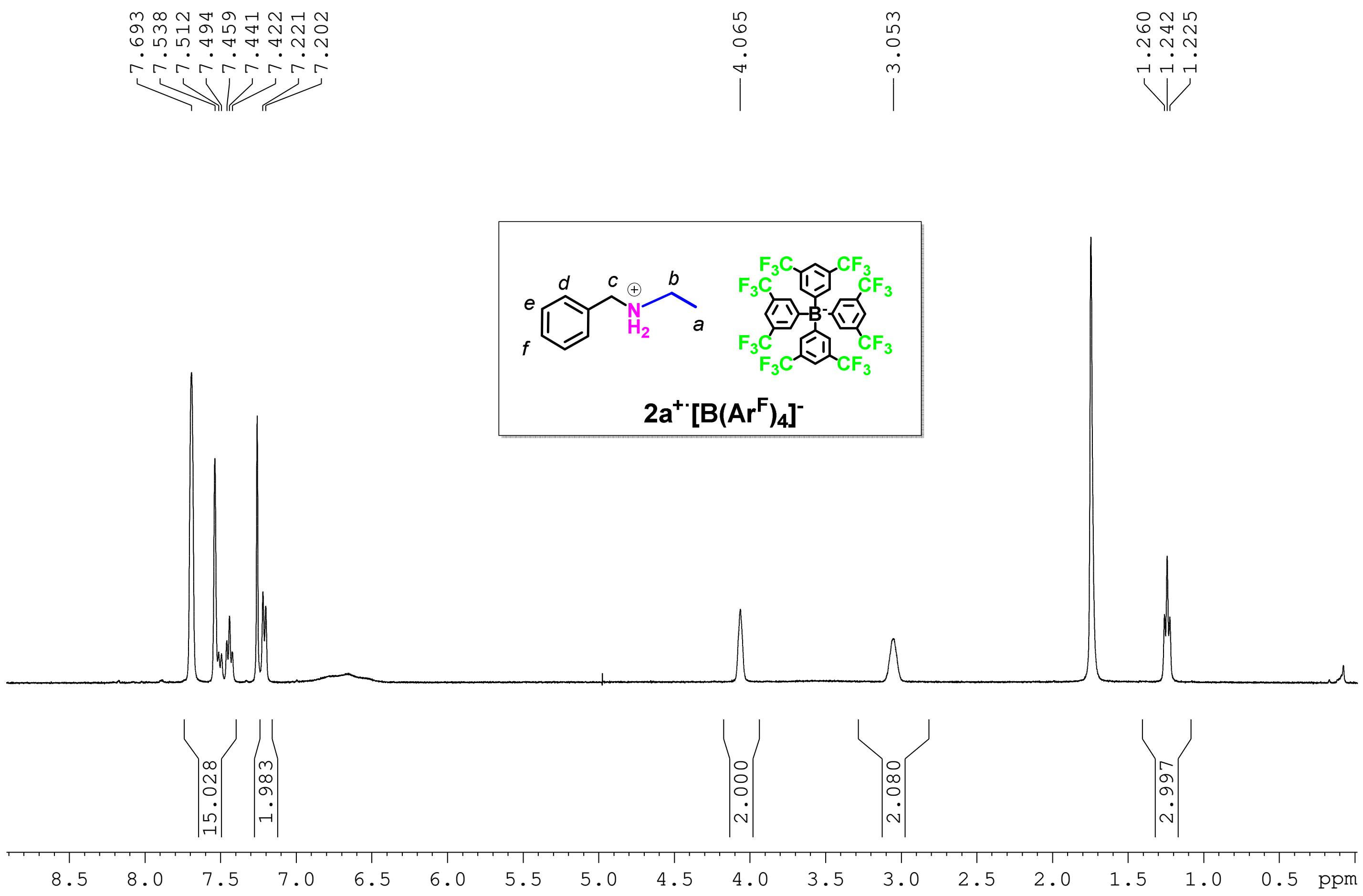

Figure S1. ^{1}H NMR spectrum of **2a^{+}·[B(Ar^{F})$_4$]$^{-}$** (400 MHz, $CDCl_3$, 298 K).

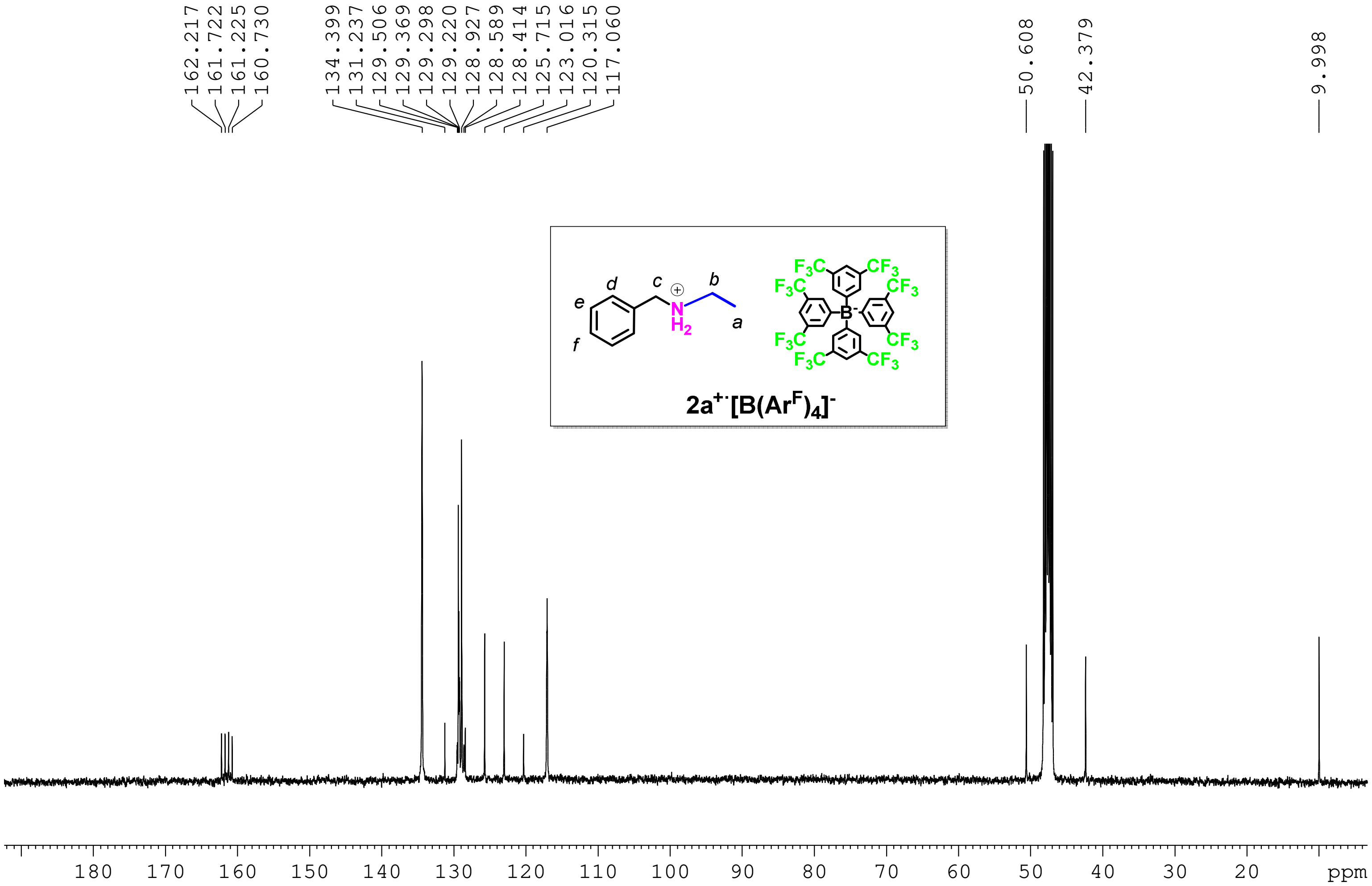

Figure S2. ^{13}C NMR spectrum of **$2a^{+}$·[B(Ar^{F})$_4$]$^{-}$** (100 MHz, CD_3OD, 298 K).

Synthesis of $2b^{+}\cdot[B(Ar^{F})_4]^{-}$

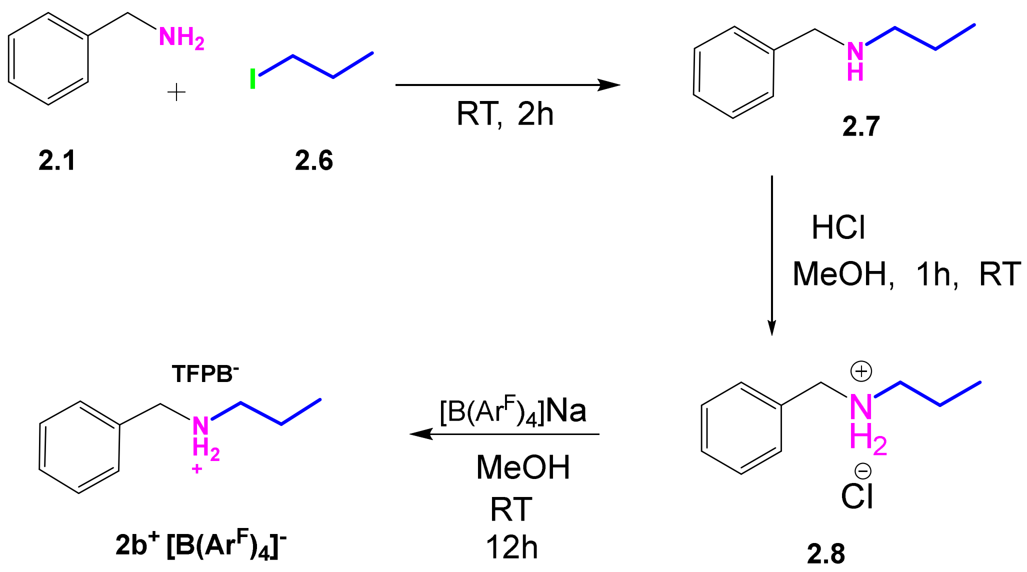

Scheme S2. Synthesis of derivative **$2b^{+}\cdot[B(Ar^{F})_4]^{-}$**

To benzylamine **2.1** (10.0 mmol) was added 1-iodopropane (10.0 mmol) and the reaction mixture was stirred at room temperature for 2 h. Then, unreacted benzylamine was removed by crystallization with CH_2Cl_2. The resulting crude product was subjected to flash chromatography on silica gel ($CHCl_3$/MeOH, 98/2) to give the ammonium iodide intermediate (1.7 g, 4.2 mmol, 42%).

The ammonium iodide (1.7 g, 4.2 mmol) was dissolved in AcOEt (30 mL) and 1.0 *N* KOH aqueous solution (30 mL) and the resulting mixture was stirred at room temperature for 4 h to give amine **2.7**.

The amine (2.0 mmol) was dissolved in Et_2O (200 mL) at room temperature and an aqueous solution of HCl (37% w/w, 1.2 eq) was added dropwise. The mixture was kept under stirring for 1 h, until the formation of a white precipitate. The solid was collected by filtration, purified by crystallization with acetonitrile and dried under vacuum, to give derivative **2.8** as a white solid.

Derivative **2.8** (1 eq.) was dissolved in dry MeOH (C = 0.20 M), then sodium tetrakis[3,5-bis(trifluoromethyl)phenyl]borate (1.1 eq.) was added and the mixture was kept under stirring overnight in the dark. The solvent was removed and deionized water was added, obtaining a brown precipitate that was filtered off and dried under vacuum to give **$2b^{+}\cdot[B(Ar^{F})_4]^{-}$**.

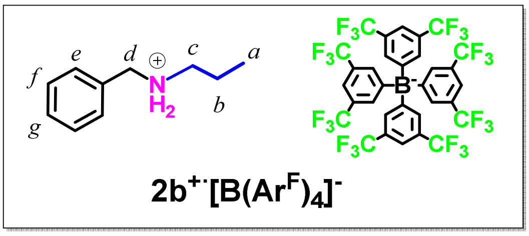

2b⁺·[B(ArF)4]⁻: (yield: 1.3 g, 1.3 mmol, 78% calculated with respect to derivative **2.8**). **ESI(+) MS**: *m*/*z* = 150.14 (M^+). **^{1}H NMR** (300 MHz, CD_3OD, 298 K): δ 7.61 (overlapped, 12H, ArH$^{[B(ArF)_4]^-}$), 7.48 (overlapped, 5H, $H_{e\text{-}g}$), 4.20 (s, 2H, H_d), 3.00 (t, *J* = 8 Hz, 2H, H_c), 1.73 (sext, *J* =7.2 Hz 2H, H_b), 1.03 (t, *J* =7.5 Hz, 3H, H_a); **^{13}C NMR** (75 MHz, CD_3OD, 298 K): δ 162.6, 161.9, 161.3, 160.6, 134.5, 131.3, 129.9, 129.6, 129.5, 129.1, 126.3, 122.7, 119.1, 51.1, 48.9, 19.3. Anal. Calcd for $C_{42}H_{28}BF_{24}N$: C 49.78, H 2.78, N 1.38. Found: C 49.77, H 2.79, N 1.39.

^{1}H and ^{13}C NMR spectra of 2b⁺·[B(Ar^F)₄]⁻

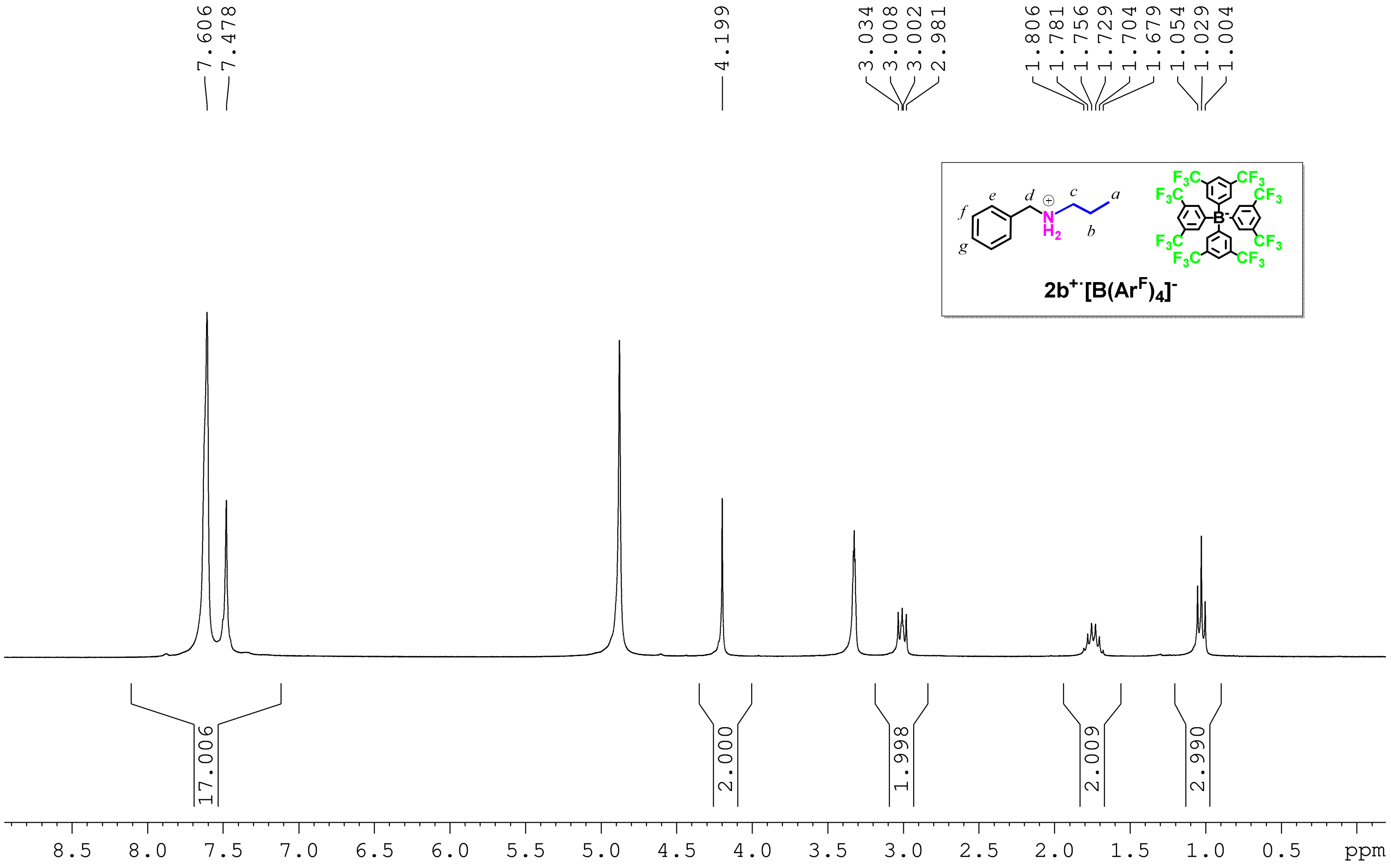

Figure S3. ^{1}H NMR spectrum of **2b^{+}·[B(Ar^{F})$_4$]$^{-}$** (300 MHz, CD_3OD, 298 K).

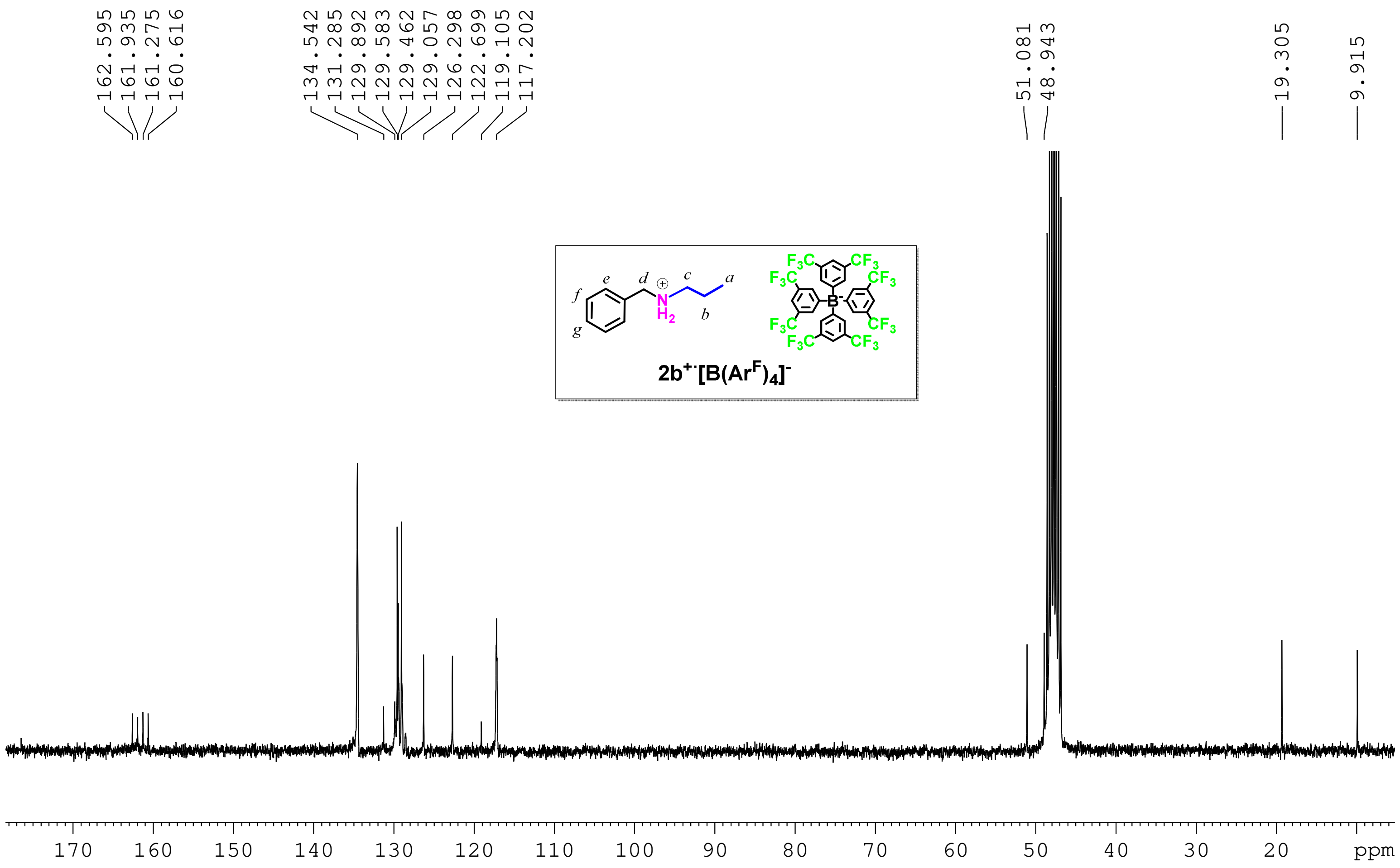

Figure S4. ^{13}C NMR spectrum of **$2b^{+}\cdot[B(Ar^{F})_4]^{-}$** (75 MHz, CD_3OD,298 K).

Synthesis of 2c-g,i^{+}·[B(Ar^{F})$_4$]$^{-}$

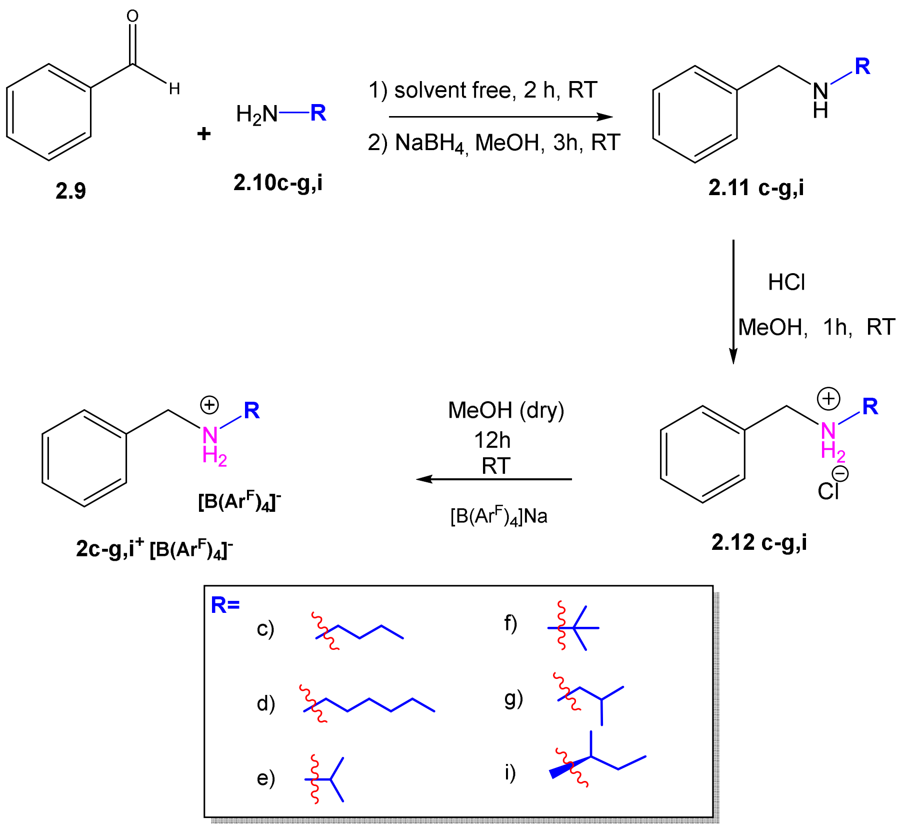

Scheme S3. Synthesis of **2c-g,i $^{+}$·[B(Ar^{F})$_4$]$^{-}$**

To benzaldehyde (10.0 mmol) was added the appropriate amine (10.0 mmol). The reaction mixture was stirred at room temperature for 2 h to give the imine intermediate in a quantitative yield. The imine was used for the next step without further purification. The imine (10.0 mmol) was dissolved in dry MeOH (20 mL) under a nitrogen atmosphere and $NaBH_4$ (10.0 mmol) was added at 0 °C and then the mixture was allowed to warm at room temperature. The solution was kept under stirring for 3 h. The solvent was removed under reduced pressure and the residue partitioned between AcOEt and an aqueous saturated solution of $NaHCO_3$. The organic layer was dried over $MgSO_4$ and the solvent was removed, under reduced pressure, to give the secondary amine as a yellow viscous liquid. The amine was used for the next step without further purification. The crude product (10.0 mmol) was dissolved in Et_2O (20 mL) at room temperature and an aqueous solution of HCl (37% w/w, 10.0 mmol) was added dropwise. The mixture was kept under stirring for 1 h, until the formation of a white

precipitate. The solid was collected by filtration, purified by crystallization with acetonitrile and dried under vacuum, to give the corresponding chloride as a white solid. The corresponding chloride **2.12c-g,i** (2.0 mmol) was dissolved in dry MeOH (C = 0.20 M), then sodium tetrakis[3,5-bis(trifluoromethyl)phenyl]borate (1.1 eq) was added and the mixture was kept under stirring overnight in the dark. The solvent was removed and deionized water was added, obtaining a brown precipitate that was filtered off and dried under vacuum to give the corresponding salt **2c-g,i^{+}·[B(Ar^{F})$_4$]$^{-}$**.

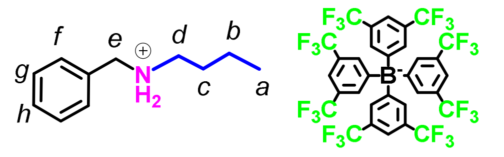

2c^{+}·[B(Ar^{F})$_4$]$^{-}$

2c^{+}·[B(Ar^{F})$_4$]$^{-}$: (yield: 1.91 g, 1.86 mmol, 93% calculated with respect to derivative **2.12c**). **ESI(+) MS**: *m/z* = 164.16 (M^{+}). **^{1}H NMR** (400 MHz, $CDCl_3$, 298 K): □ 7.61 (s, 8H, $ArH^{[B(ArF)_4]^-}$), 7.54 (s, 4H, $ArH^{[B(ArF)_4]^-}$), 7.51 (d, *J* = 7.59 Hz, 1H, H_h), 7.44 (d, *J* = 7.70 Hz, 2H, H_g), 7.21 (d, *J* = 6.57 Hz, 2H, H_f), 4.12 (bs, 2H, H_e), 3.08 (bs, 2H, H_d), 1.61 (bm, 2H, H_c), 1.30 (bm, 2H, H_b), 0.88 (t, *J* = 7.22 Hz, 3H, H_a); **^{13}C NMR** (100 MHz, $CDCl_3$, 298 K): □ 162.2, 161.7, 161.2, 160.7, 134.4, 131.2, 129.5, 129.4, 129.3, 129.2, 128.9, 128.6, 128.4, 125.7, 123.0, 120.3, 117.1, 50.6, 42.4, 10.0. Anal. Calcd for $C_{43}H_{30}BF_{24}N$: C 50.27, H 2.94, N 1.36. Found: C 50.26, H 2.93, N 1.37.

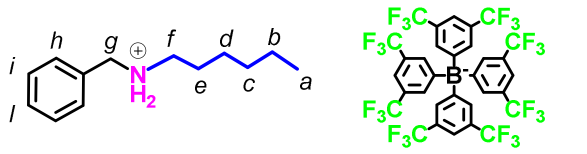

2d^{+}·[B(Ar^{F})$_4$]$^{-}$

2d^{+}·[B(Ar^{F})$_4$]$^{-}$: (yield: 2.09 g, 1.98 mmol, 99% calculated with respect to derivative **2.12d**). **ESI(+) MS**: *m/z* = 192.18 (M^{+}). **^{1}H NMR** (400 MHz, $CDCl_3$, 298 K): □ 7.61 (s, 8H, $ArH^{[B(ArF)_4]^-}$), 7.54 (s, 4H, $ArH^{[B(ArF)_4]^-}$), 7.51 (d, *J* = 7.59 Hz, 1H, H_l), 7.44 (d, *J* = 7.70 Hz, 2H, Hi_g), 7.21 (d, *J* = 6.57 Hz, 2H, H_h), 4.12 (bs, 2H, H_g), 3.08 (bs, 2H, Hf_d), 2.10 (bs, 4H, H_{d+e}), 1.61 (bm, 2H, H_c), 1.31 (bm, 2H, H_b), 0.88 (t, *J* = 7.22 Hz, 3H, H_a); **^{13}C NMR** (100 MHz, $CDCl_3$, 298 K): □ 162.4, 161.9, 161.4, 160.9, 134.8, 131.6, 130.4, 129.4, 129.1, 129.0, 128.8, 128.6, 128.4, 127.5, 125.9, 123.2, 120.4, 117.6, 117.6,

77.2, 53.6, 48.8, 28.2, 19.2, 3.0. Anal. Calcd for $C_{45}H_{34}BF_{24}N$: C 51.21, H 3.25, N 1.33. Found: C 51.20, H 3.24, N 1.34.

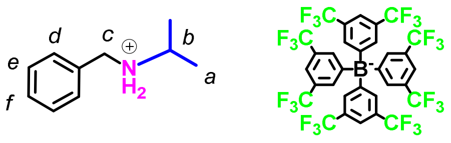

2e⁺·[B(Ar^F)₄]⁻

$2e^+$·[B(Ar^F)$_4$]$^-$: (yield: 1.78 g, 1.76 mmol, 88%, calculated with respect to derivative **2.12e**). **ESI(+) MS**: *m/z* = 150,15 (M^+). **^{1}H NMR** (300 MHz, CD_3OD, 298 K): □ 7.61 (overlapped, 12H, $ArH^{[B(ArF)_4]-}$), 7.48 (overlapped, 5H, H_{c-e}), 4.19 (s, 2H, H_c), 3.42 (m, 1H, H_b), 1.39 (d, *J* =6.5 Hz, 6H, H_a); **^{13}C NMR** (65 MHz, CD_3OD, 298 K): □ 162.8, 162.0, 161.2, 160.4, 134.5, 133.7, 131.7, 131.0, 129.5, 129.4, 129.3, 129.0, 128.9, 128.4, 126.7, 122.3, 118.0, 117.2, 114.2, 50.5, 18.0; **DEPT 135°** (75 MHz, CD_3OD, 298 K): □ 134.5, 129.4, 129.0, 117.2, 117.1, 50.4, 48.2, 17.9. Anal. Calcd for $C_{42}H_{28}BF_{24}N$: C 49.78, H 2.78, N 1.38. Found: C 49.79, H 2.79, N 1.37.

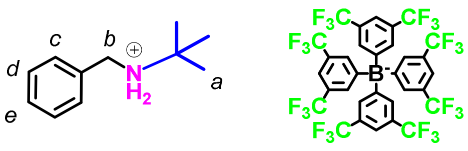

$2f^+$·[B(Ar^F)$_4$]$^-$

$2f^+$·[B(Ar^F)$_4$]$^-$: (yield: 1.81 g, 1.76 mmol, 88%, calculated with respect to derivative **2.12f**). **ESI(+) MS**: *m/z* = 164,15 (M^+). **^{1}H NMR** (400 MHz, CD_3OD, 298 K): □ 7.60 (overlapped, 12H, $ArH^{[B(ArF)_4]-}$), 7.48 (overlapped, 5H, H_{c-e}), 4.17 (s, 2H, H_b), 1.45 (s, 9H, H_a); **^{13}C NMR** (100 MHz, CD_3OD, 298 K): □ 162.2, 161.7, 161.2, 160.7, 134.4, 131.6, 129.5, 129.2, 129.0, 128.6, 128.4, 125.7, 123.0, 120.3, 117.1, 57.1, 45.2, 45.2. Anal. Calcd for $C_{43}H_{30}BF_{24}N$: C 50.27, H 2.94, N 1.36. Found: C 50.26, H 2.95, N 1.37.

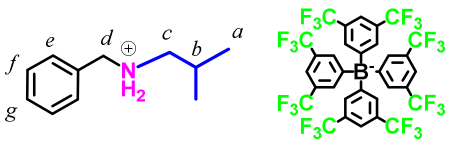

$2g^+$·[B(Ar^F)$_4$]$^-$

2g⁺·[B(ArF)4]⁻: (yield: 1.50 g, 1.46 mmol, 73%, calculated with respect to derivative **2.12g**). **ESI(+) MS**: *m/z* = 164,14 (M^+). **^{1}H NMR** (400 MHz, $CDCl_3$, 298 K): ◻ 7.69 (s, 8H, $ArH^{[B(ArF)_4]-}$), 7.53 (s, 4H, $ArH^{[B(ArF)_4]-}$), 7.50 (d, *J* = 7.89 Hz, 1H, H_g), 7.44 (t, *J* = 7.30 Hz, 2H, H_f), 7.21 (d, *J* = 7.59 Hz, 2H, H_e), 4.09 (bs, 2H, H_d), 2.86 (bm, 2H, H_c), 1.92 (bm, 1H, H_b), 0.94 (t, *J* = 6.25 Hz, 6H, H_a); **^{13}C NMR** (100 MHz, CD_3OD, 298 K): ◻ 162.2, 161.7, 161.2, 160.7, 134.4, 130.9, 129.6, 129.4, 129.2, 129.2, 128.9, 128.4, 125.7, 123.0, 120.3, 117.1, 117.1, 54.3, 51.4, 25.7, 18.9. Anal. Calcd for $C_{43}H_{30}BF_{24}N$: C 50.27, H 2.94, N 1.36. Found: C 50.28, H 2.93, N 1.36.

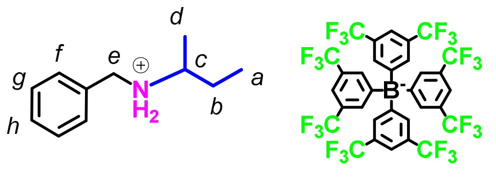

2i⁺·[B(ArF)4]⁻: (yield: 1.87g, 1.82 mmol, 91%, calculated with respect to derivative **2.12i**). **ESI(+) MS**: *m/z* =164.18 (M^+). **^{1}H NMR** (400 MHz, $CDCl_3$, 298 K): ◻ 7.69 (s, 8H, $ArH^{[B(ArF)_4]-}$), 7.42-7.53 (overlapped, 3H + 4H, Ar*H*+ $ArH^{[B(ArF)_4]-}$), 7.20 (d, *J* = 7, 2H, Ar*H*), 5.65-5.76 (m, broad, 2H, H_{NH2+}), 4.13 (m, broad, 2H, H_e), 3.30 (s, broad, 1H, H_c), 1.70 (overlapped, 1H, H_b), 1.60 (m, broad, 1H, H_b), 1.34 (d, *J* = 6.3 Hz, 3H, H_d), 0.94 (t, *J* = 7.2 Hz, 3H, H_a); **^{13}C NMR** (65 MHz, CD_3OD, 298 K): ◻ 161.3, 160.7, 159.8, 159.1, 133.0, 128.2, 127.9, 127.6, 125.1, 120.8, 115.8, 115.6, 54.3, 24.1, 13.0, 7.1. Anal. Calcd for $C_{43}H_{30}BF_{24}N$: C 50.27, H 2.94, N 1.36. Found: C 50.26, H 2.95, N 1.35.

^{1}H and ^{13}C NMR spectra of 2c^{+}·[B(Ar^{F})$_4$]$^{-}$

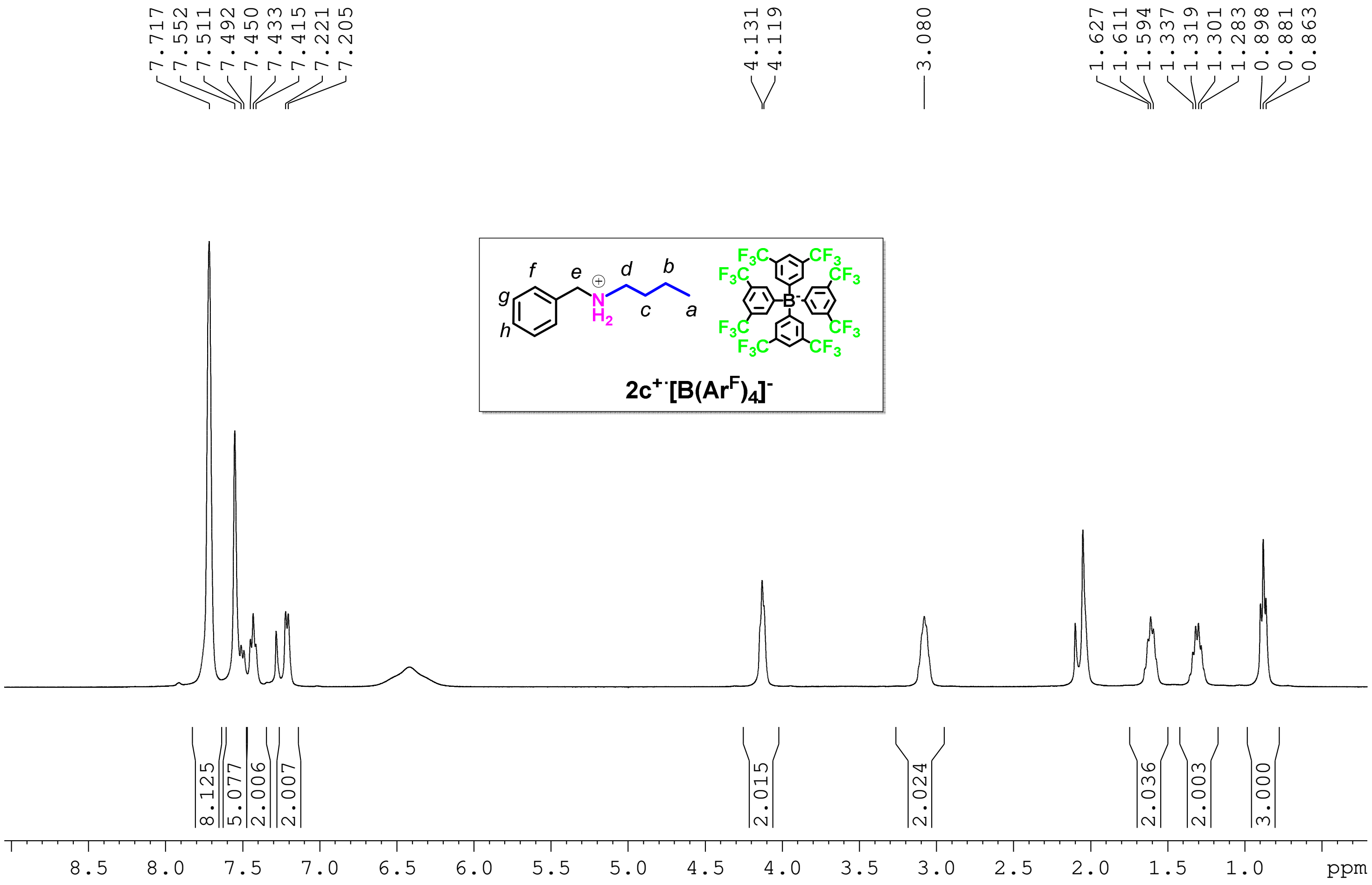

Figure S5. ^{1}H NMR spectrum of **2c^{+}·[B(Ar^{F})$_4$]$^{-}$** (400 MHz, $CDCl_3$, 298 K).

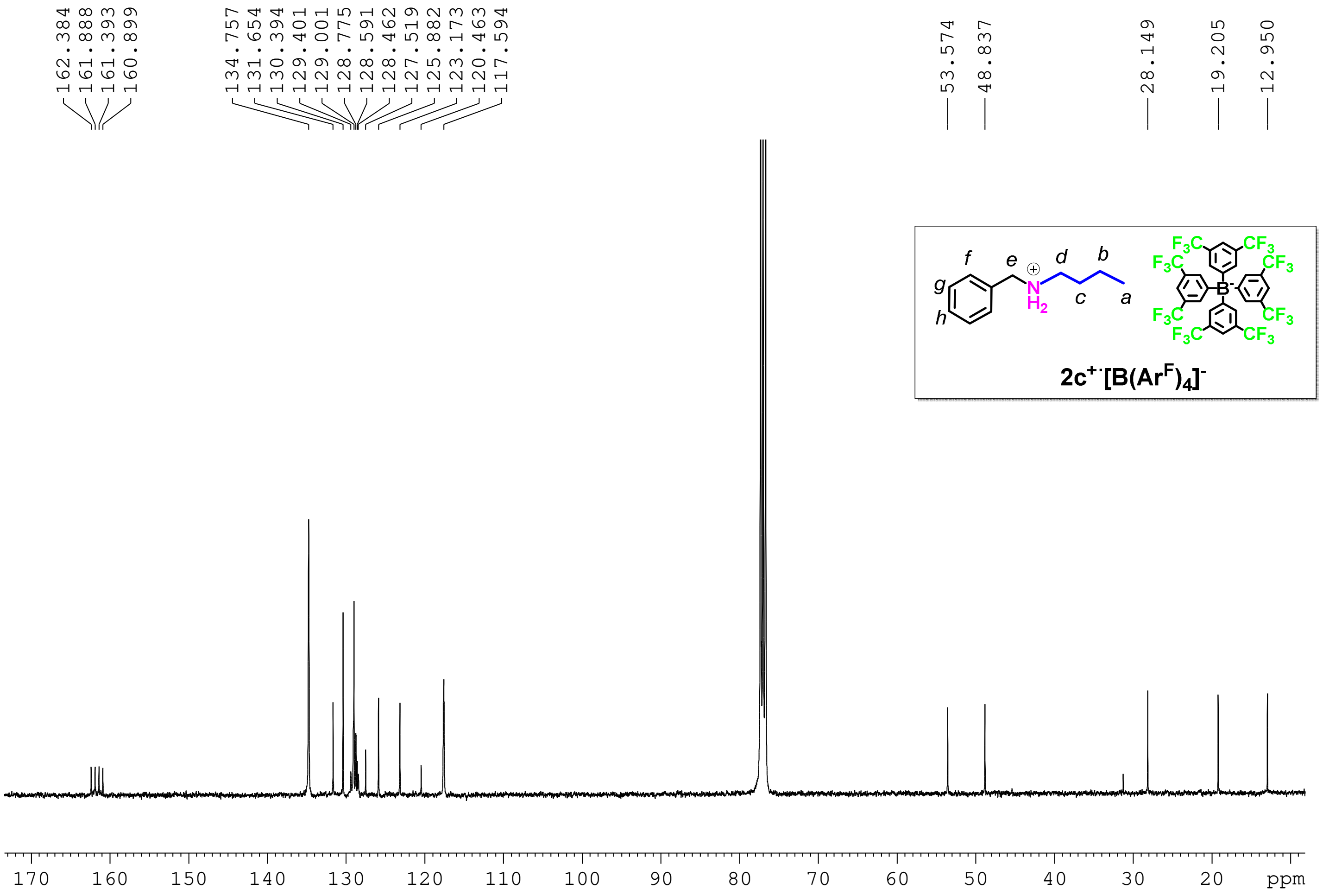

Figure S6. ^{13}C NMR spectrum of **$2c^{+}\cdot[B(Ar^{F})_4]^{-}$** (100 MHz, $CDCl_3$, 298 K).

^{1}H and ^{13}C NMR spectra of 2d^{+}·[B(Ar^{F})$_4$]$^{-}$

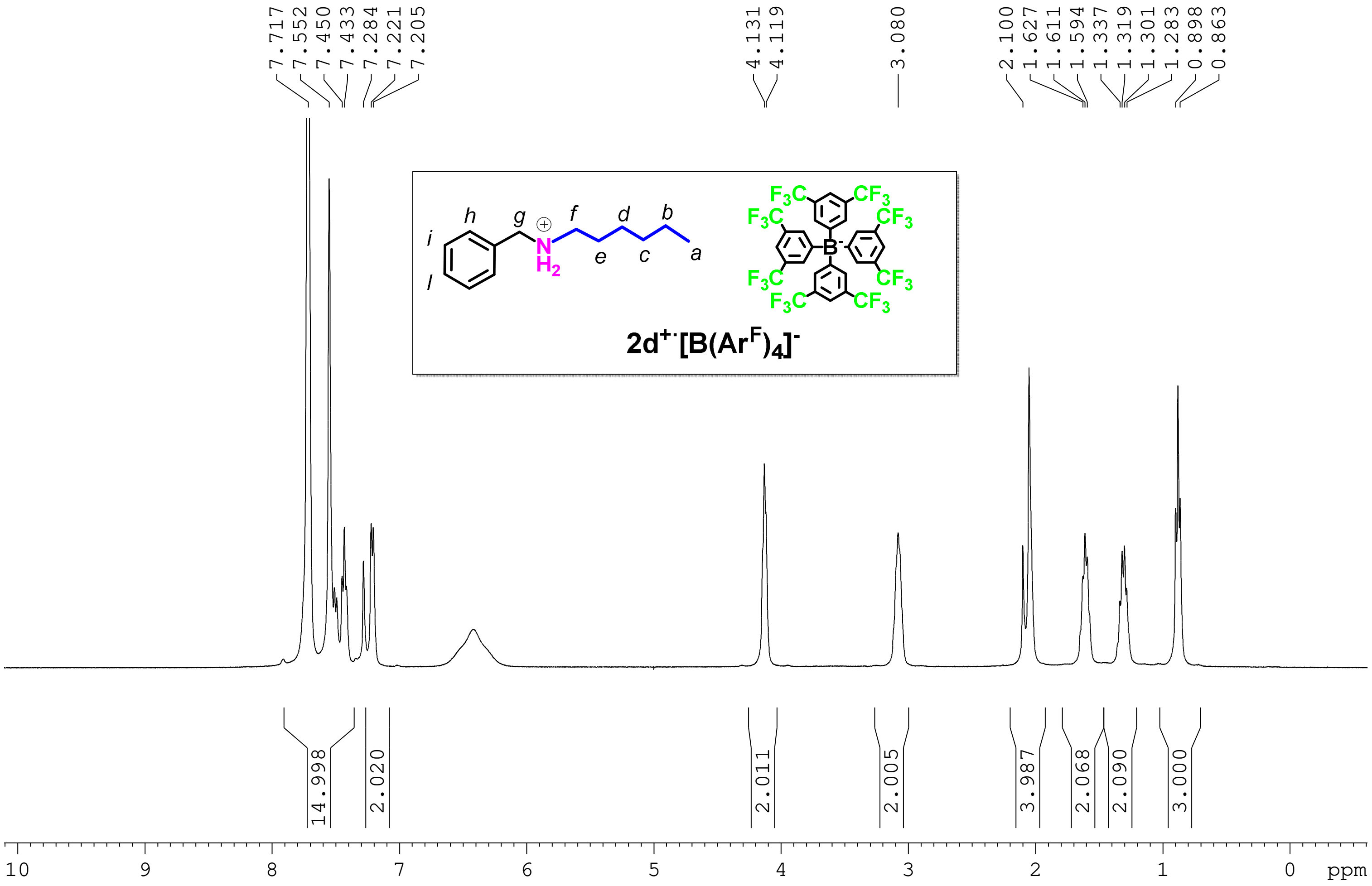

Figure S7. ^{1}H NMR spectrum of **2d^{+}·[B(Ar^{F})$_4$]$^{-}$** (100 MHz, $CDCl_3$, 298 K).

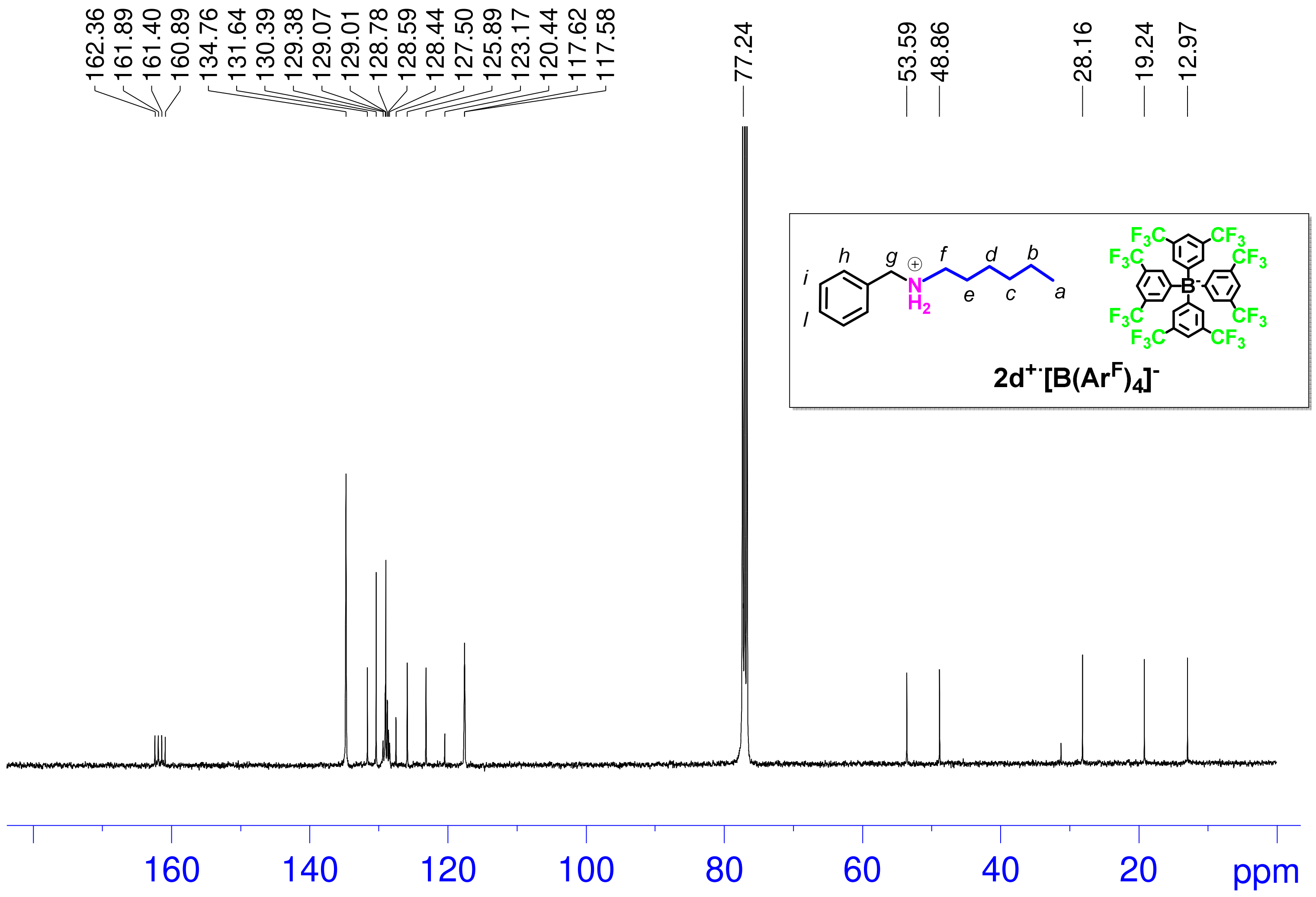

Figure S8. ^{13}C NMR spectrum of **2d^{+}·[B(Ar^{F})$_4$]$^{-}$** (100 MHz, $CDCl_3$, 298 K).

^{1}H and ^{13}C NMR spectra of 2e^{+}·[B(Ar^{F})$_4$]$^{-}$

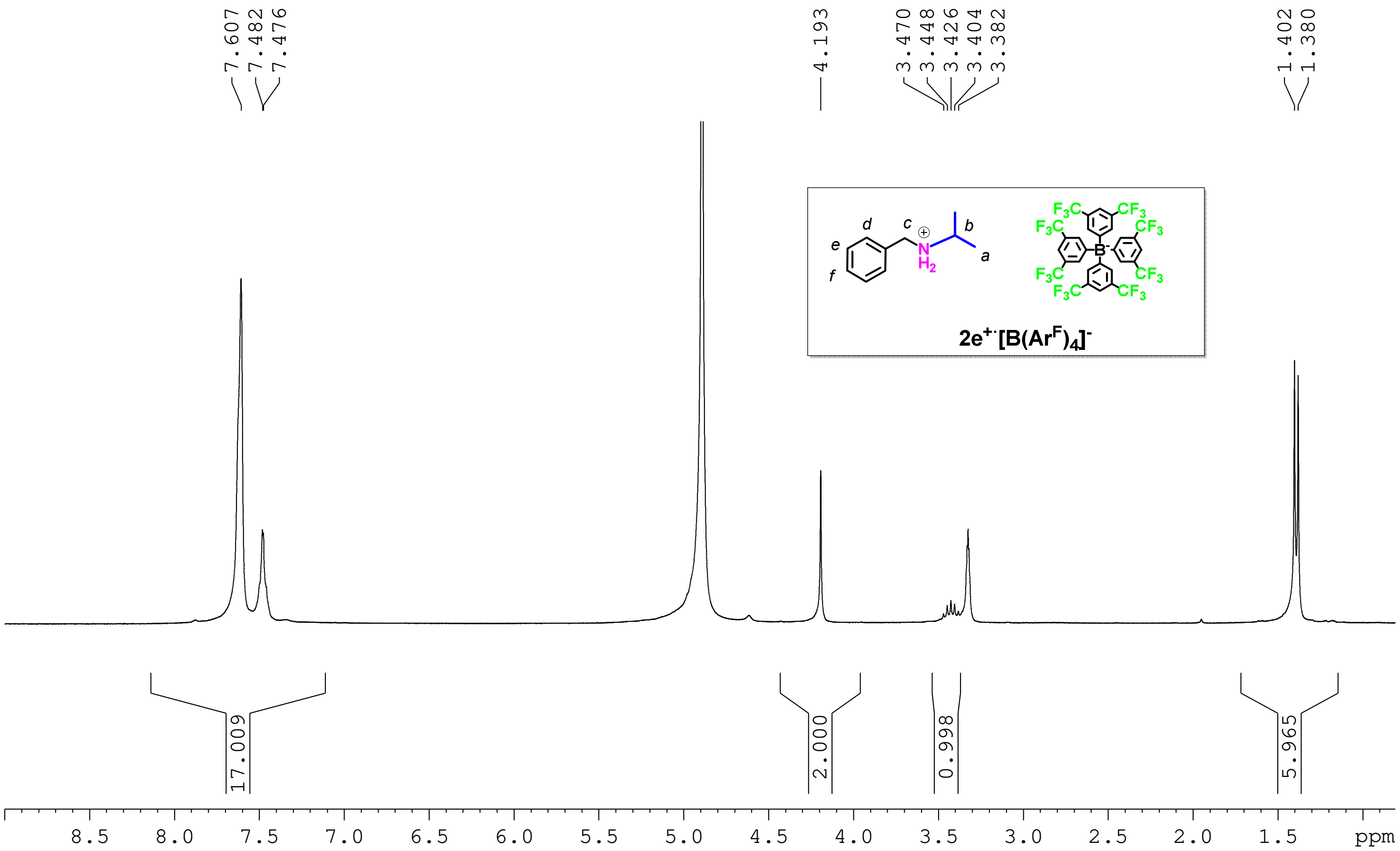

Figure S9. ^{1}H NMR spectrum of **2e^{+}·[B(Ar^{F})$_4$]$^{-}$** (300 MHz, CD_3OD, 298 K).

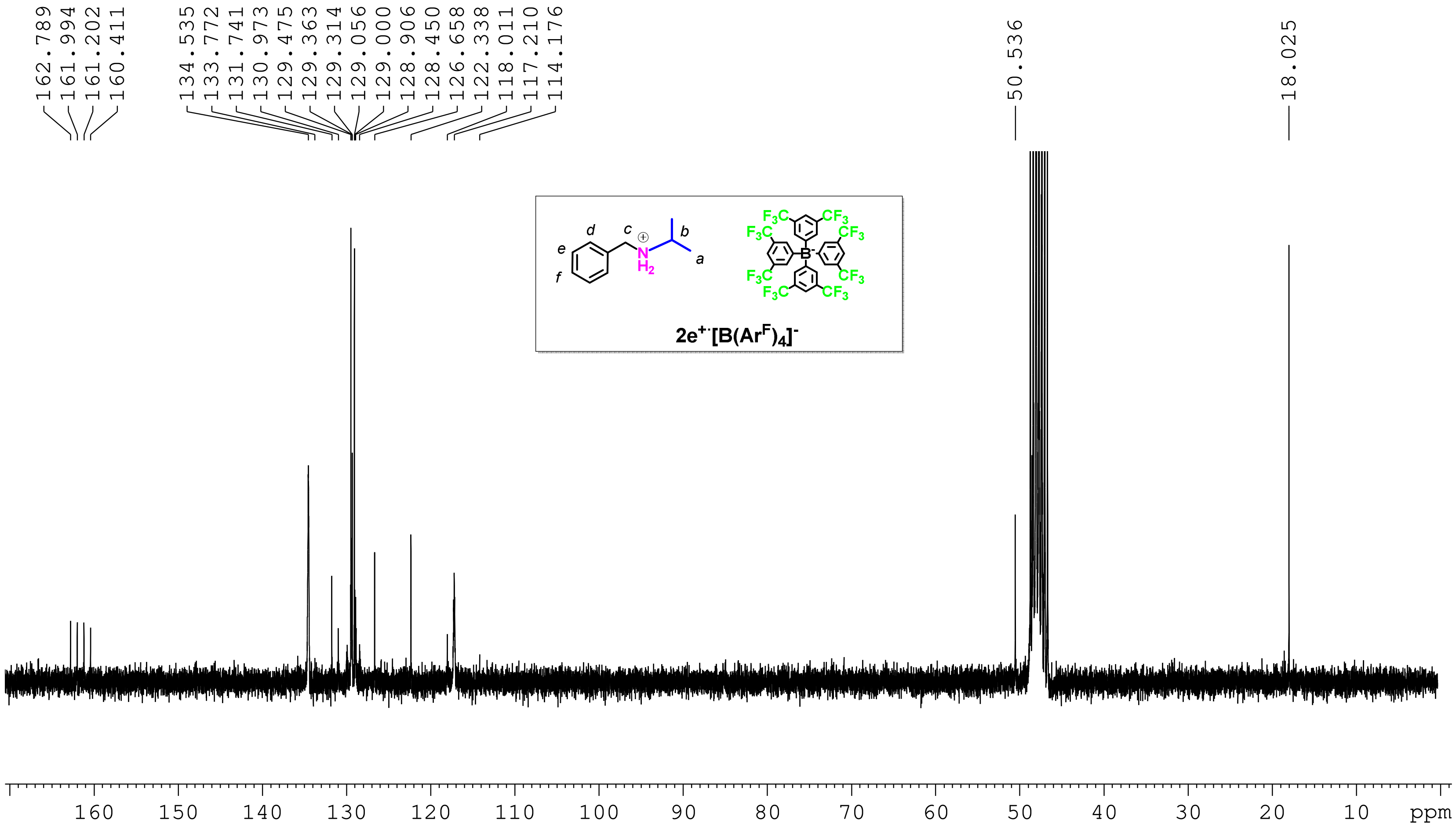

Figure S10. ^{13}C NMR spectrum of **2e^{+}·[B(Ar^{F})$_4$]$^{-}$** (65 MHz, CD$_3$OD, 298 K).

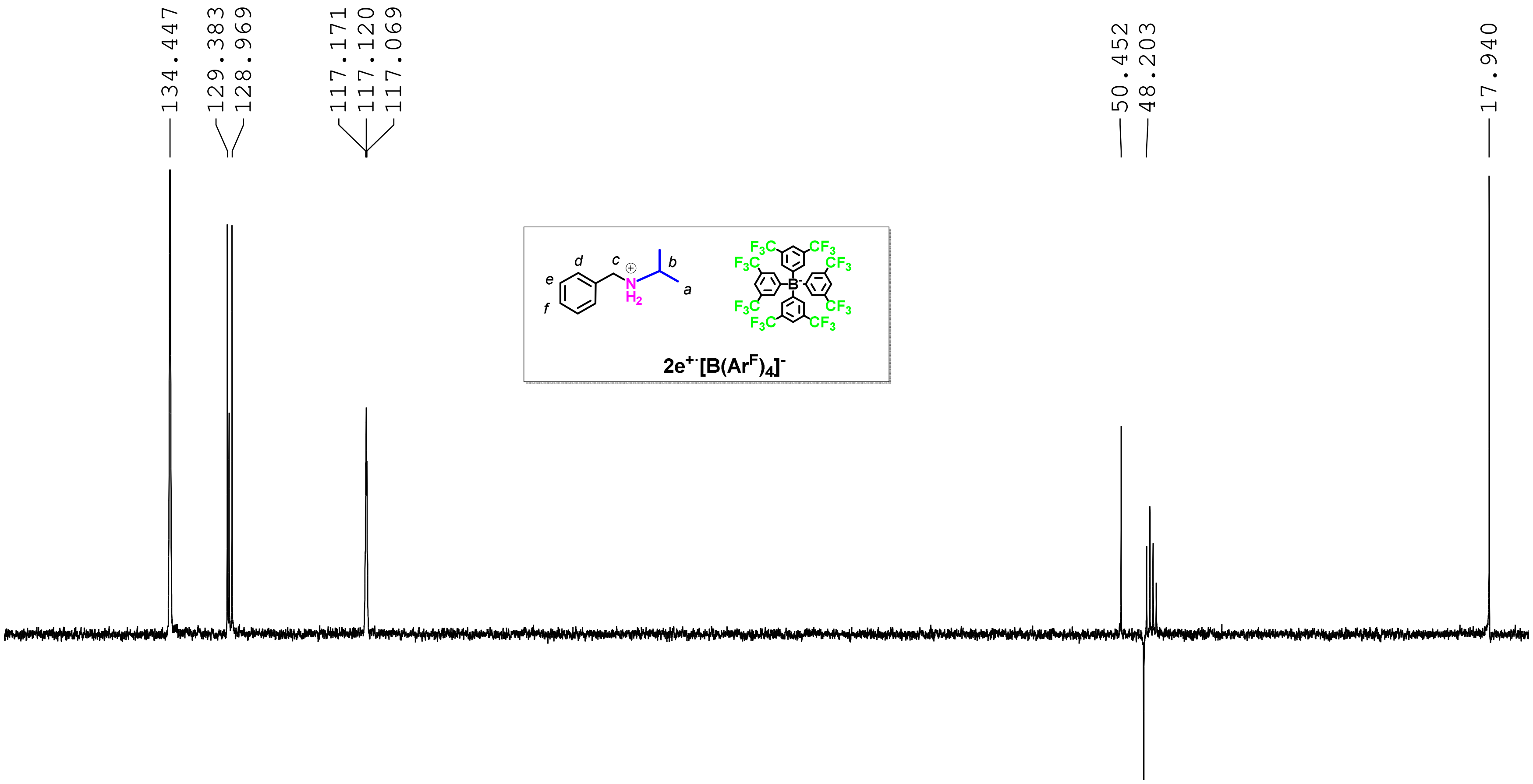

Figure S11. DEPT-135 NMR-spectrum of **2e⁺·[B(ArF)4]⁻** (75 MHz, CD_3OD, 298 K).

^{1}H and ^{13}C NMR spectra of 2f^{+}·[B(Ar^{F})$_4$]$^{-}$

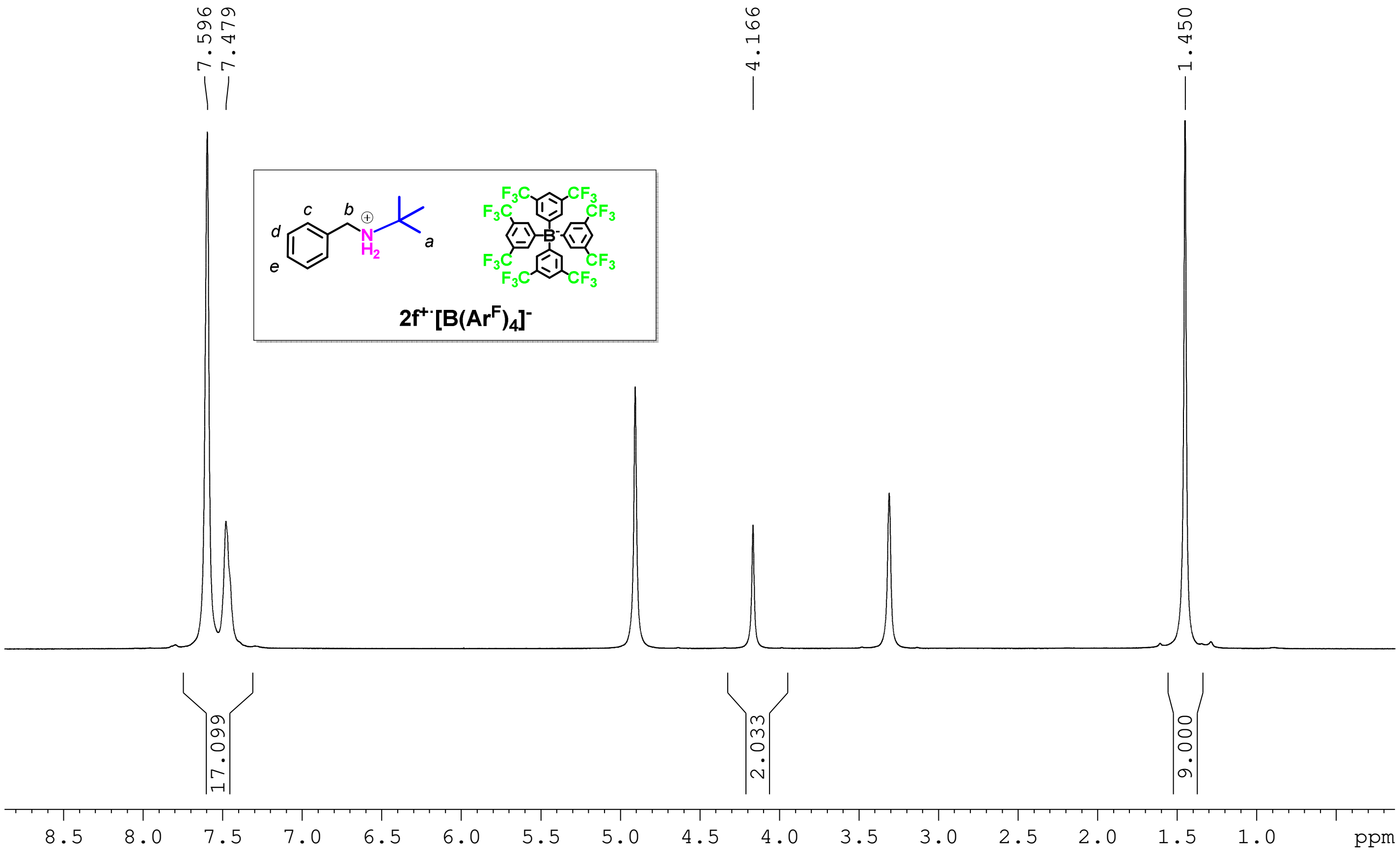

Figure S12. ^{1}H NMR spectrum of **2f^{+}·[B(Ar^{F})$_4$]$^{-}$** (400 MHz, CD_3OD, 298 K).

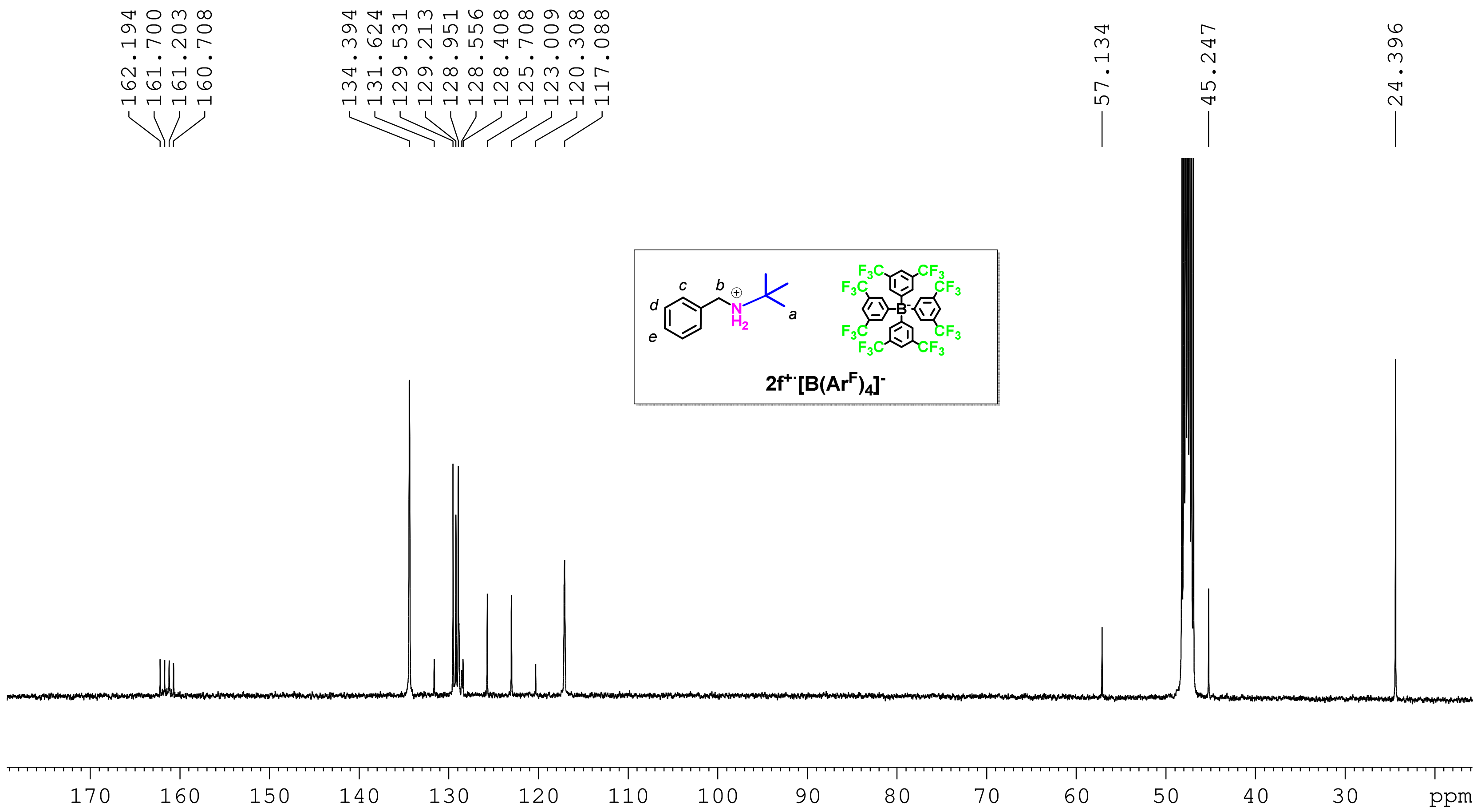

Figure S13. ^{13}C NMR spectrum of **2f^{+}·[B(Ar^{F})$_4$]$^{-}$** (100 MHz, CD_3OD, 298 K).

^{1}H and ^{13}C NMR spectra of 2g^{+}·[B(Ar^{F})$_4$]$^{-}$

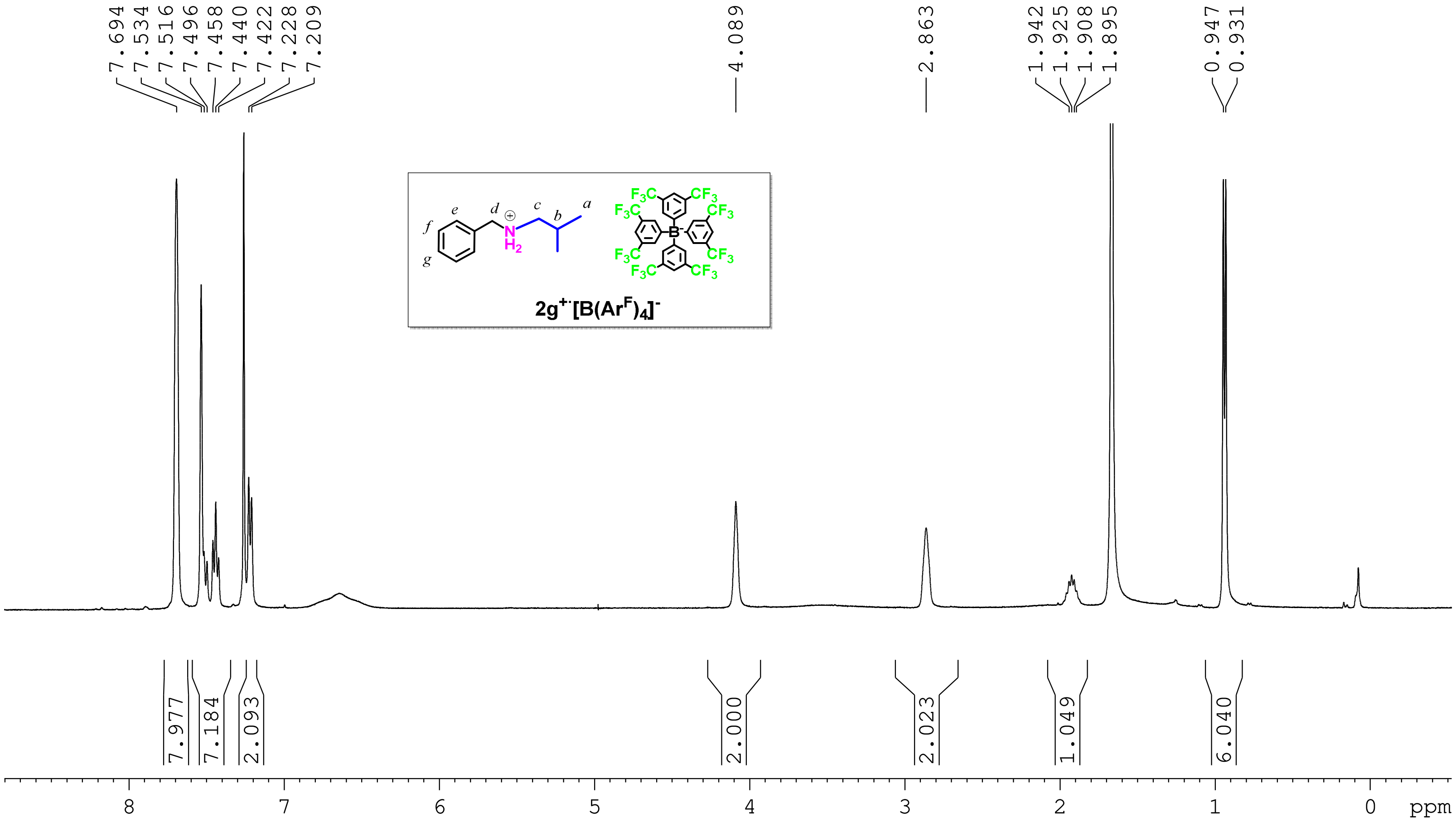

Figure S14. ^{1}H NMR spectrum of **2g^{+}·[B(Ar^{F})$_4$]$^{-}$** (400 MHz, $CDCl_3$, 298 K).

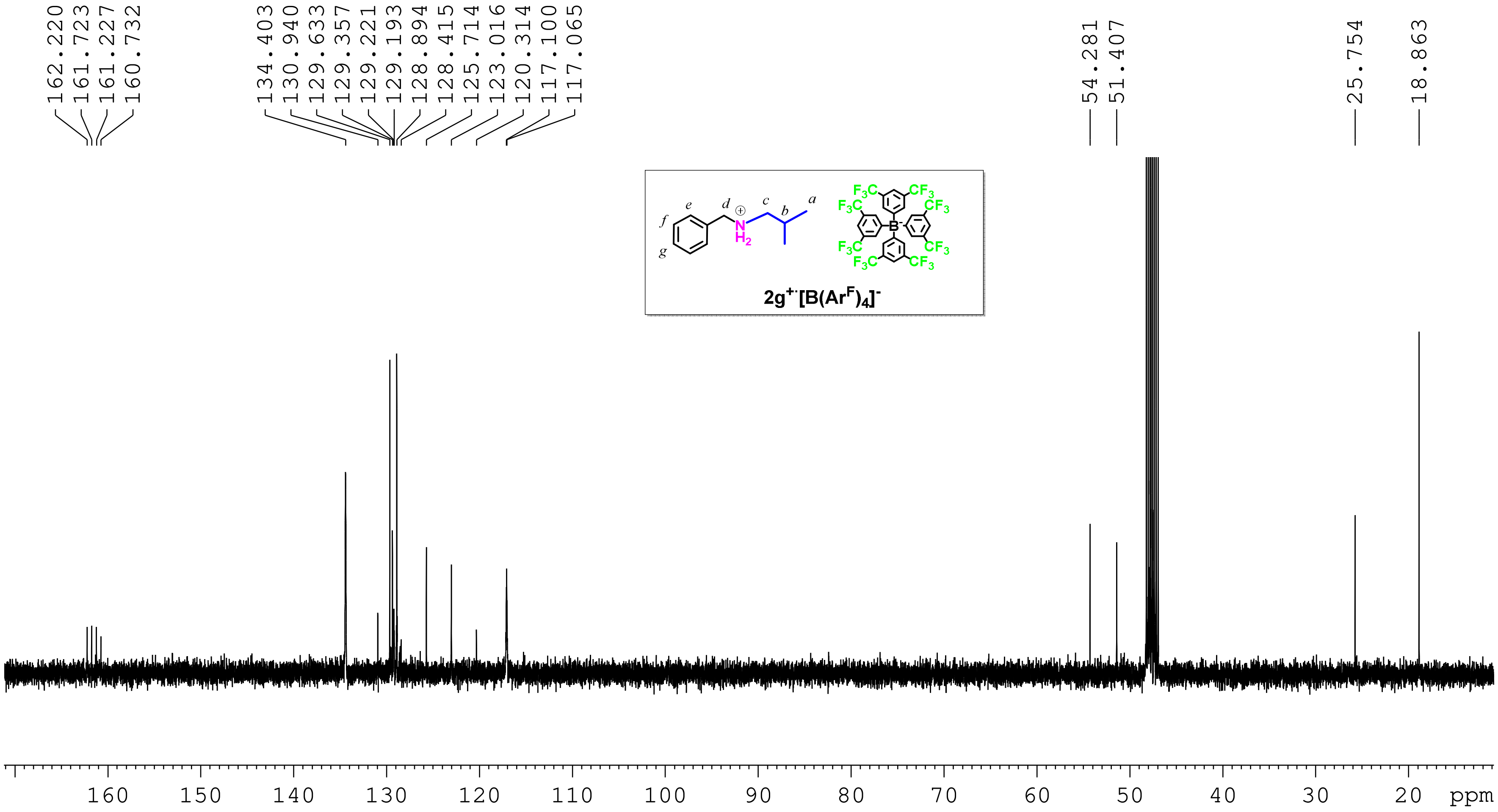

Figure S15. ^{13}C NMR spectrum of **$2g^{+}$·$[B(Ar^F)_4]^{-}$** (100 MHz, $CDCl_3$, 298 K).

^{1}H and ^{13}C NMR spectra of 2i^{+}·[B(Ar^{F})$_4$]$^{-}$

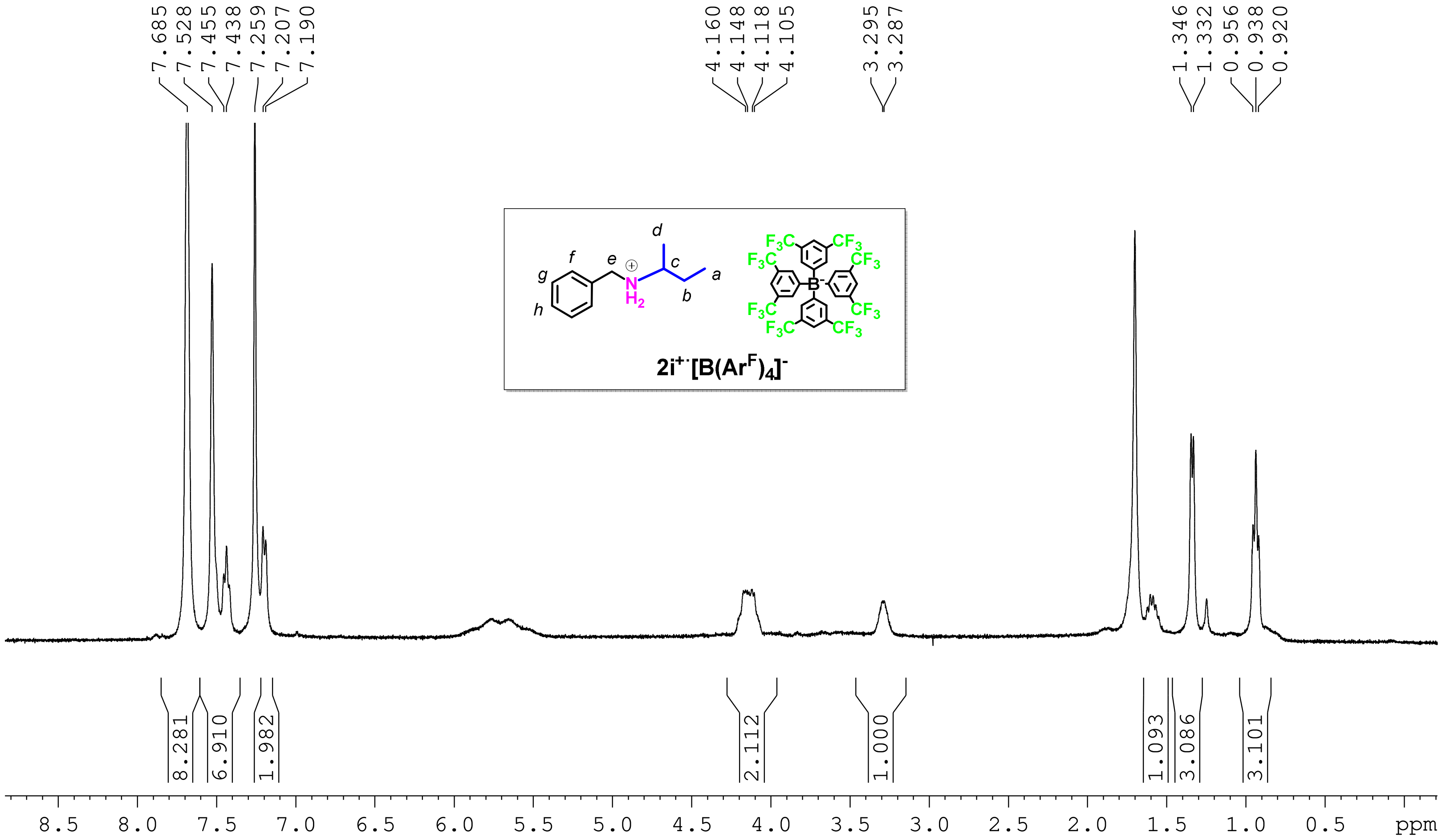

Figure S16. ^{1}H NMR spectrum of **2i^{+}·[B(Ar^{F})$_4$]$^{-}$** (400 MHz, $CDCl_3$, 298 K).

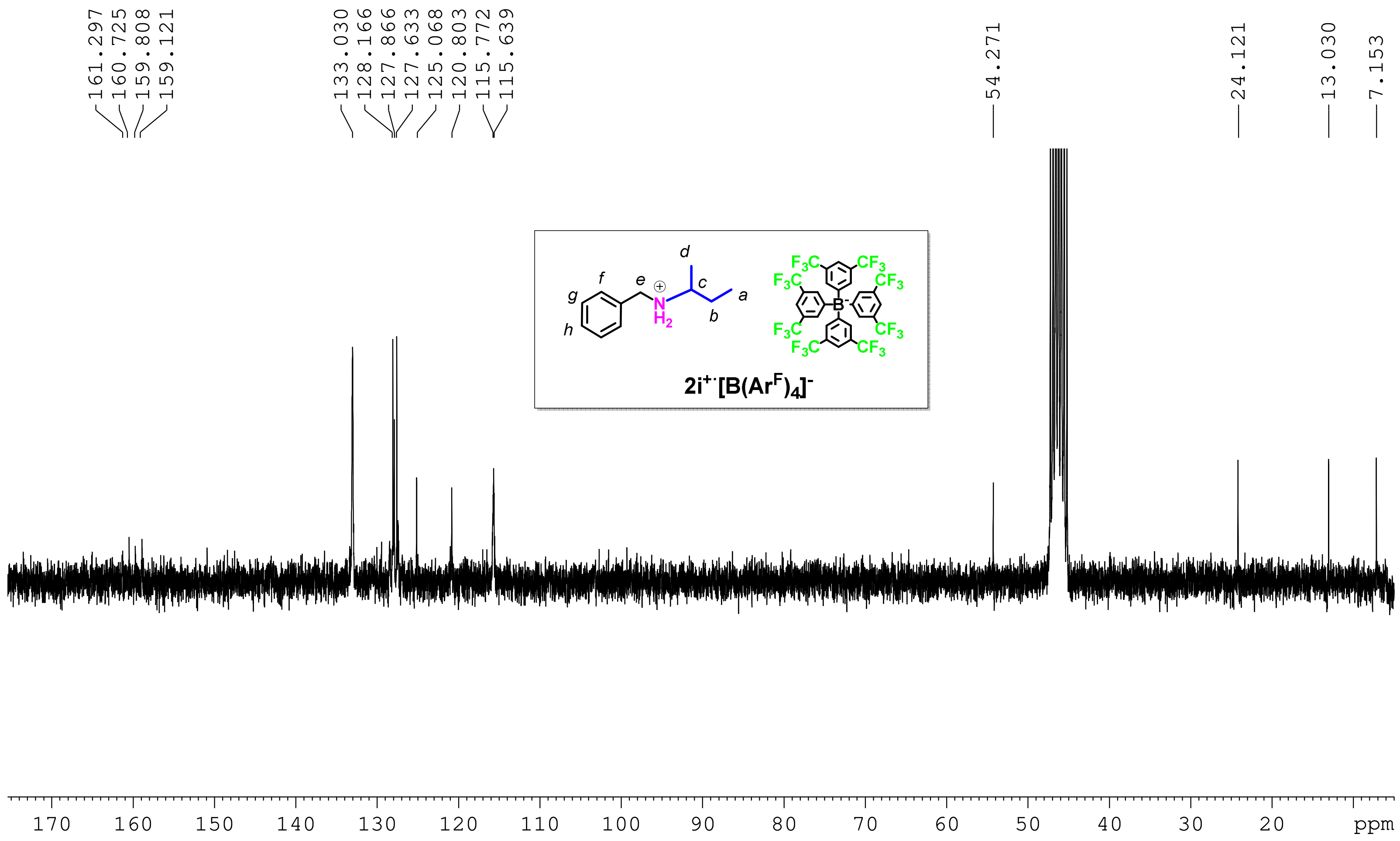

Figure S17. ^{13}C NMR spectrum of **2i^{+}·[B(Ar^{F})$_4$]$^{-}$** (65 MHz, CD_3OD, 298 K).

Synthesis of 2h$^+$·[B(Ar^F)$_4$]$^-$

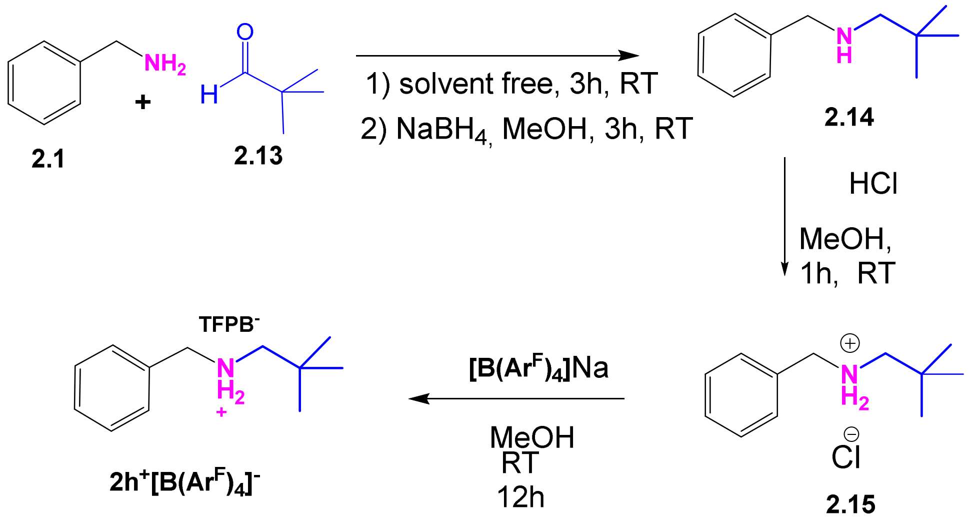

Scheme S4. Synthesis of derivative **2h$^+$·[B(Ar^F)$_4$]$^-$**

To benzylamine **2.1** (10.0 mmol) was added 3,3-dimethylbutyraldehyde **2.13** (10.0 mmol) and the reaction mixture was stirred at room temperature for 3 h. The resulting imine (10.0 mmol) was dissolved in dry MeOH (20 mL) under a nitrogen atmosphere and $NaBH_4$ (10.0 mmol) was added at 0 °C and then the mixture was allowed to warm at room temperature. The solution was kept under stirring for 3 h. The solvent was removed under reduced pressure and the residue partitioned between AcOEt (30 mL) and an aqueous saturated solution of $NaHCO_3$ (30 mL). The organic layer was dried over $MgSO_4$ and the solvent was removed under reduced pressure, to give derivative **2.14** as a yellow viscous liquid. The compound was used for the next step without further purification. The crude product (10.0 mmol) was dissolved in Et_2O (20 mL) at room temperature and an aqueous solution of HCl (37% w/w, 11.0 mmol) was added dropwise. The mixture was kept under stirring for 1 h, until the formation of a white precipitate. The solid was collected by filtration, purified by crystallization with hexane/MeOH and dried under vacuum, to give derivative **2.15** as a white solid. Derivative **2.15**$^-$ (0.15 mmol) was dissolved in dry MeOH (5.0 mL), then sodium tetrakis[3,5-bis(trifluoromethyl)phenyl]borate (0.16 mmol) was added and the mixture was kept under stirring overnight in the dark. The solvent was removed and deionized water was added, obtaining a brown precipitate that was filtered off and dried under vacuum to give **2h$^+$·[B(Ar^F)$_4$]$^-$**.

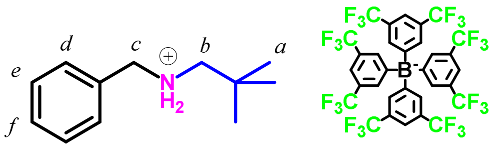

2h^{+}·[B(Ar^{F})$_4$]$^{-}$

2h^{+}·[B(Ar^{F})$_4$]$^{-}$: (yield: 0.09 g, 0.22 mmol, 95%, calculated with respect to derivative **2.15**). **ESI(+) MS**: m/z = 178.17 (M+). **^{1}H NMR** (300 MHz, $CDCl_3$, 298 K): δ 7.22-7.71 (overlapped, 5H, $H_{d\text{-}f}$+ 12H, Ar$H^{[B(ArF)_4]^-}$), 4.17 (s, 2H, H_c), 2.86 (s 2H, H_b), 0.94 (s, 9H, H_a); **^{13}C NMR** (75 MHz, $CDCl_3$, 298 K): δ 162.8, 162.1, 161.4, 160.8, 134.9, 131.6, 130.4, 130.1, 129.6, 129.2, 128.8, 128.8, 128.4, 127.8, 126.5, 122.9, 119.3, 117.7, 117.6, 77.4, 60.2, 54.4, 31.4, 30.5, 26.5. Anal. Calcd for $C_{44}H_{32}BF_{24}N$: C 50.74, H 3.10, N 1.34. Found: C 50.73, H 3.11, N 1.35.

^{1}H and ^{13}C NMR spectra of 2h^{+}·[B(Ar^{F})$_4$]$^{-}$

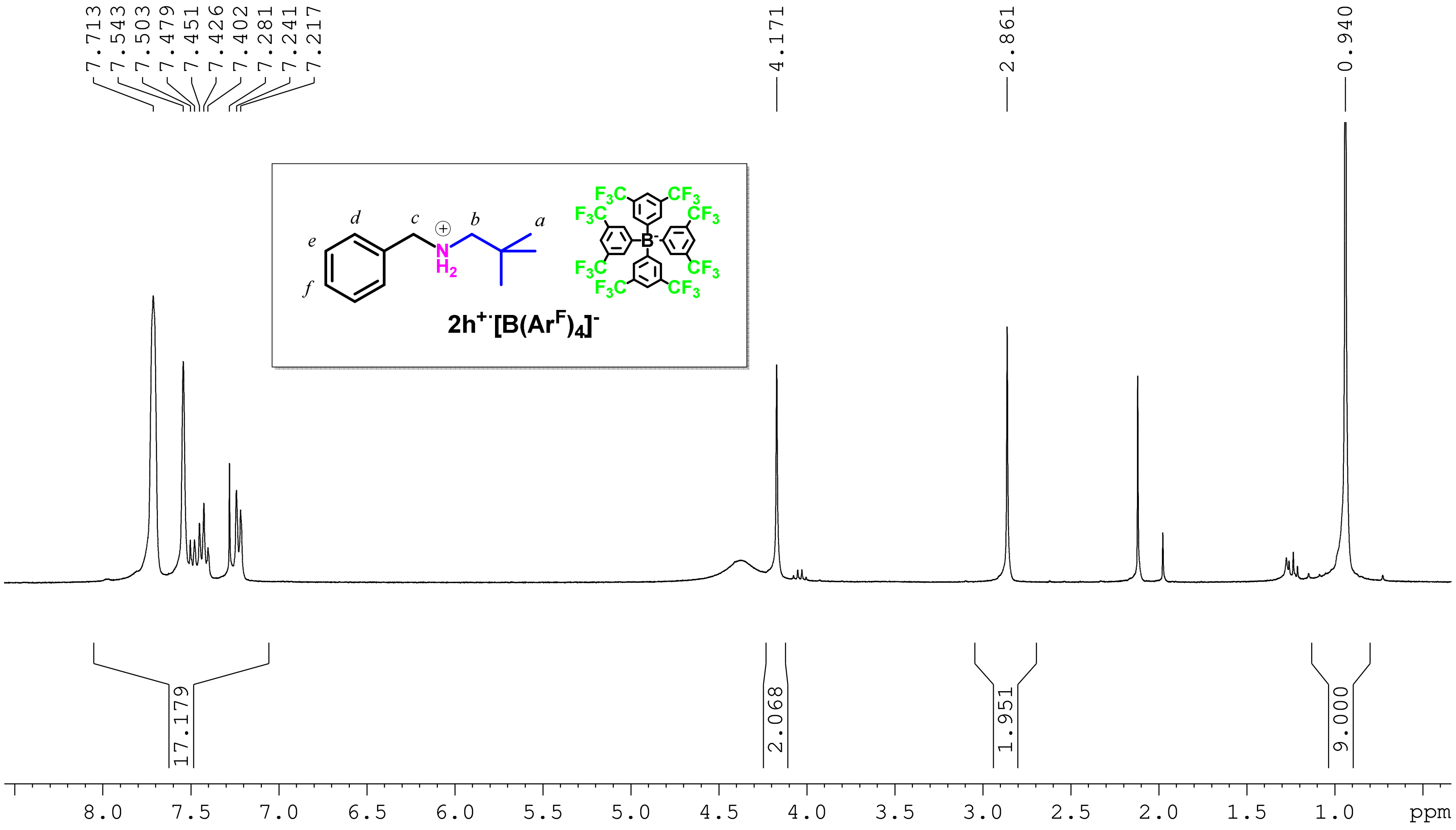

Figure S18. ^{1}H NMR spectrum of **2h^{+}·[B(Ar^{F})$_4$]$^{-}$** (300 MHz, $CDCl_3$, 298 K).

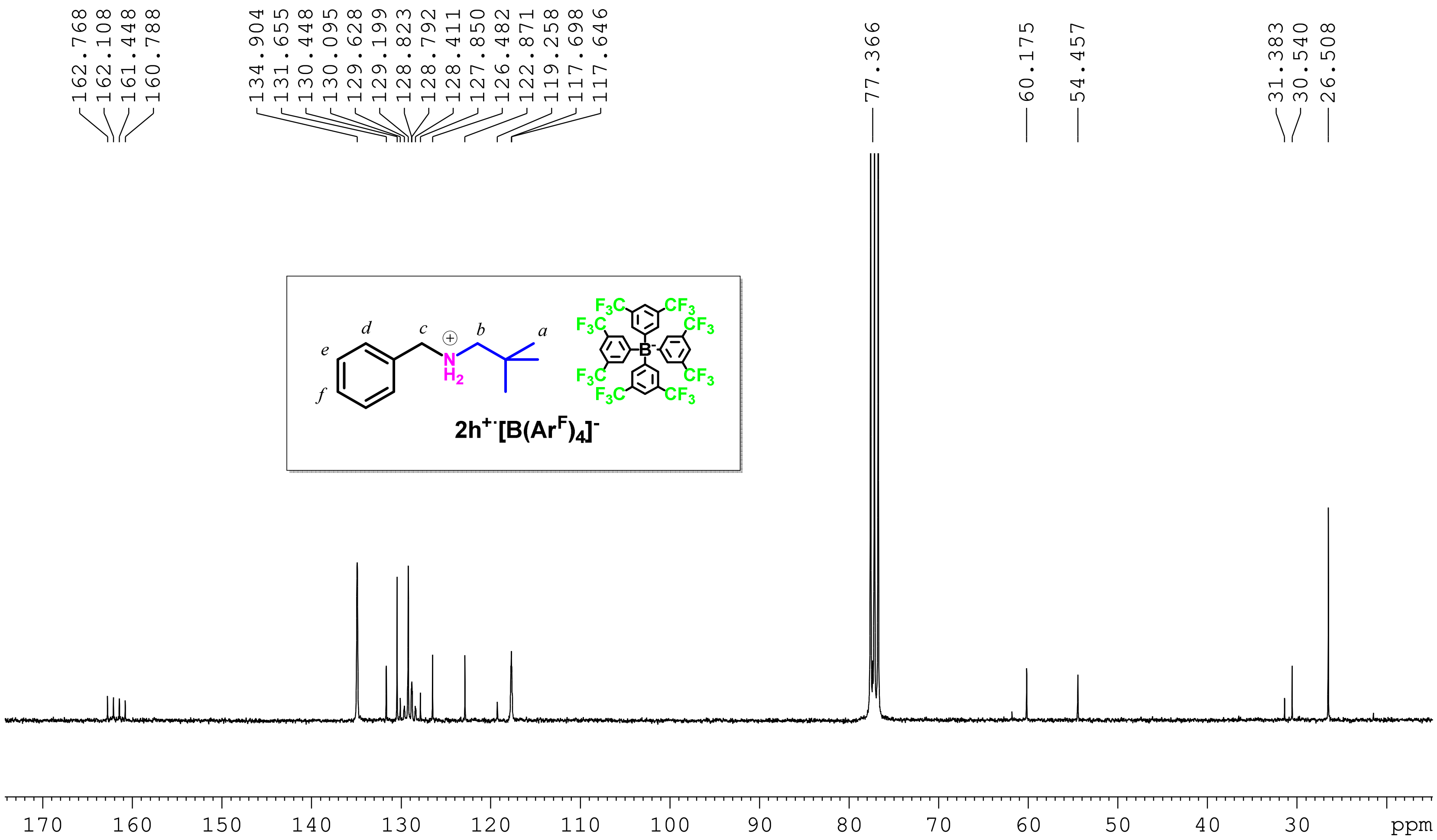

Figure S19. ^{13}C NMR spectrum of **$2h^{+}$·[B(Ar^{F})$_4$]$^{-}$** (75 MHz, $CDCl_3$, 298 K).

Synthesis of 2j,k^{+}·[B(Ar^{F})$_4$]$^-$

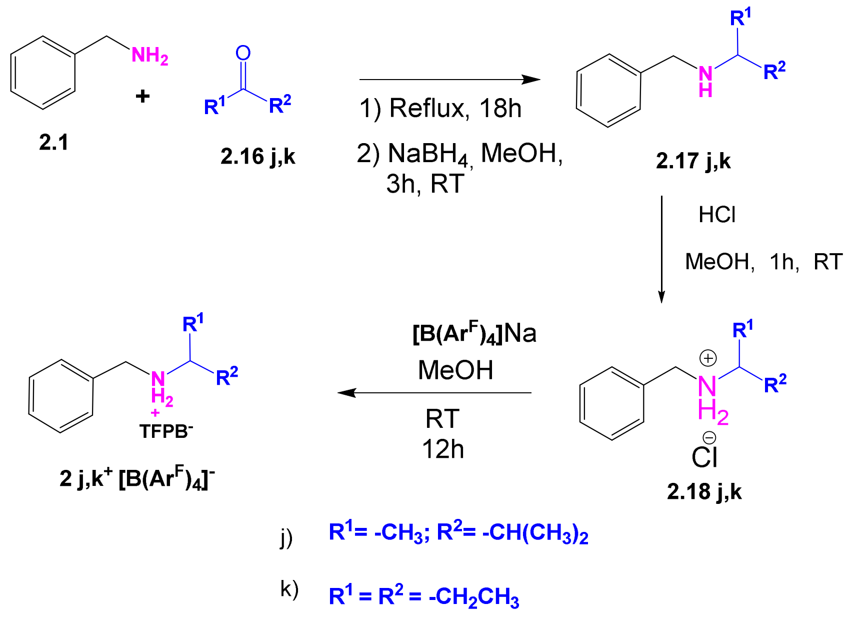

Scheme S4. Synthesis of **2j,k^{+}·[B(Ar^{F})$_4$]$^-$**

Benzylamine **2.1** (10.0 mmol) was dissolved into the corresponding ketone **2.16** (40 mL) and the reaction mixture was stirred at reflux for 18 h. The reaction mixture was then cooled to room temperature and the excess of ketone was removed under reduced pressure. The resulting imine (10.0 mmol) was dissolved in dry MeOH (20 mL) under a nitrogen atmosphere and $NaBH_4$ (10.0 mmol) was added at 0 °C and then the mixture was allowed to warm at room temperature. The solution was kept under stirring for 3 h. The solvent was removed under reduced pressure and the residue partitioned between AcOEt (30 mL) and an aqueous saturated solution of $NaHCO_3$ (30 mL). The organic layer was dried over $MgSO_4$ and the solvent was removed under reduced pressure, to give derivative **2.17 j-k** as a yellow viscous liquid. The compound was used for the next step without further purification. The crude product (10.0 mmol) was dissolved in Et_2O (20 mL) at room temperature and an aqueous solution of HCl (37% w/w, 11.0 mmol) was added dropwise. The mixture was kept under stirring for 1 h, until the formation of a white precipitate. The solid was collected by filtration, purified by crystallization with Exane/MeOH and dried under vacuum, to give derivative **2.18j-k** as a white solid. Derivative **2.18j-k** (1.0 eq.) was dissolved in dry MeOH (C= 0.20 M), then

sodium tetrakis[3,5-bis(trifluoromethyl)phenyl]borate (1.1 eq) was added and the mixture was kept under stirring overnight in the dark. The solvent was removed and deionized water was added, obtaining a brown precipitate that was filtered off and dried under vacuum to give derivatives **2j-k$^+$·TFPB$^-$**.

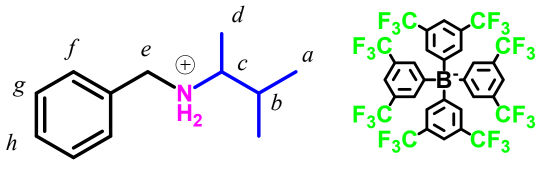

2j$^+$·[B(Ar^F)$_4$]$^-$: (yield: 0.09 g, 0.22 mmol, 95%, calculated with respect to derivative **2.18j**). **ESI(+) MS**: m/z = 178.17 (M+). **^{1}H NMR** (400 MHz, $CDCl_3$, 298 K): □ 7.71 (s, 8H, ArH$^{[B(ArF)_4]-}$), 7.55 (s, 4H, ArH$^{[B(ArF)_4]-}$), 7.23-7.52 (overlapped, 5H, $H_{f\text{-}g}$), 4.14 (m, broad 1H, H_e), 3.17 (m, broad 1H, H_c), 1.97 (m broad, 1H, H_b), 1.29 (d, J = 7, 3H, H_d), 0.86-1.00 (overlapped, 6H, H_{a+b}); **^{13}C NMR** (65 MHz, $CDCl_3$, 298 K): □ 163.9, 163.2, 162.5, 161.9, 135.8, 132.5, 131.0, 130.7, 130.3, 129.8, 127.6, 124.0, 120.4, 118.5, 60.4, 50.2, 30.8, 19.6, 15.7, 11.4. Anal. Calcd for $C_{44}H_{32}BF_{24}N$: C 50.74, H 3.10, N 1.34. Found: C 50.75, H 3.11, N 1.35.

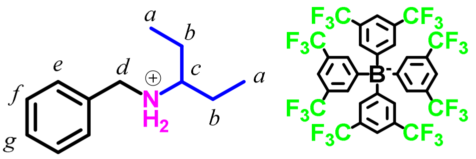

2k$^+$·[B(Ar^F)$_4$]$^-$: (yield: 0.12 g, 0.11 mmol, 78%, calculated with respect to derivative **2.18k**). **ESI(+) MS**: m/z = 178,15 (M$^+$). **^{1}H NMR** (400 MHz, $CDCl_3$, 298 K): □ 7.71 (s, 8H, ArH$^{[B(ArF)_4]-}$), 7.55 (s, 4H, ArH$^{[B(ArF)_4]-}$), 7.23-7.51 (overlapped, 5H, $H_{e\text{-}g}$), 4.17 (broad, 2H, H_d), 3.19 (m, broad, 1H, H_c), 1.74 (m, broad, 4H, H_b), 0.96 (t, J =7, 6H, H_a); **^{13}C NMR** (100 MHz, $CDCl_3$, 298 K): □ 162.7, 162.4, 162.0, 161.5, 161.0, 134.8, 132.5, 131.6, 130.6, 129.1, 128.9, 126.0, 123.3, 120.5, 117.7, 50.9, 22.8, 8.6. Anal. Calcd for $C_{44}H_{32}BF_{24}N$: C 50.74, H 3.10, N 1.34. Found: C 50.74, H 3.09, N 1.33.

^{1}H and ^{13}C NMR spectra of $2j^{+}\cdot[B(Ar^{F})_4]^{-}$

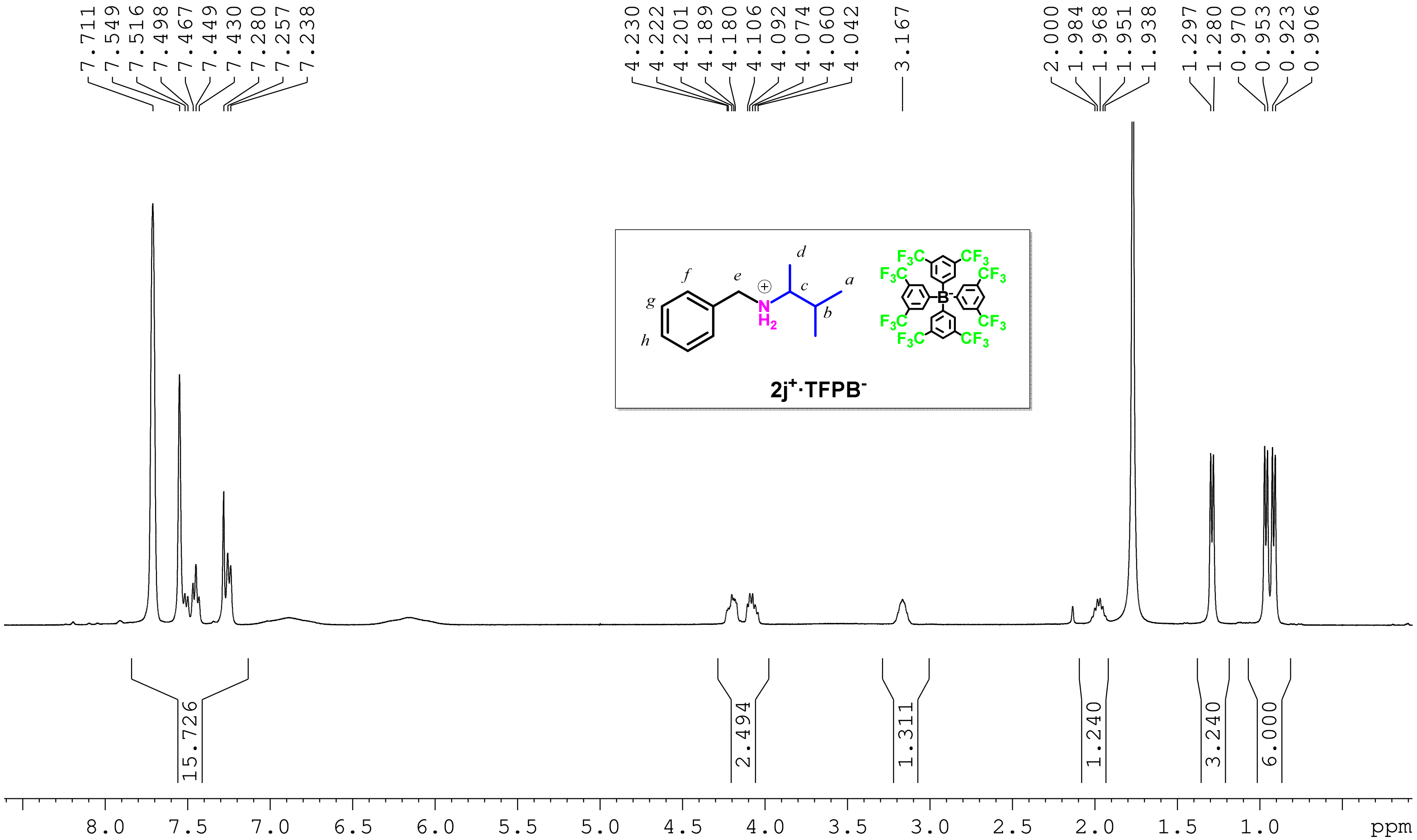

Figure S20. ^{1}H NMR spectrum of **$2j^{+}\cdot[B(Ar^{F})_4]^{-}$** (400 MHz, $CDCl_3$, 298 K).

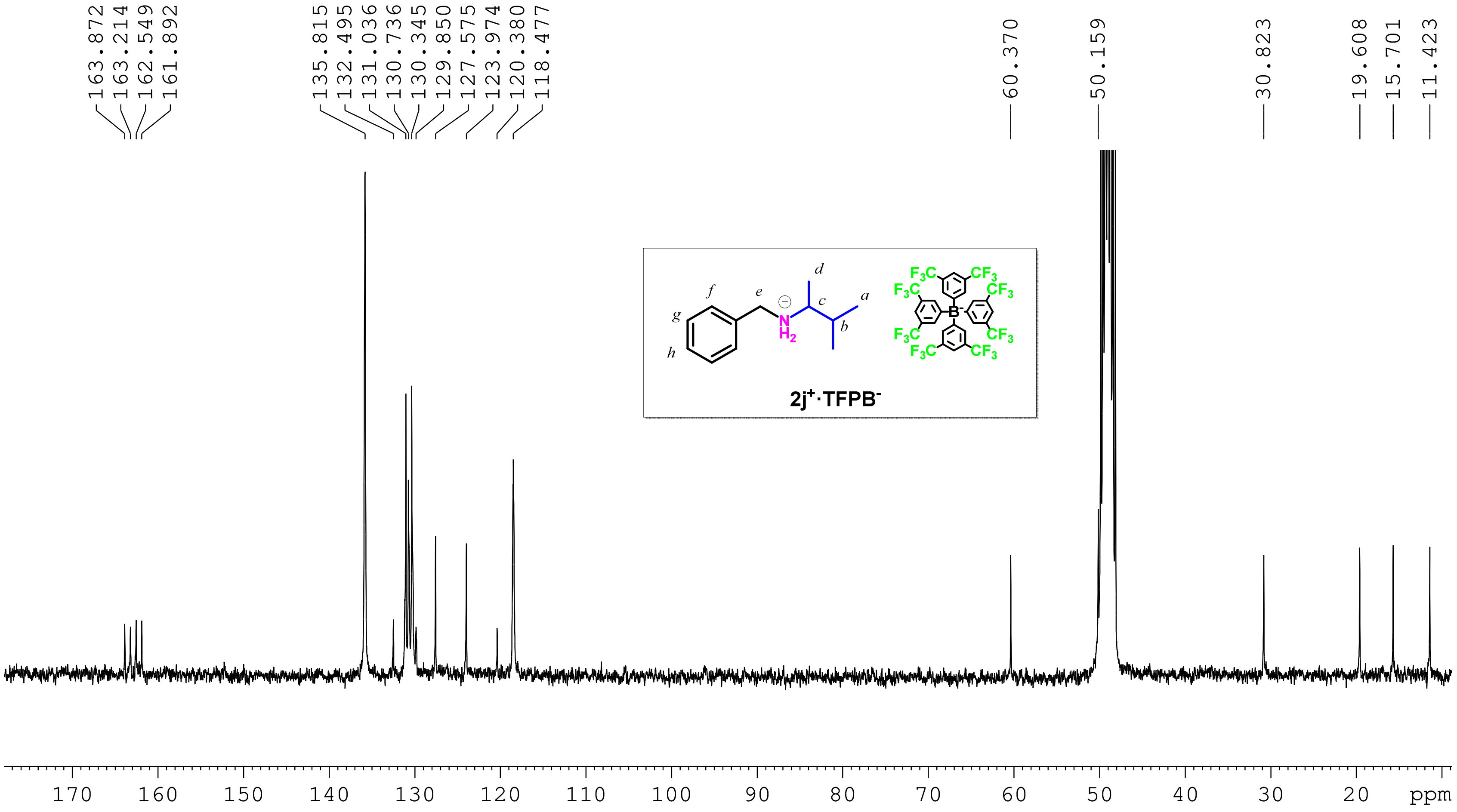

Figure S21. ${}^{13}C$ NMR spectrum of **2j⁺·[B(Ar^F)_4]⁻** (100 MHz, $CDCl_3$, 298 K).

^{1}H and ^{13}C NMR spectra of 2k^{+}·[B(Ar^{F})$_4$]$^{-}$

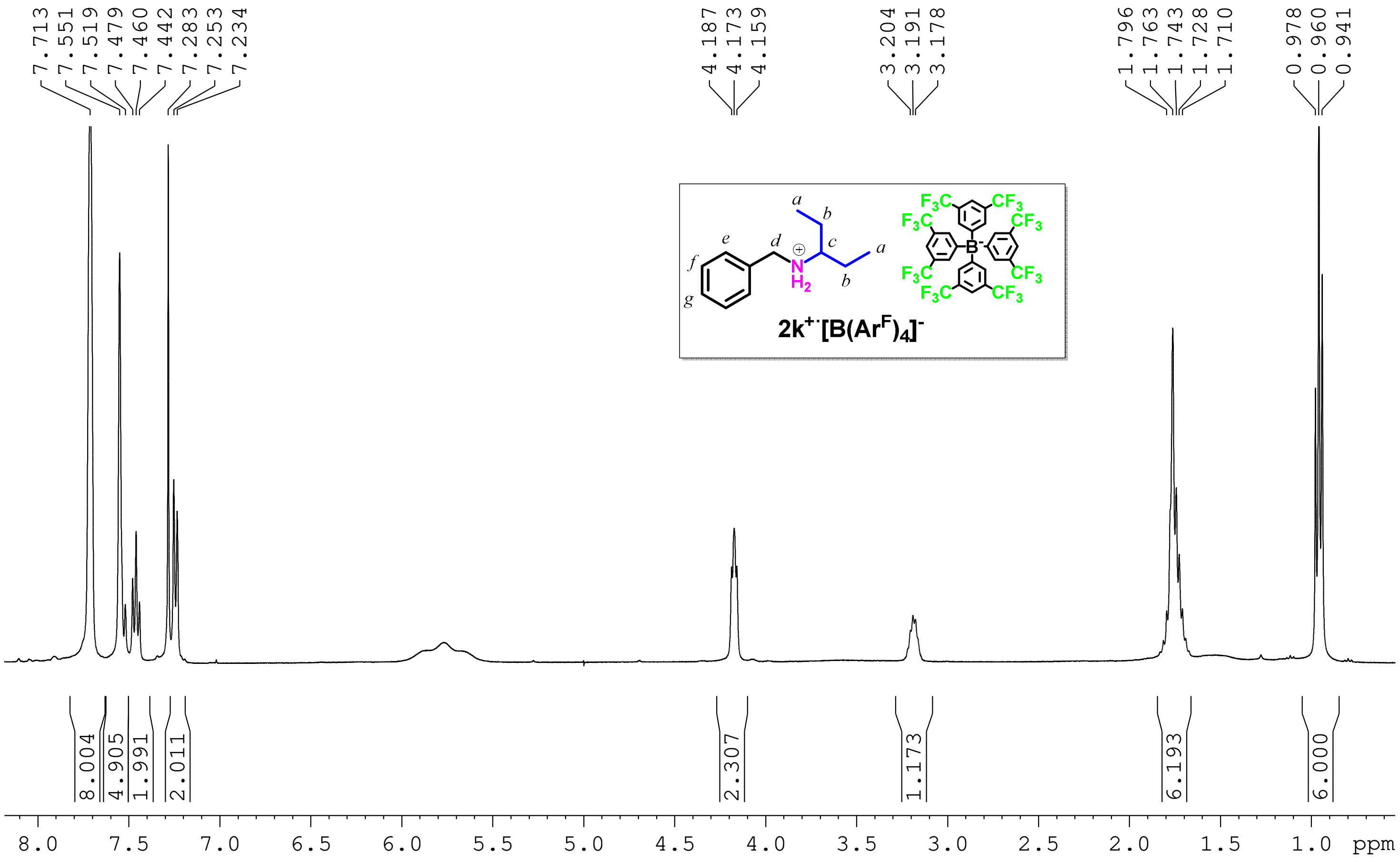

Figure S22. ^{1}H NMR spectrum of **2k^{+}·[B(Ar^{F})$_4$]$^{-}$** (400 MHz, $CDCl_3$, 298 K).

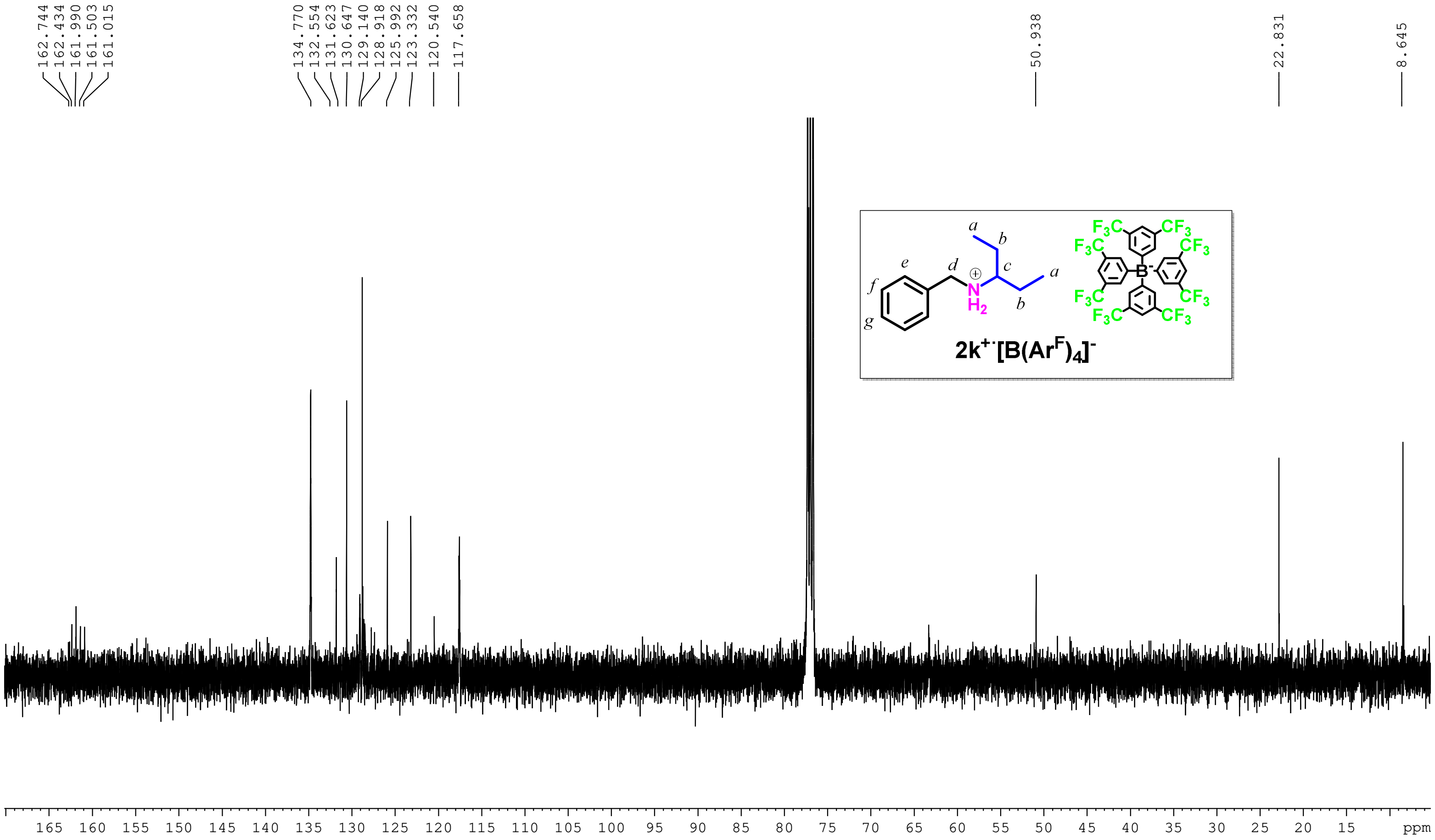

Figure S23. ^{13}C NMR spectrum of **2k^{+}·[B(Ar^{F})$_4$]$^{-}$** (100 MHz, $CDCl_3$, 298 K).

General procedure for the preparation of Pseudo[2]rotaxanes 2a–k$^+$⊂1

Calixarene derivative **1** ($1.9\cdot10^{-3}$ mmol) was dissolved in 0.5 mL of $CDCl_3$ ($3.8\cdot10^{-3}$ M solution). Then, the appropriate **$[B(Ar^F)_4]^-$** salt (**$2a$-$k^+\cdot[B(Ar^F)_4]^-$**) was added ($1.9\cdot10^{-3}$ mmol, $3.8\cdot10^{-3}$ M) and the mixture was stirred for 15 min. Then, the solution was transferred in a NMR tube for 1D NMR spectra acquisition.

Scale-up of procedure for the preparation of Pseudo[2]rotaxane 2a$^+$⊂1

Calixarene derivative **1** (1.0 mmol) was dissolved in 26.3 mL of $CDCl_3$ ($3.8\cdot10^{-3}$ M solution). Then, **$2a^+\cdot[B(Ar^F)_4]^-$** salt (1.0 mmol) was added (final concentration $3.8\cdot10^{-3}$ M) and the mixture was stirred for 15 min. Then, 0.5 mL of the solution was transferred in a NMR tube for 1D and 2D NMR spectrum acquisition.

^{1}H NMR Spectrum of 2a$^+$⊂1

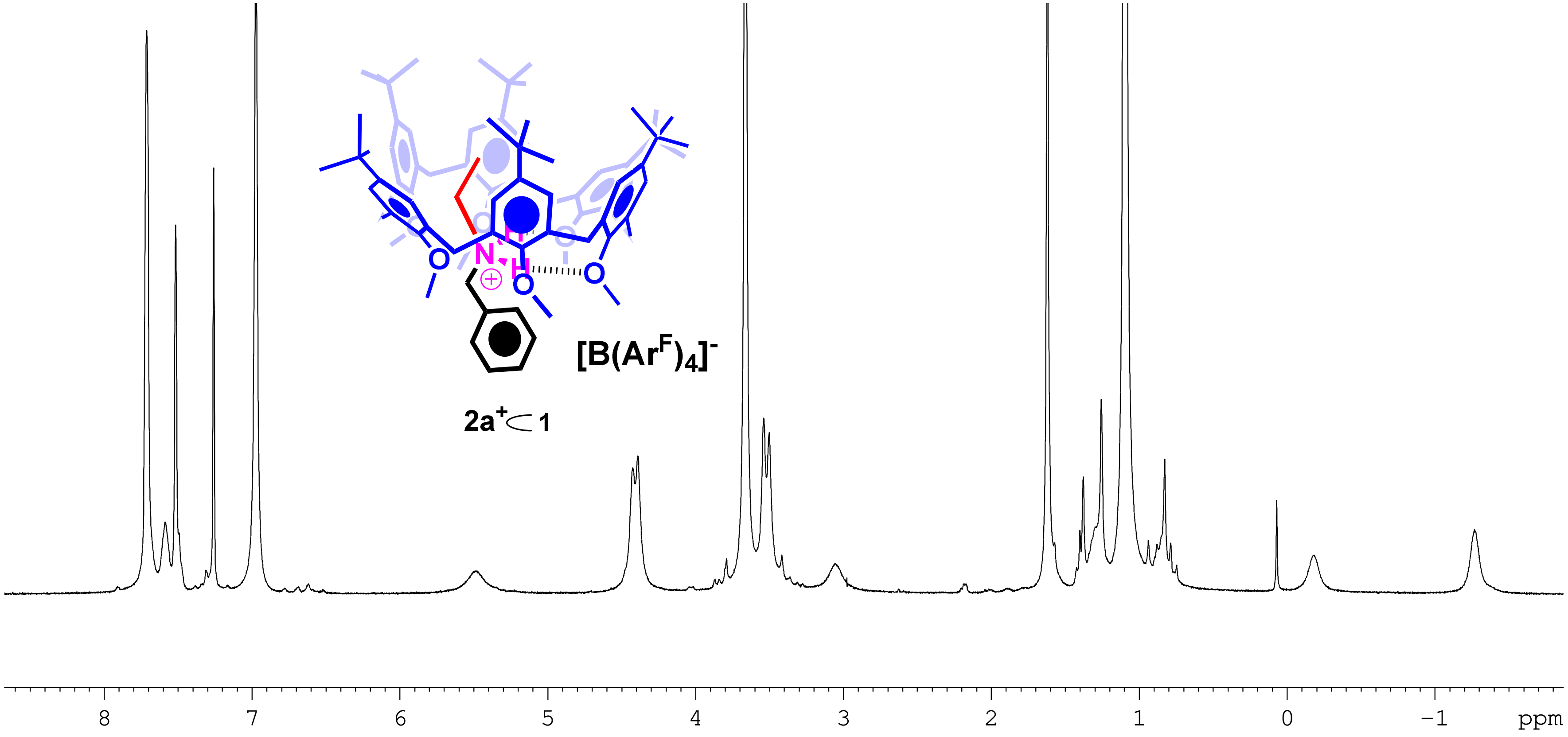

Figure S24. ^{1}H NMR spectrum of **2a$^+$⊂1** (400 MHz, $CDCl_3$, 298 K).

^{1}H NMR Spectrum of 2b$^+$⊂ 1

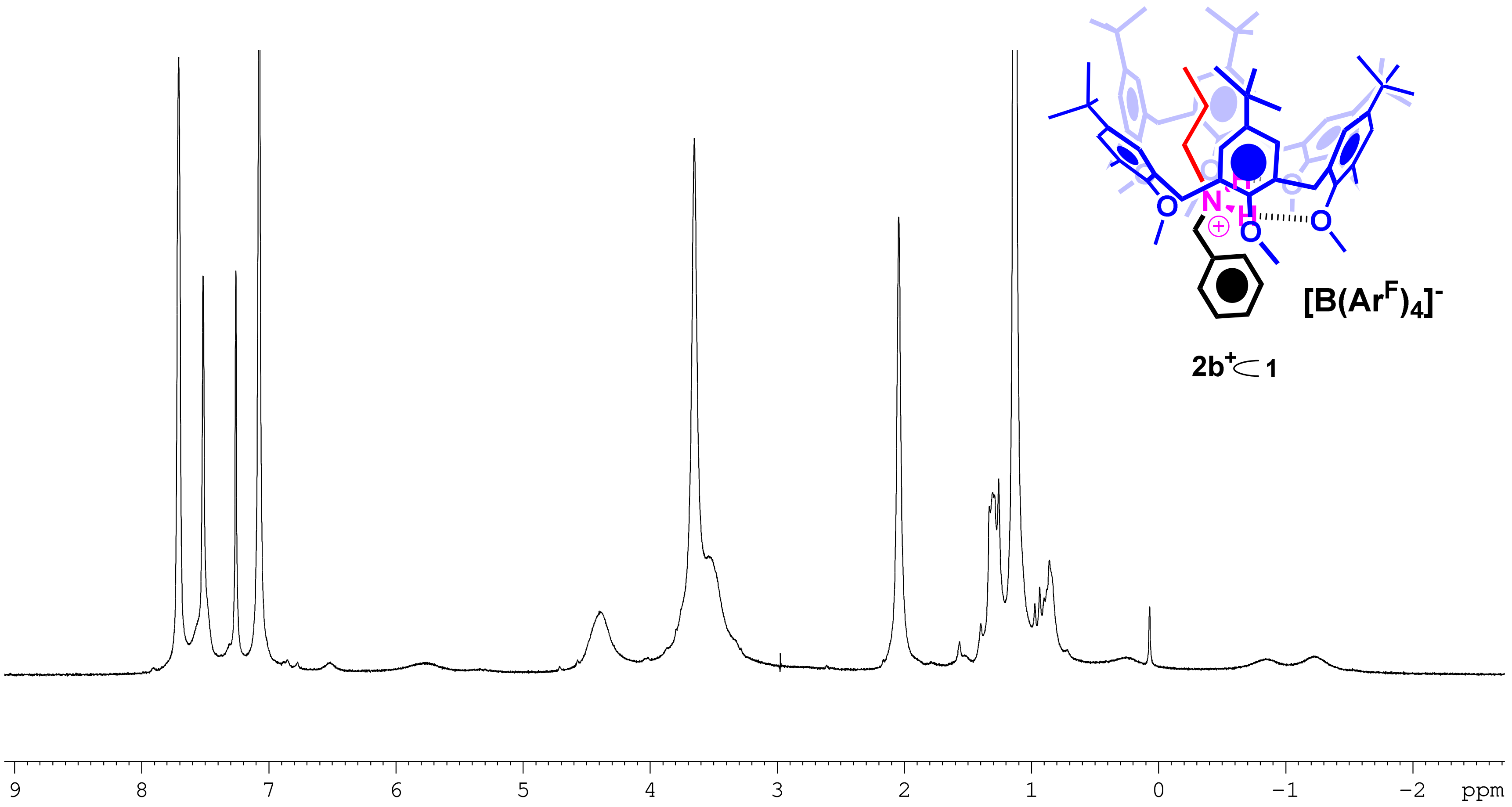

Figure S25. ^{1}H NMR spectrum of **2b$^+$⊂ 1** (400 MHz, $CDCl_3$, 298 K).

^{1}H NMR Spectrum of 2c$^+$⊂ 1

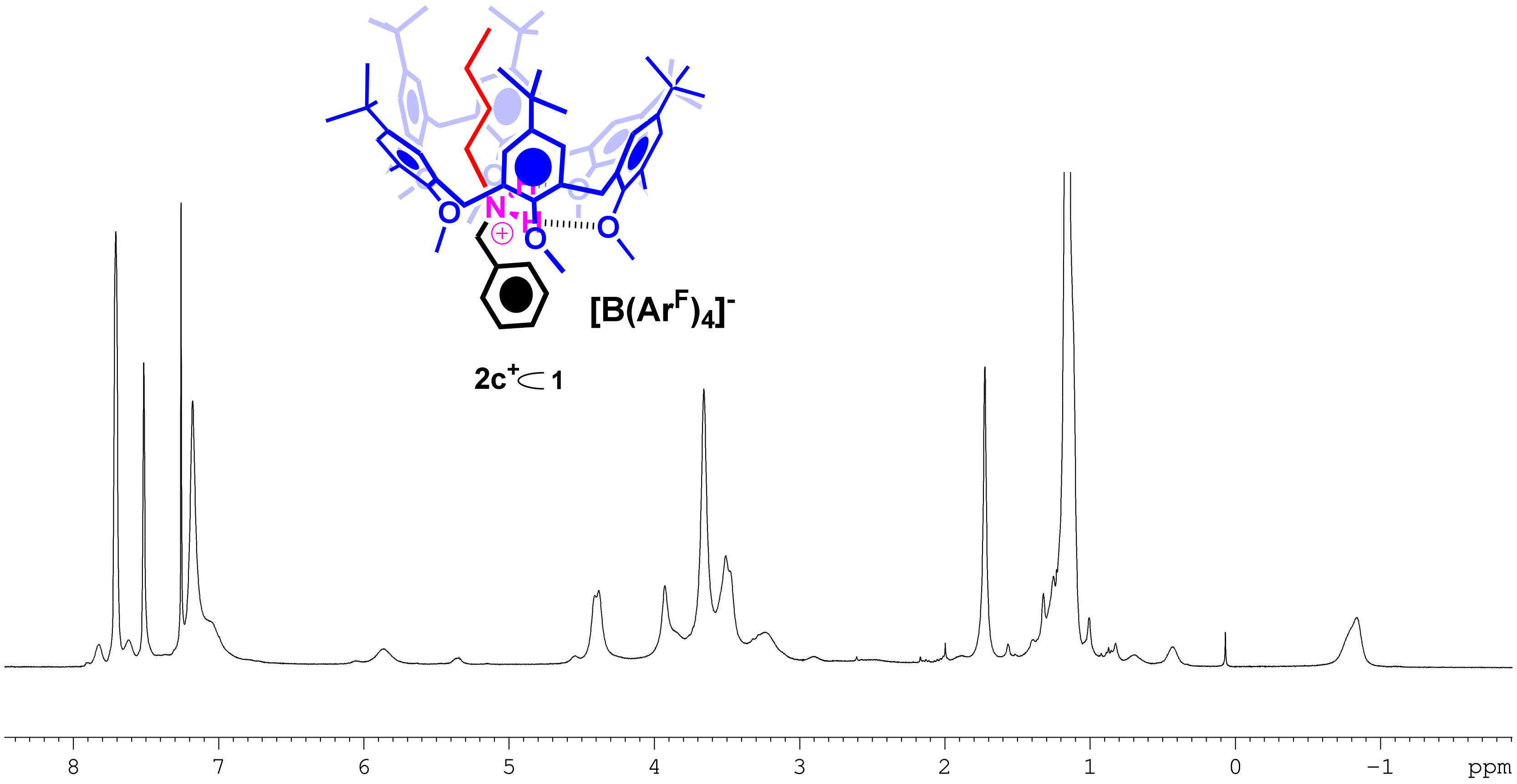

Figure S26. ^{1}H NMR spectrum of **2c$^+$⊂ 1** (400 MHz, $CDCl_3$, 298 K).

^{1}H NMR Spectrum of 2d⁺⊂1

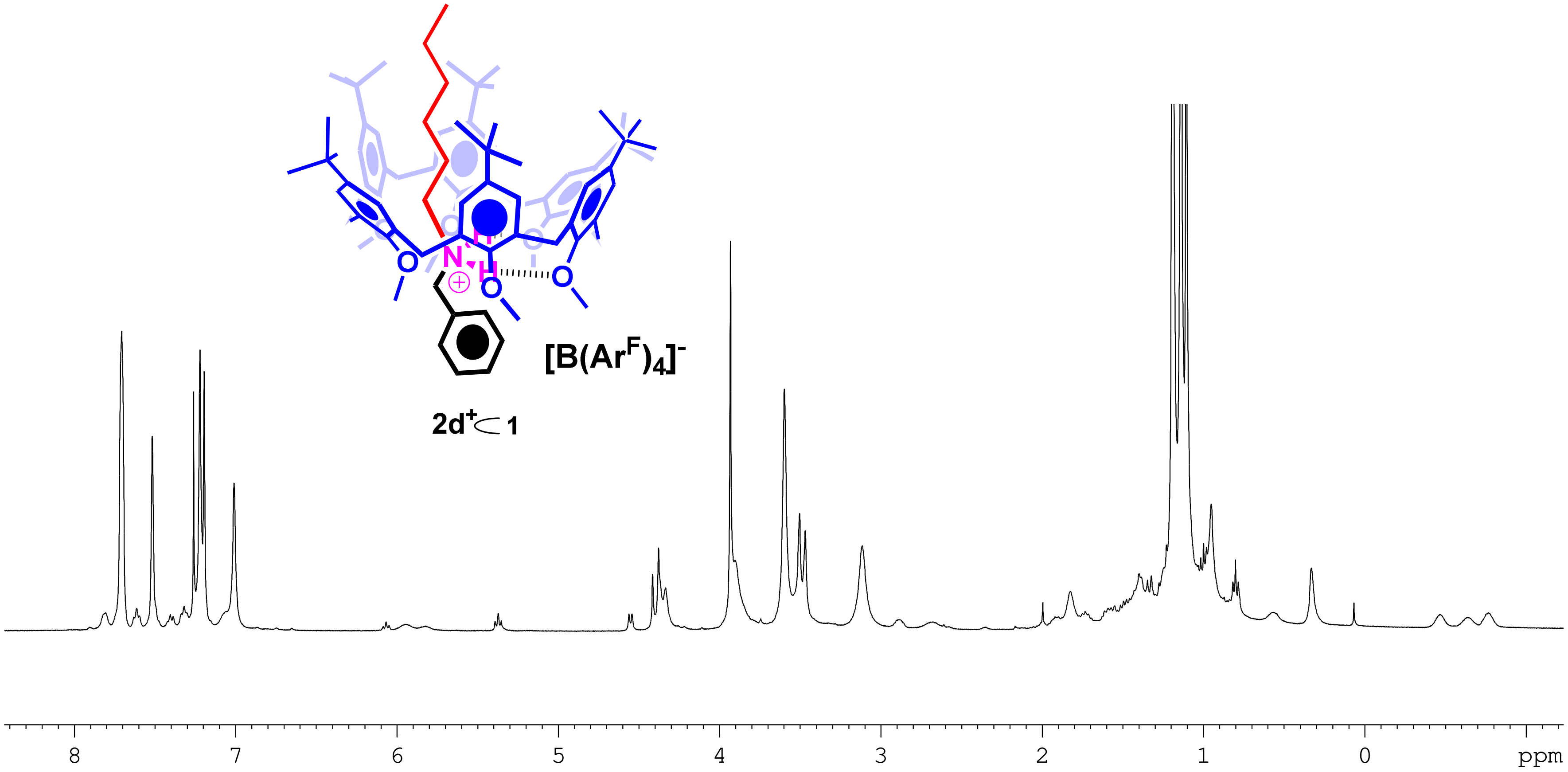

Figure S27. ^{1}H NMR spectrum of **2d$^+$⊂1** (400 MHz, $CDCl_3$, 298 K).

^{1}H NMR Spectrum of 2e^{+}⊂1

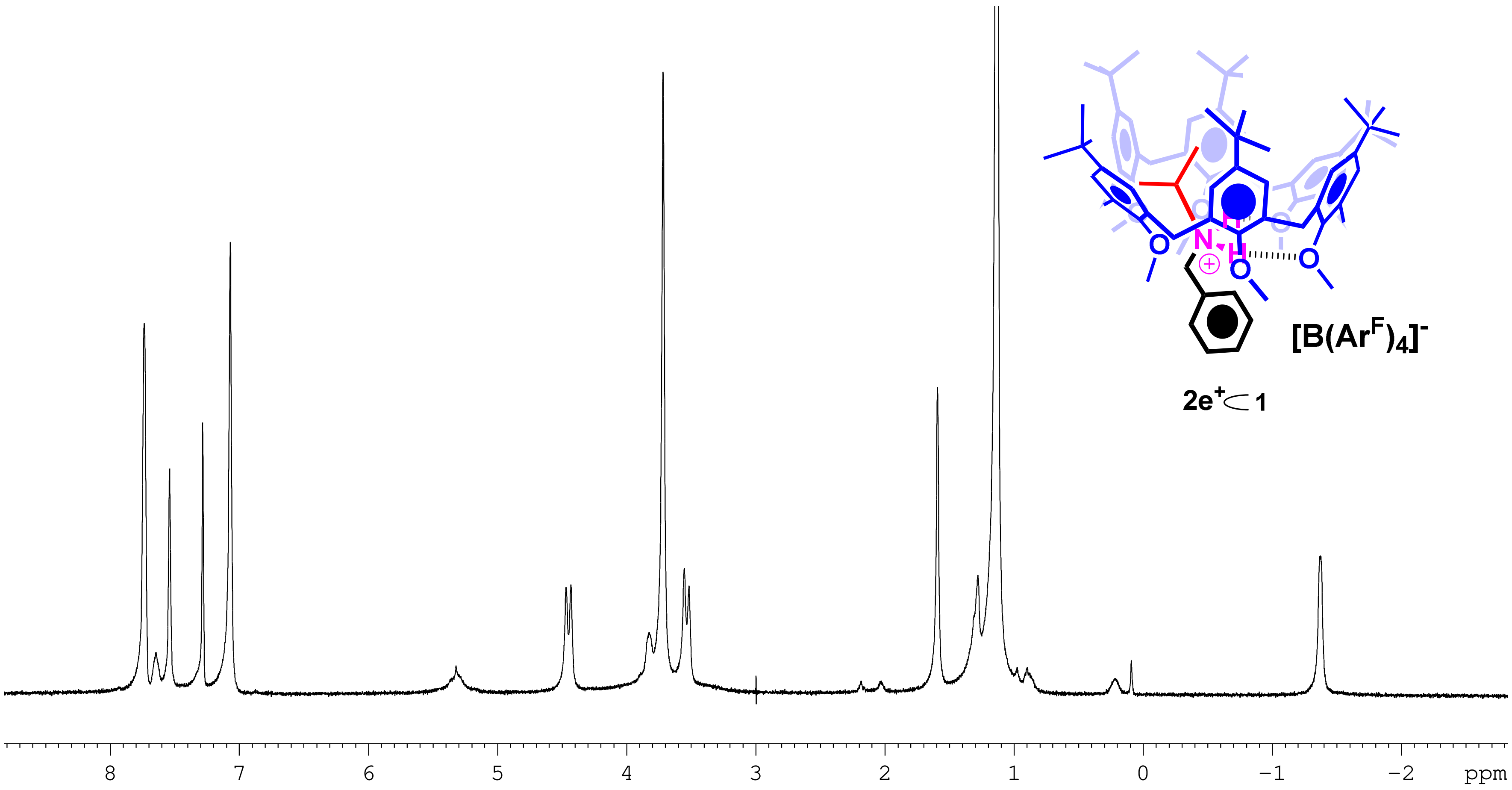

Figure S28. ^{1}H NMR spectrum of **2e^{+}⊂1** (400 MHz, $CDCl_3$, 298 K).

^{1}H NMR Spectrum of 2f^{+}⊂1

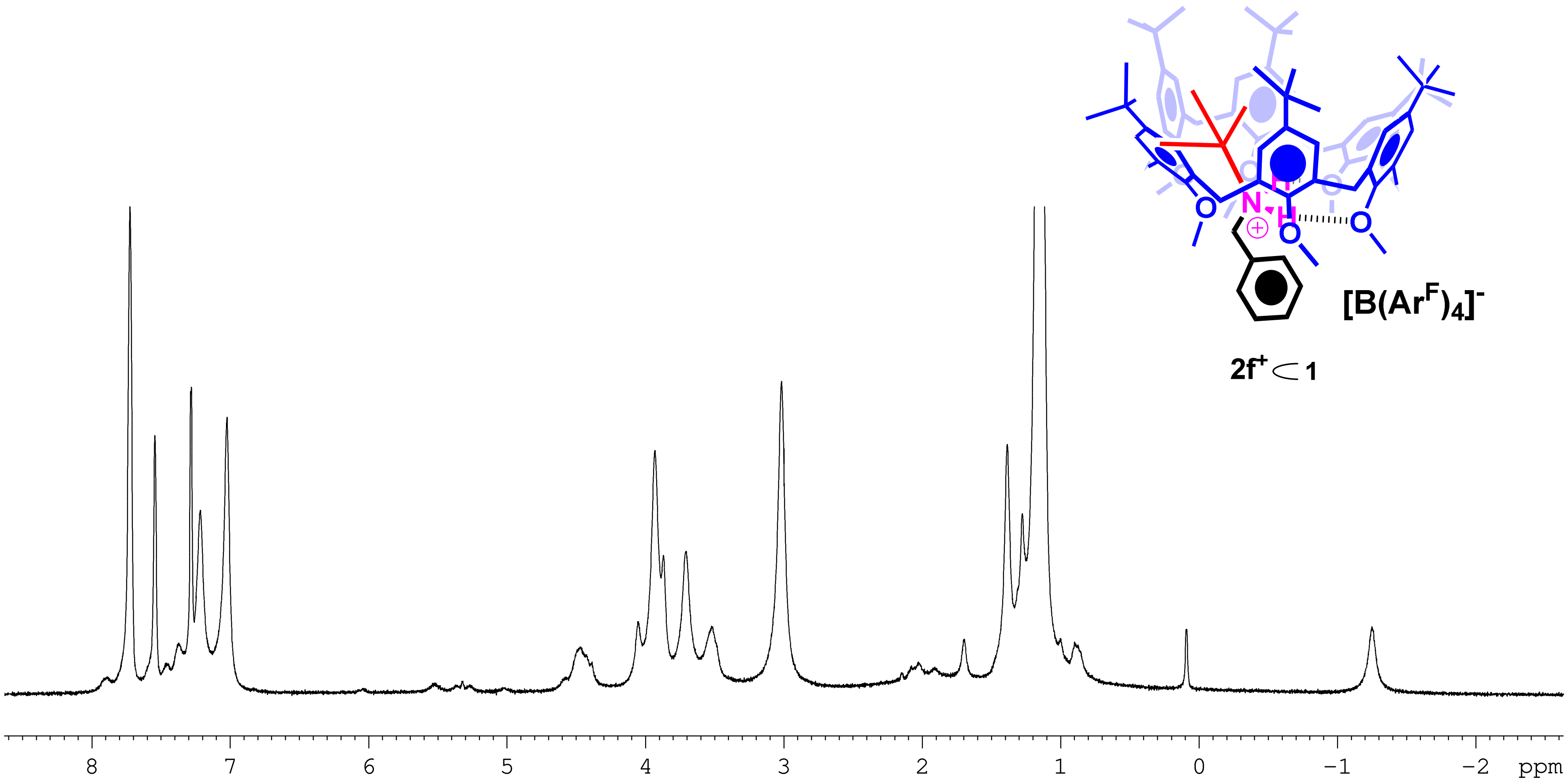

Figure S29. ^{1}H NMR spectrum of **2f^{+}⊂1** (400 MHz, $CDCl_3$, 298 K).

^{1}H NMR Spectrum of 2g⁺⊂ 1

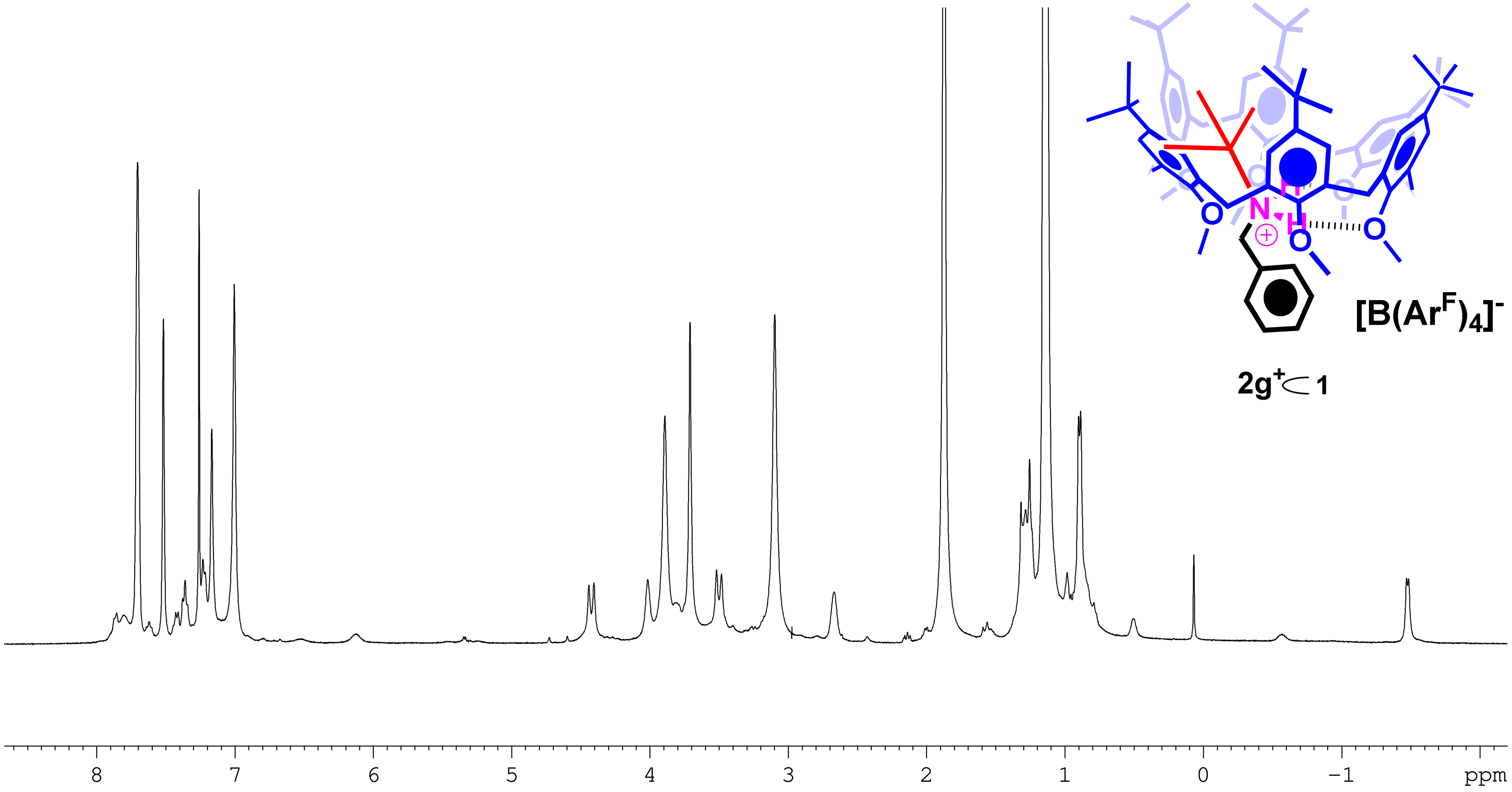

Figure S30. ^{1}H NMR spectrum of **2g⁺⊂ 1** (400 MHz, $CDCl_3$, 298 K).

^{1}H NMR Spectrum of 2h^{+}⊂1

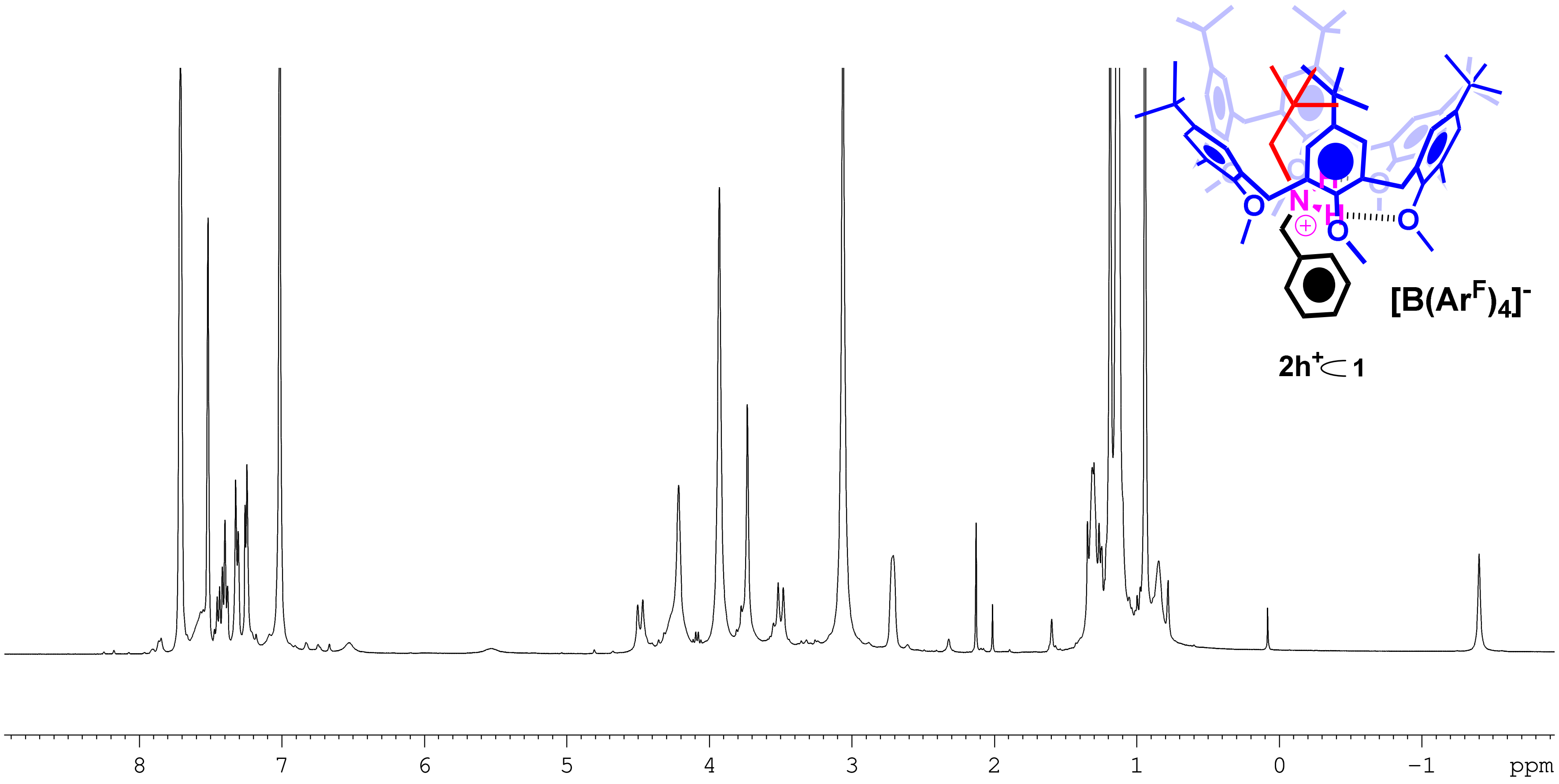

Figure S31. ^{1}H NMR spectrum of **2h^{+}⊂1** (400 MHz, $CDCl_3$, 298 K).

^{1}H NMR Spectrum of 2i⁺⊂ 1

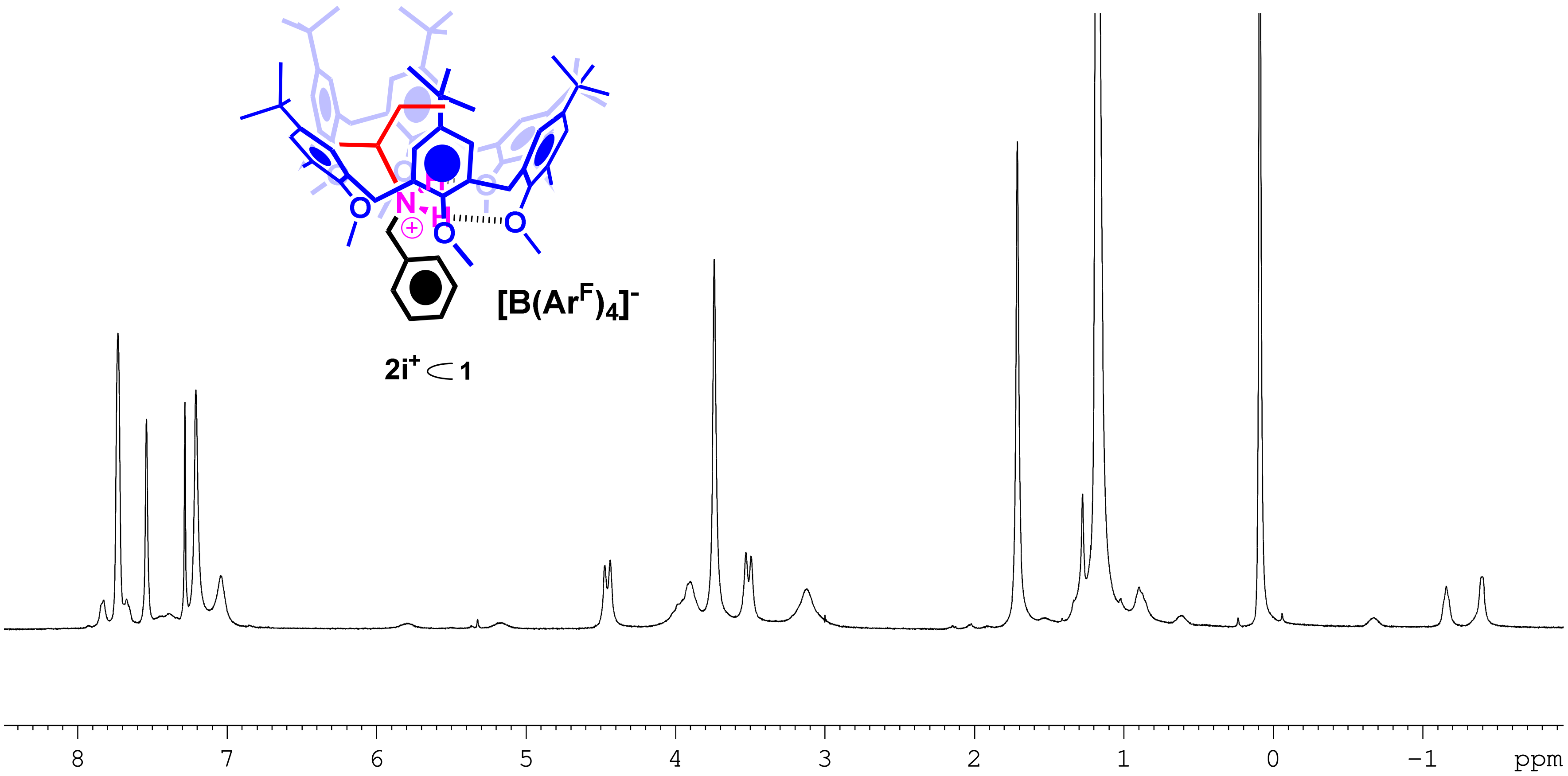

Figure S32. ^{1}H NMR spectrum of **2i$^+$⊂ 1** (400 MHz, $CDCl_3$, 298 K).

^{1}H NMR Spectrum of 2j^{+}⊂1

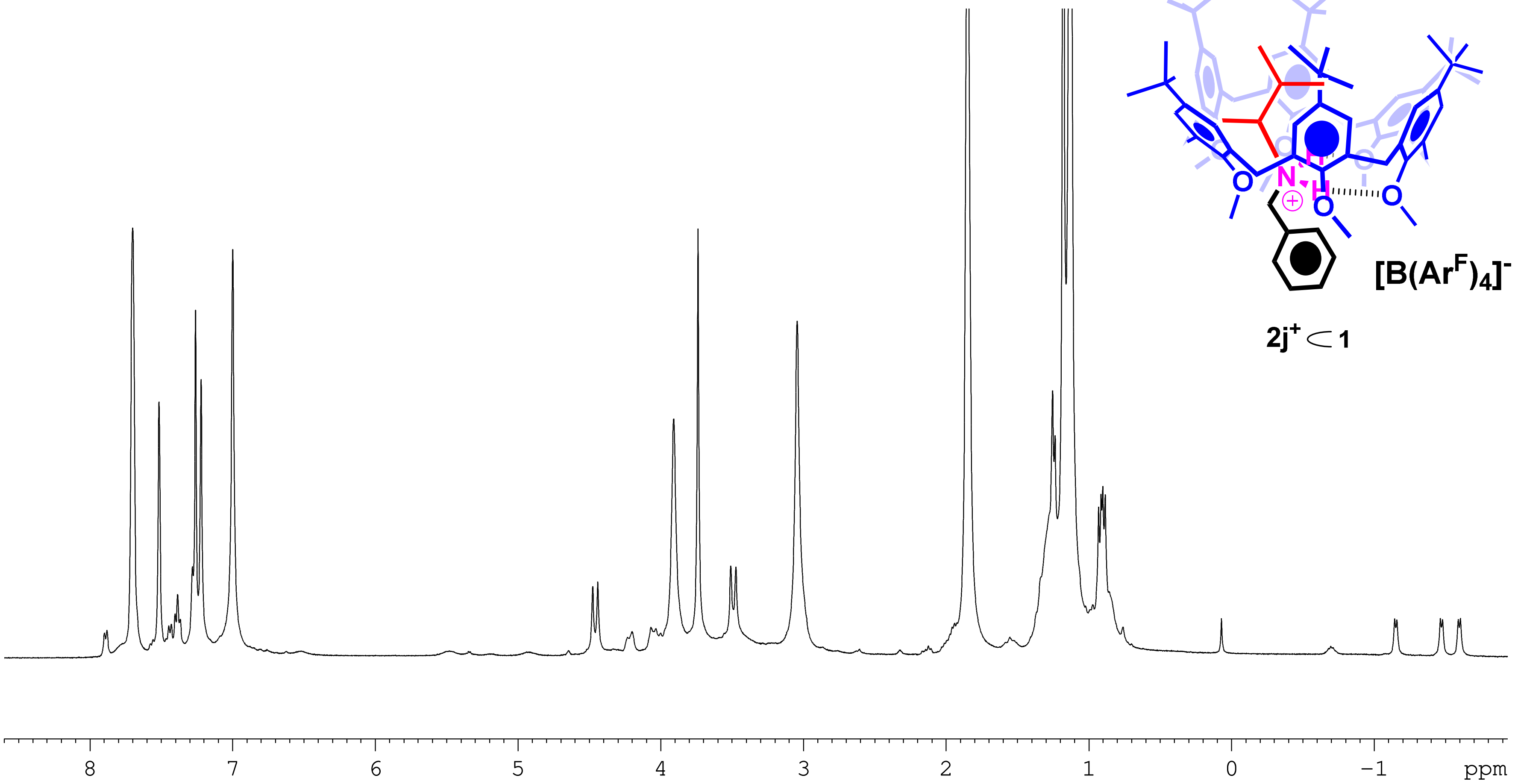

Figure S33. ^{1}H NMR spectrum of **2j^{+}⊂1** (400 MHz, $CDCl_3$, 298 K).

^{1}H NMR Spectrum of 2k^{+}⊂ 1

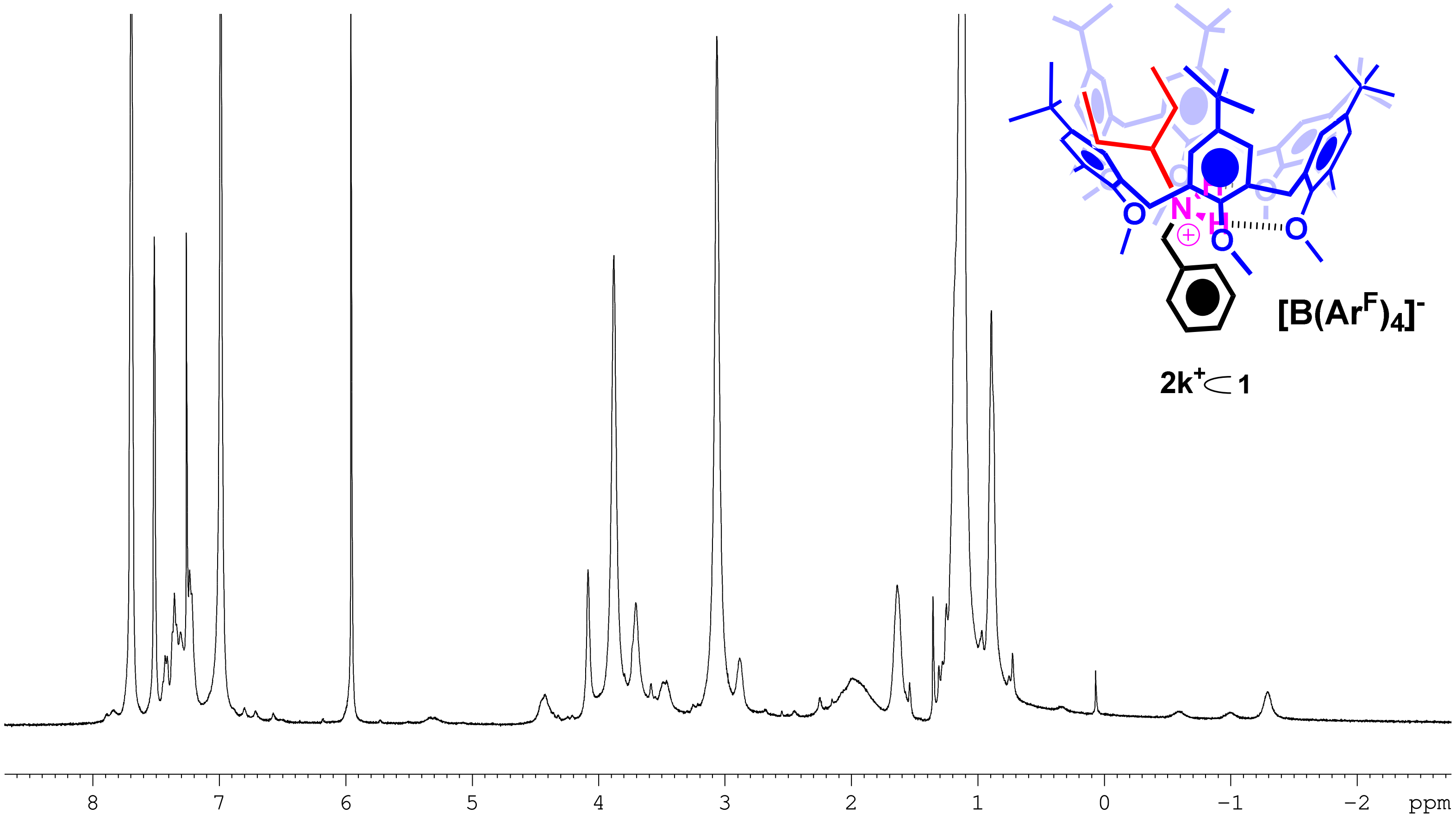

Figure S34. ^{1}H NMR spectrum of **2k^{+}⊂ 1** (400 MHz, $CDCl_3$, 298 K).

^{1}H NMR determination of K$_{ass}$ values

The association constant values of complexes were calculated by means of three methods:

a) **^{1}H NMR competition experiments**. In this case, a ^{1}H NMR analysis was performed on a 1:1:1 mixture of host, and two guests (or two host and one guest) in a NMR tube using 0.5 mL of $CDCl_3$ as solvent. The following equation was used to obtain the K$_{ass}$ value.

$$K_{A\subset H}=\frac{[HG_A]}{[H][G_A]} \quad \text{and} \quad K_{B\subset H}=\frac{[HG_B]}{[H][G_B]} \longrightarrow K_{rel}=\frac{K_{A\subset H}}{K_{B\subset H}}=\frac{[HG_A][H][G_B]}{[HG_B][H][G_A]} \longrightarrow$$

$$\begin{matrix}[HG_A]=[G_B]\\ [HG_B]=[G_A]\end{matrix} \longrightarrow K_{rel}=\frac{K_{A\subset H}}{K_{B\subset H}}=\frac{[HG_A]^2}{[HG_B]^2}$$

b) **Quantitative ^{1}H NMR experiments using 1,1,2,2-tetrachloroethane as the internal standard**. In this case, ^{1}H NMR experiments were carried out on a 1:1 mixture of host and guest in 0.5 mL of $CDCl_3$ containing 1 ◘L of 1,1,2,2-tetrachloroethane (d= 1.586 g/mL) as internal standard. The following equation was used to obtain the moles of the complex:

$$\frac{G_a}{G_b}=\frac{F_a}{F_b}\times\frac{N_b}{N_a}\times\frac{M_a}{M_b}$$

Where:

G_a = grams of 1,1,2,2-Tetrachloroethane**;** G_b= grams of pseudorotaxane.

F_a and F_b= areas of the signal related of 1,1,2,2-tetrachloroethane and pseudorotaxane.

N_a and N_b = numbers of nuclei which cause the signals (N_a for 1,1,2,2-tetrachloroethane**;** N_b for pseudorotaxane)

M_a and M_b = molecular masses of 1,1,2,2-tetrachloroethane (a) and pseudorotaxane (b).

c) **Integration of free and complexed ^{1}H NMR signals of host or guest**. In this case, an equimolar solution of host and guest was solubilized in $CDCl_3$ and equilibrated in a NMR tube.

Apparent association constants of 2a-k$^+$⊂1 pseudorotaxanes

Axle	K_{app} (M^{-1})	logK_{app}
**2a$^+$⊂1**	1.1 ±0.2×10^6	6.04
**2b$^+$⊂1**	4.8 ±0.8×10^3	3.68
**2c$^+$⊂1**	6.5 ±0.9×10^4	4.81
**2d$^+$⊂1**	2.4 ±0.6×10^3	3.38
**2e$^+$⊂1**	4.2 ±0.6×10^4	4.62
**2f$^+$⊂1**	3.6 ±0.5×10^2	2.56
**2g$^+$⊂1**	5.1 ±0.6×10^3	3.71
**2h$^+$⊂1**	1.7 ±0.6×10^3	3.23
**2i$^+$⊂1**	6.9 ±0.8×10^3	3.84
**2j$^+$⊂1**	2.9 ±0.5×10^3	3.46
**2k$^+$⊂1**	2.7 ±0.4×10^2	2.43

Table 1.

K_{ass} value of 2a⁺⊂ 1

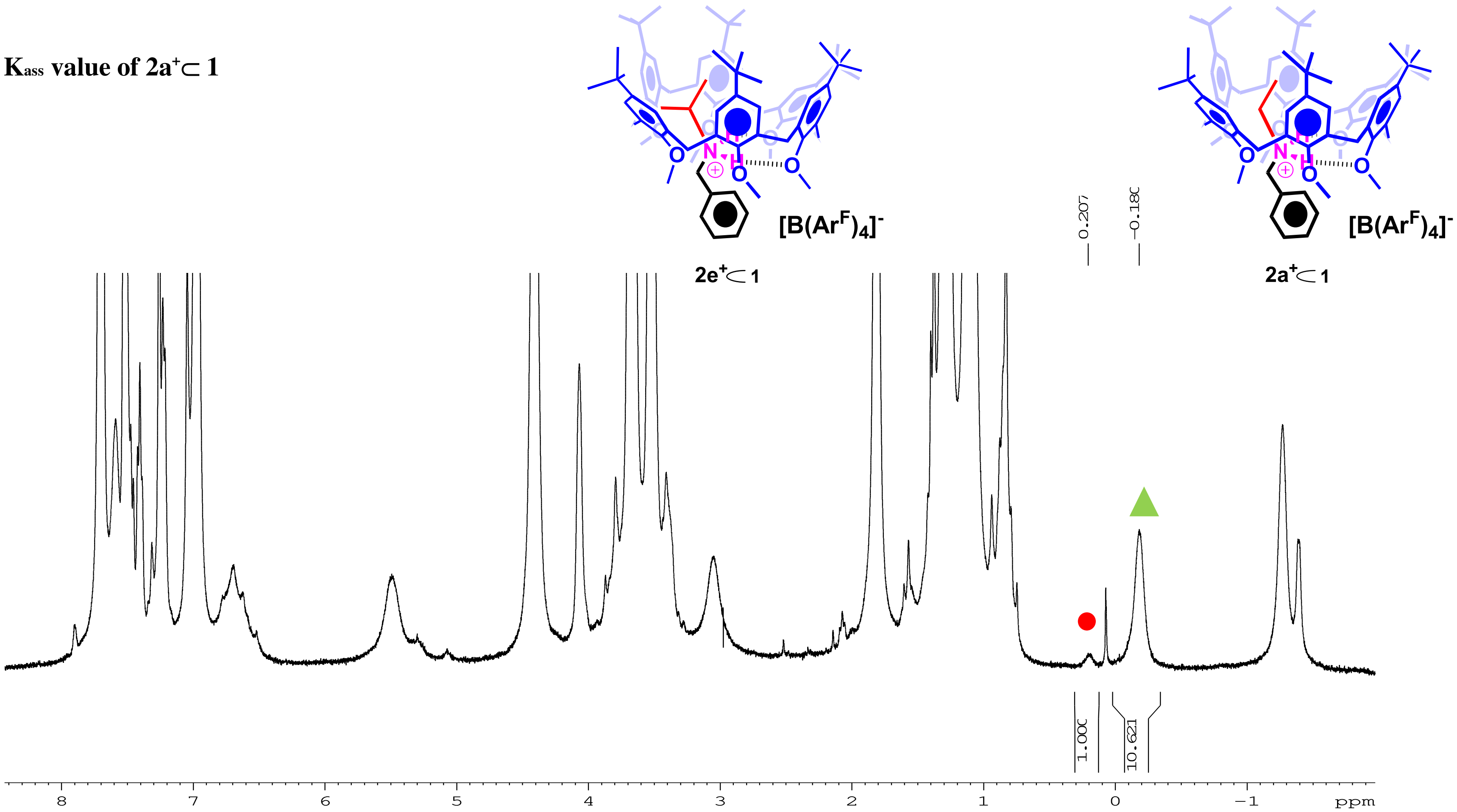

Figure S35. ^{1}H NMR spectrum ($CDCl_3$, 400 MHz, 298 K) of derivative 1 in the presence of 1 equivalent of 2a$^+$ and 1 equivalent of 2e$^+$ (3.8·10^{-3} M each one). (●) resonance relative to 2e$^+$⊂ 1$^-$complex, (▲) resonance relative to 2a$^+$⊂ 1 $^-$ complex.

K$_{ass}$ value of 2b^{+}⊂ 1

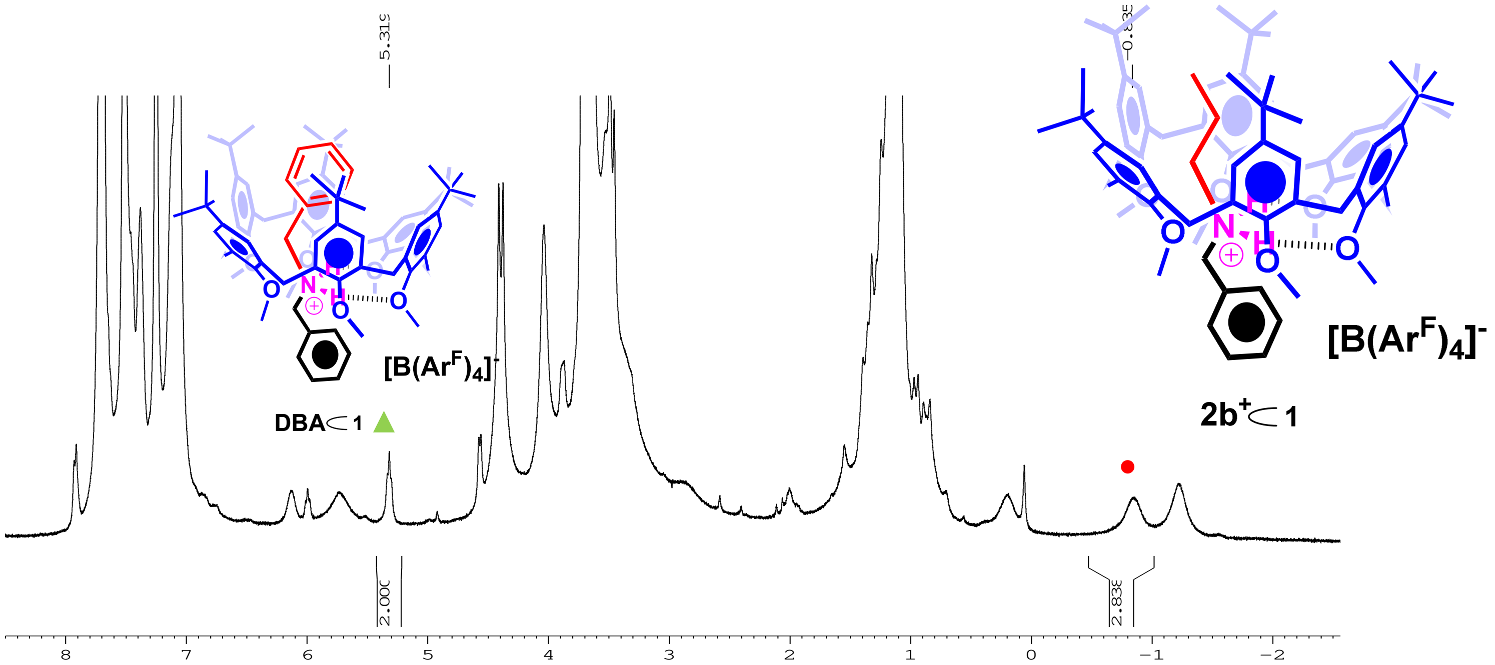

Figure S38. ^{1}H NMR spectrum (CDCl$_3$, 400 MHz, 298 K) of derivative **1** in the presence of 1 equivalent of **2b^{+-}** and 1 equivalent of **dibenzylammoniun (DBA)** axle[2] (3.8·10^{-3} M each one) . (●) Resonance relative to the *endo*-alkyl-**2b^{+}⊂ 1**complex, (▲) resonance relative to **DBA⊂ 1** complex.

K$_{ass}$ value of 2c$^+$⊂ 1

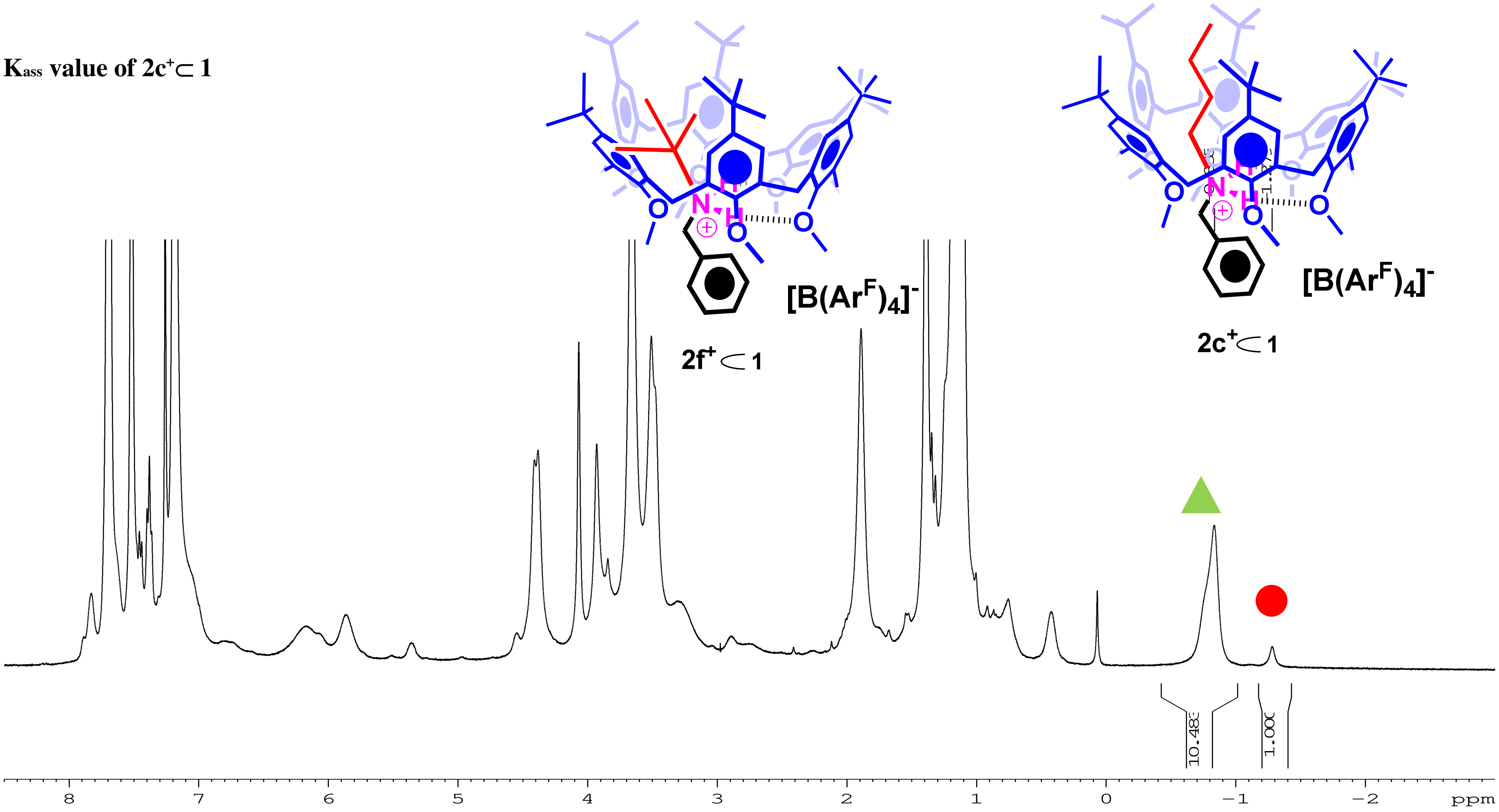

Figure S37. ^{1}H NMR spectrum ($CDCl_3$, 400 MHz, 298 K) of derivative **1** in the presence of 1 equivalent of **2c$^+$** and 1 equivalent of **2f$^+$** ($3.8 \cdot 10^{-3}$ M each one). (●) Resonance relative to **2f$^+$⊂ 1** complex, (▲) resonance relative to **2c$^+$⊂ 1**.

K_{ass} value of 2d^{+}⊂ 1

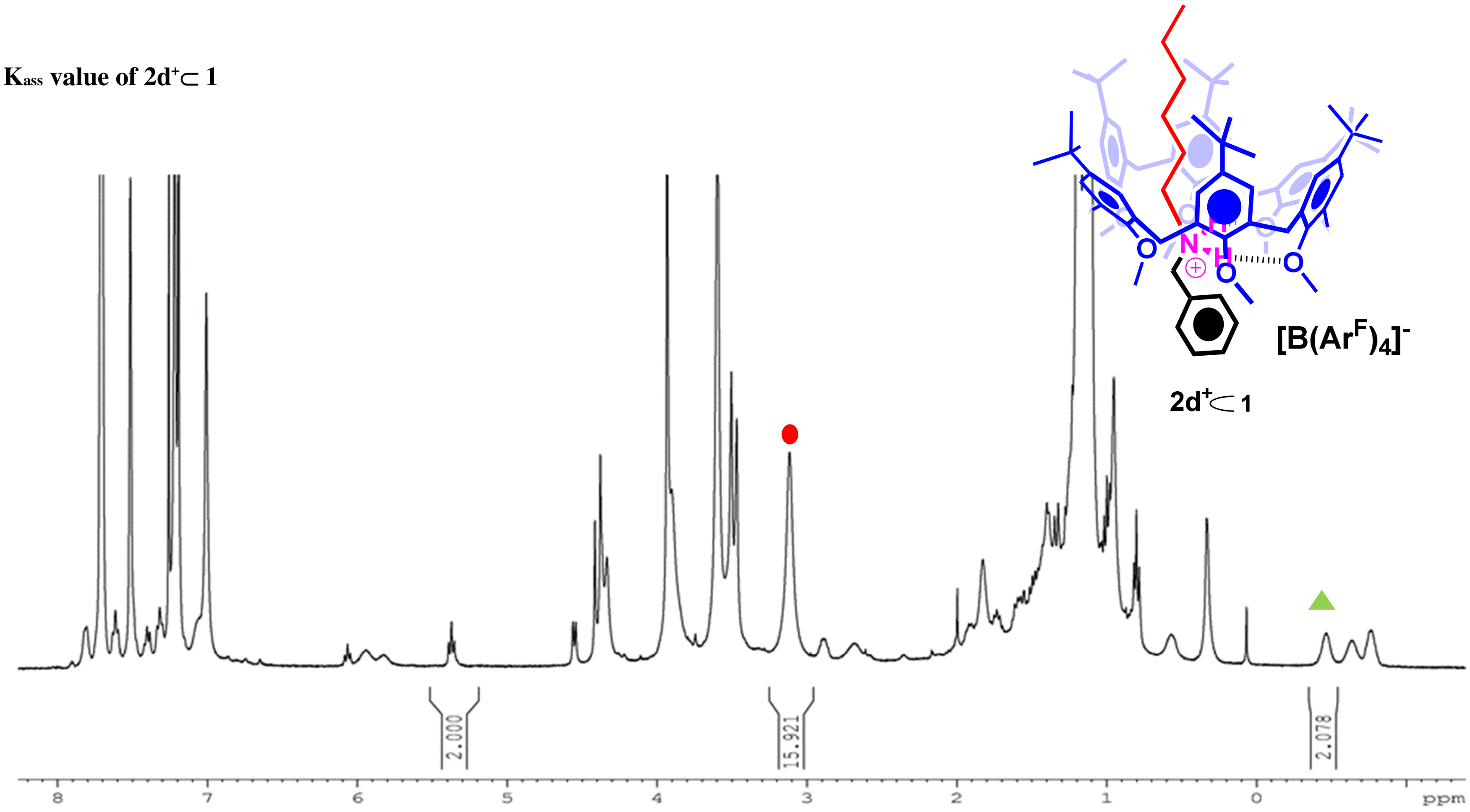

Figure S37. ^{1}H NMR spectrum (CDCl$_3$, 400 MHz, 298 K) of equimolar solution (3.8·10^{-3} M) of **1** and **2d^{+}**. The association constant K_{ass} value was calculated by integration of complexed **2d^{+}⊂ 1** (▲) and free derivative **1** (●).

K$_{ass}$ value of 2e^{+}⊂ 1

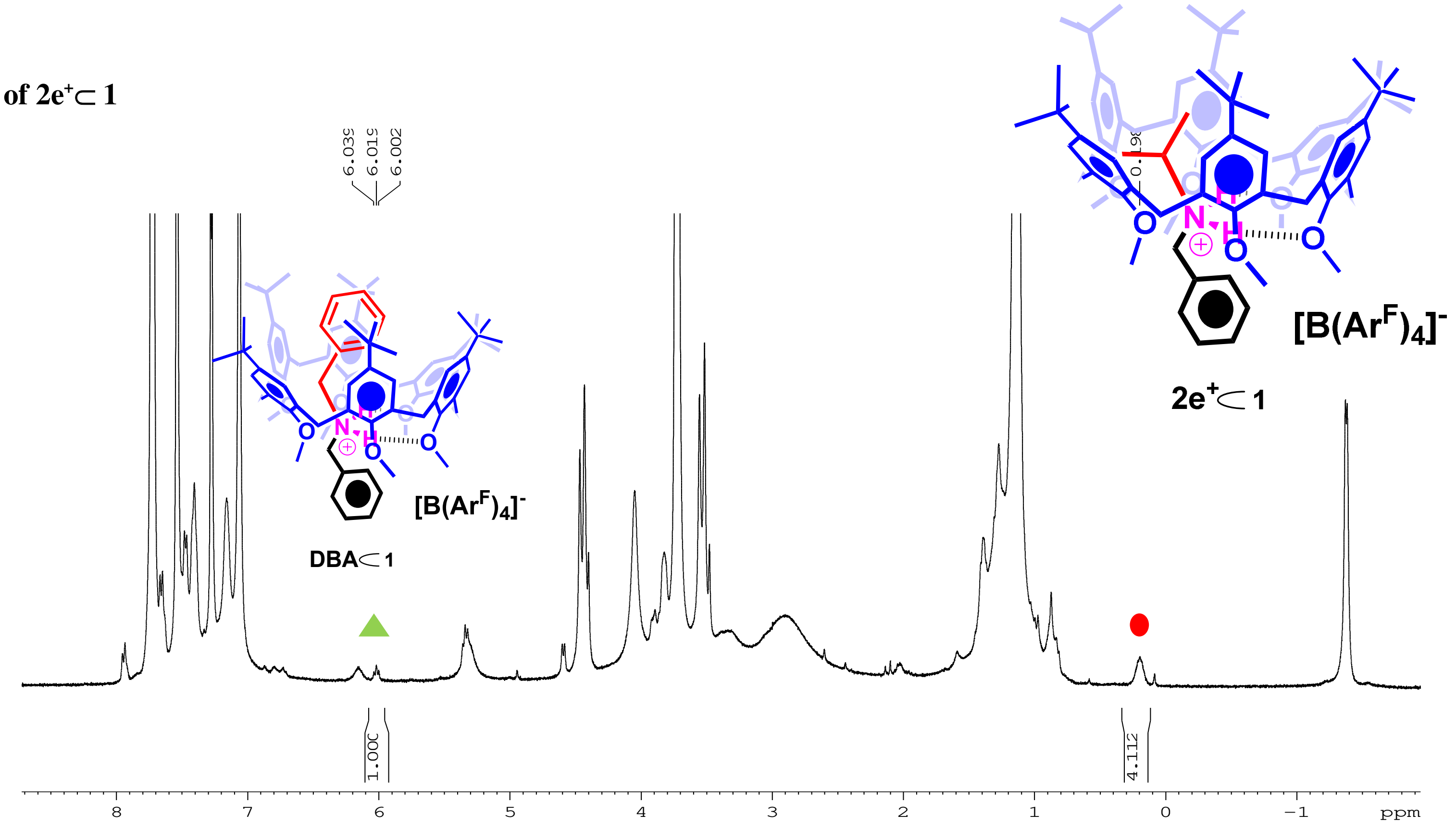

Figure S38. ^{1}H NMR spectrum ($CDCl_3$, 400 MHz, 298 K) of derivative **1** in presence of 1 equivalent of **2e^{+}** and 1 equivalent of **dibenzylammonium (DBA)** axle (3.8·10^{-3} M each one). (●) Resonance relative to the *endo*-alkyl-**2e^{+}⊂ 1**complex, (▲) resonance relative to **DBA⊂ 1** complex.

Kass value of 2f⁺⊂ 1

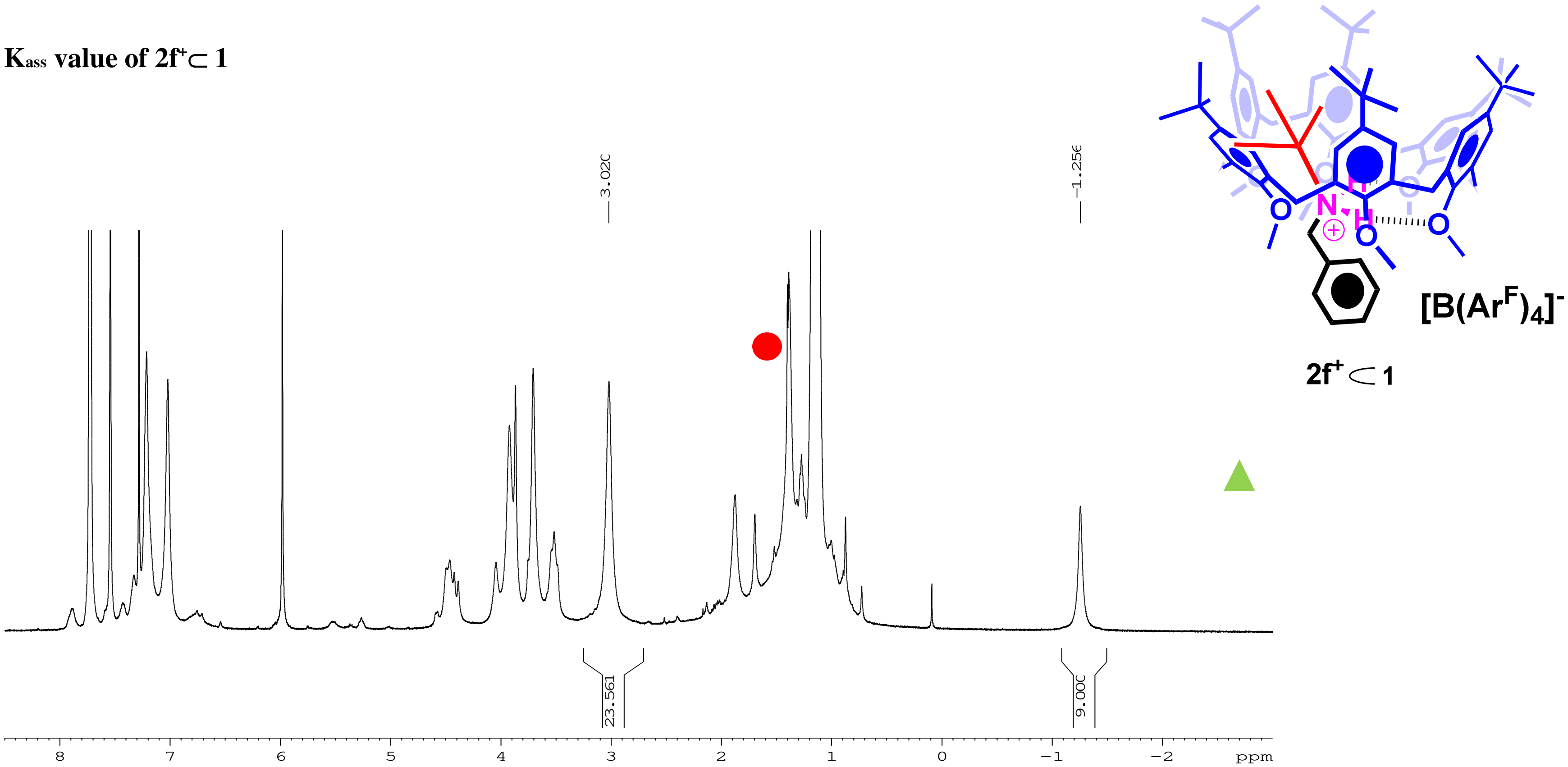

Figure S39. ¹H NMR spectrum ($CDCl_3$, 400 MHz, 298 K) of equimolar solution ($3.8\cdot10^{-3}$ M) of 1 and 2f⁺. The association constant K_{ass} value was calculated by integration of complexed 2f⁺⊂ 1 (▲) and free derivative 1 (●).

K$_{ass}$ value of 2g^{+}⊂ 1

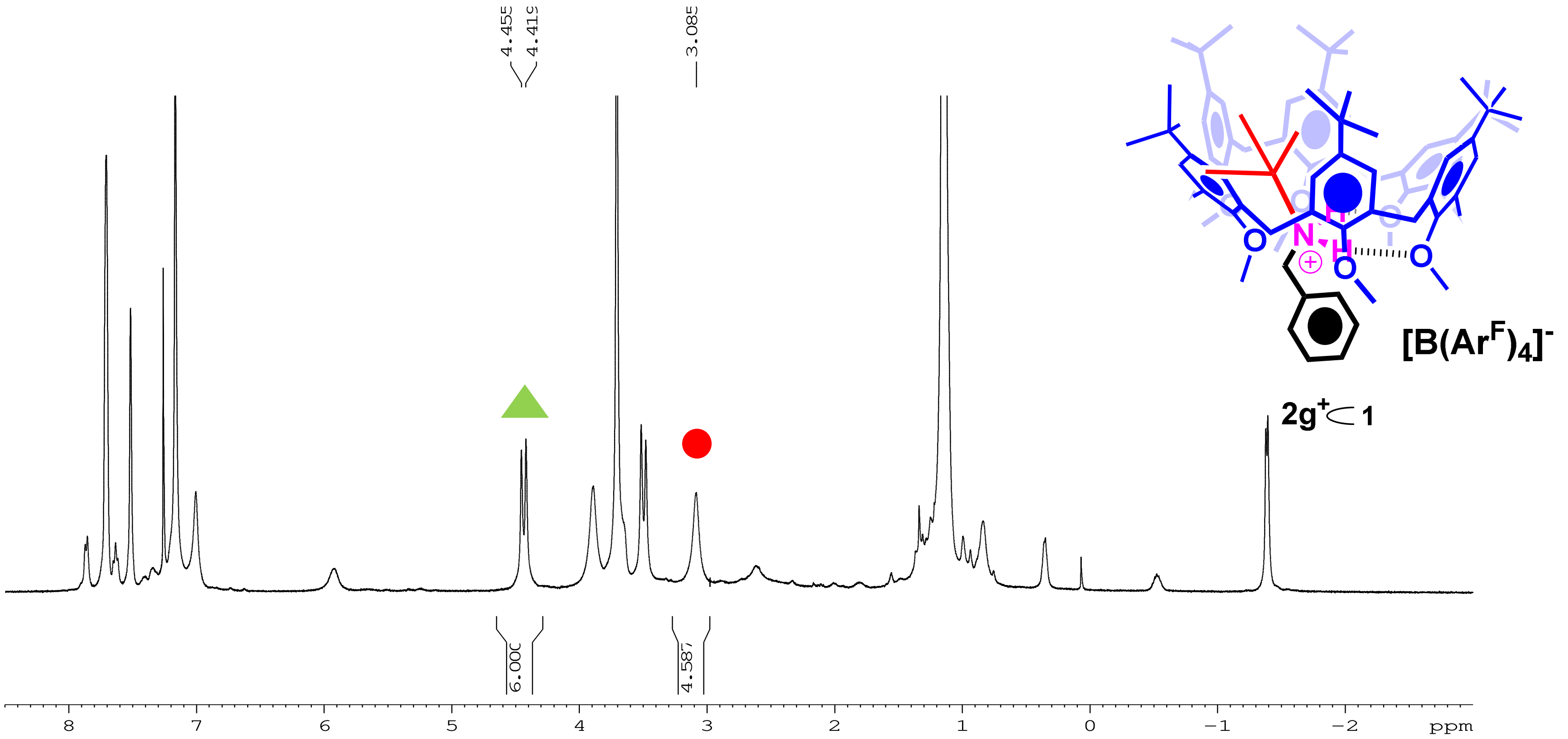

Figure S40. ^{1}H NMR spectrum ($CDCl_3$, 400 MHz, 298 K) of equimolar solution ($3.8 \cdot 10^{-3}$ M) of **1** and **2g**$^{+}$. The association constant ***K$_{ass}$*** value was calculated by integration of complexed (▲) and free (●) derivative **1**.

K_{ass} value of $2h^{+}\subset 1$

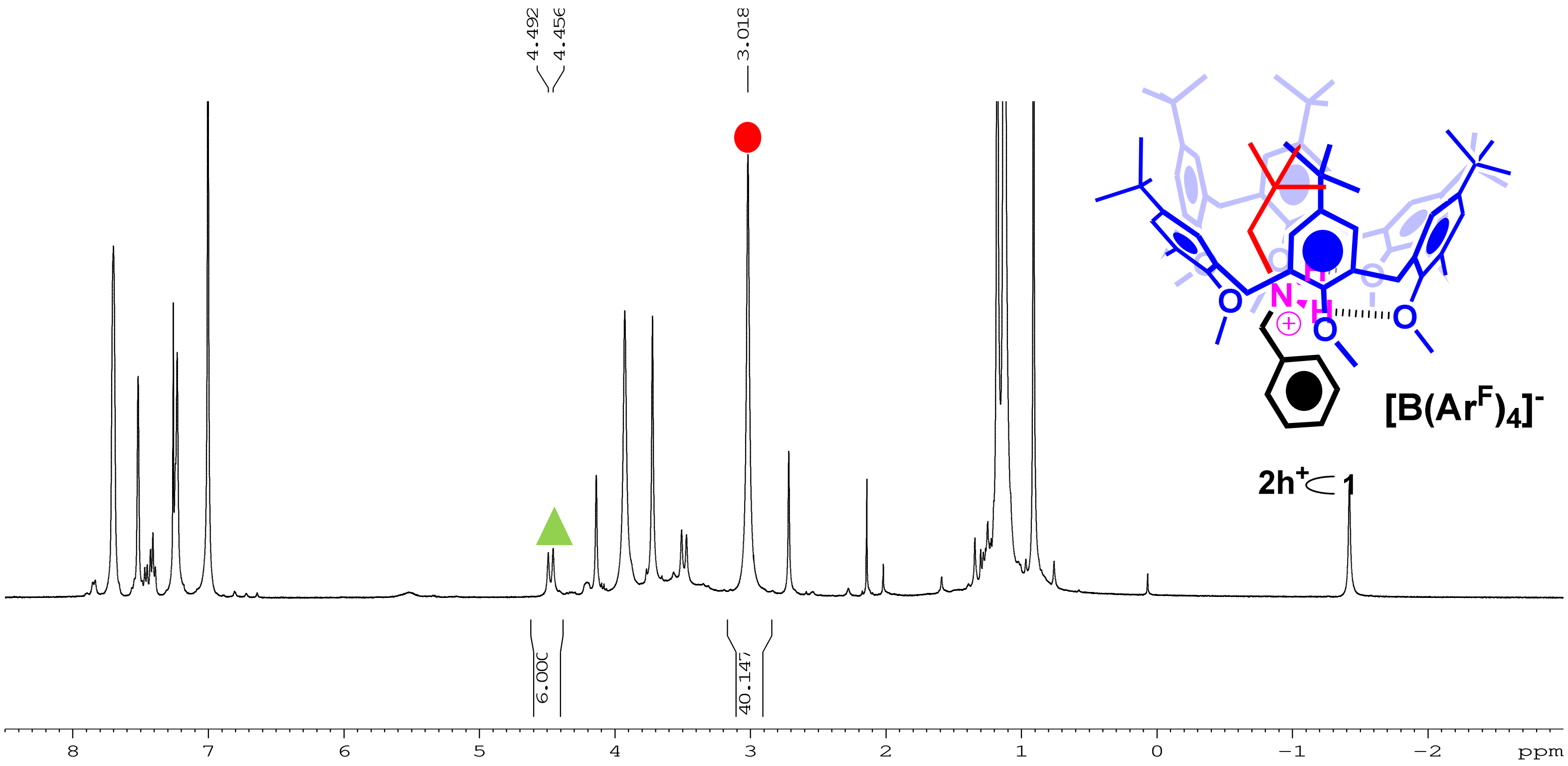

Figure S41. ^{1}H NMR spectrum ($CDCl_3$, 400 MHz, 298 K) of equimolar solution ($3.8\cdot10^{-3}$ M) of **1** and **2h**$^+$. The association constant $\boldsymbol{K_{ass}}$ value was calculated by integration of complexed (▲) and free (●) derivative **1**.

K_{ass} value of $2i^{+}\subset 1$

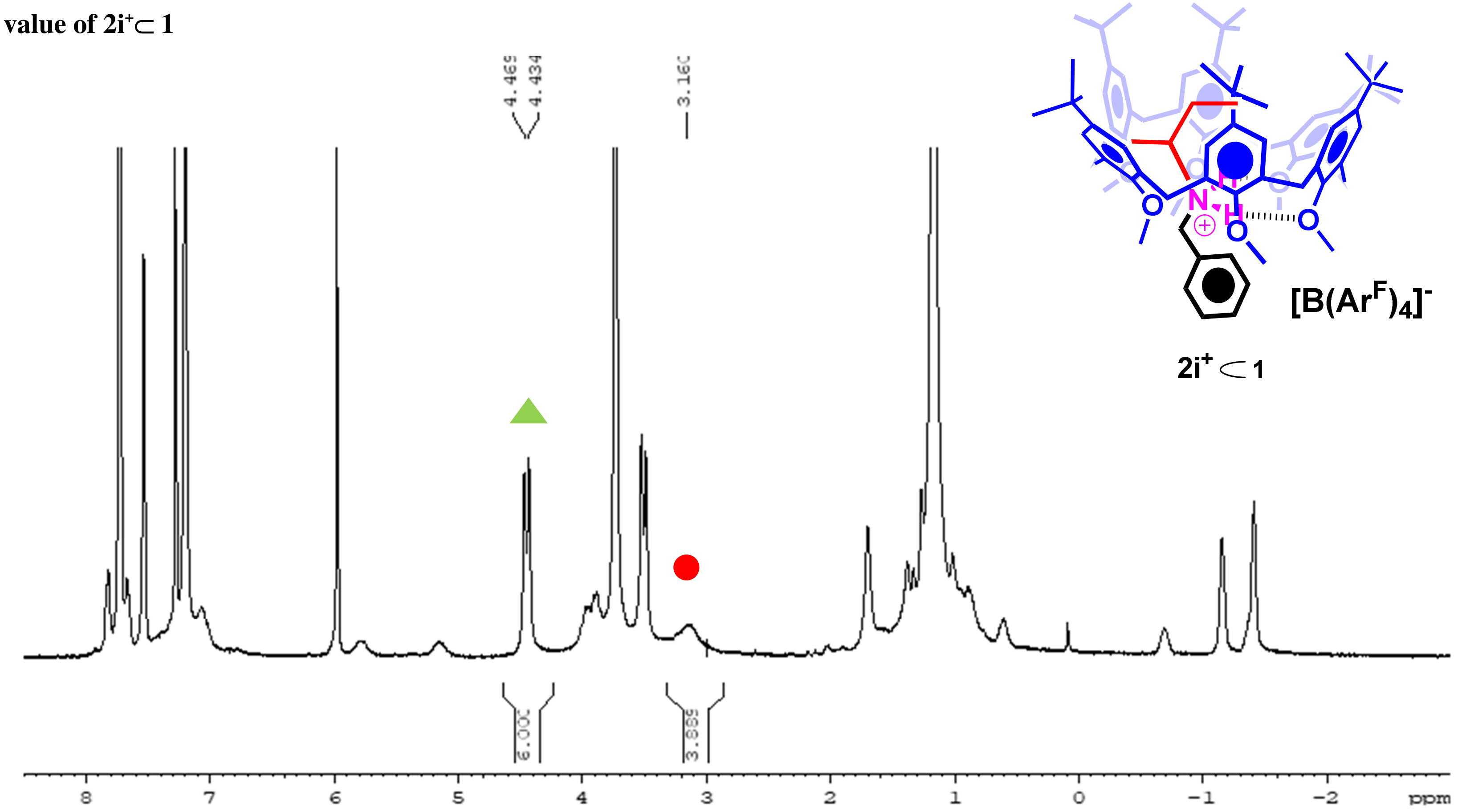

Figure S42. ^{1}H NMR spectrum ($CDCl_3$, 400 MHz, 298 K) of equimolar solution ($3.8\cdot10^{-3}$ M) of **1** and **$2i^{+}\subset 1$.** The association constant $\boldsymbol{K}_{ass}$ value was calculated by integration of complexed (▲) and free (●) derivative **1**.

K_{ass} value of $2j^+ \subset 1$

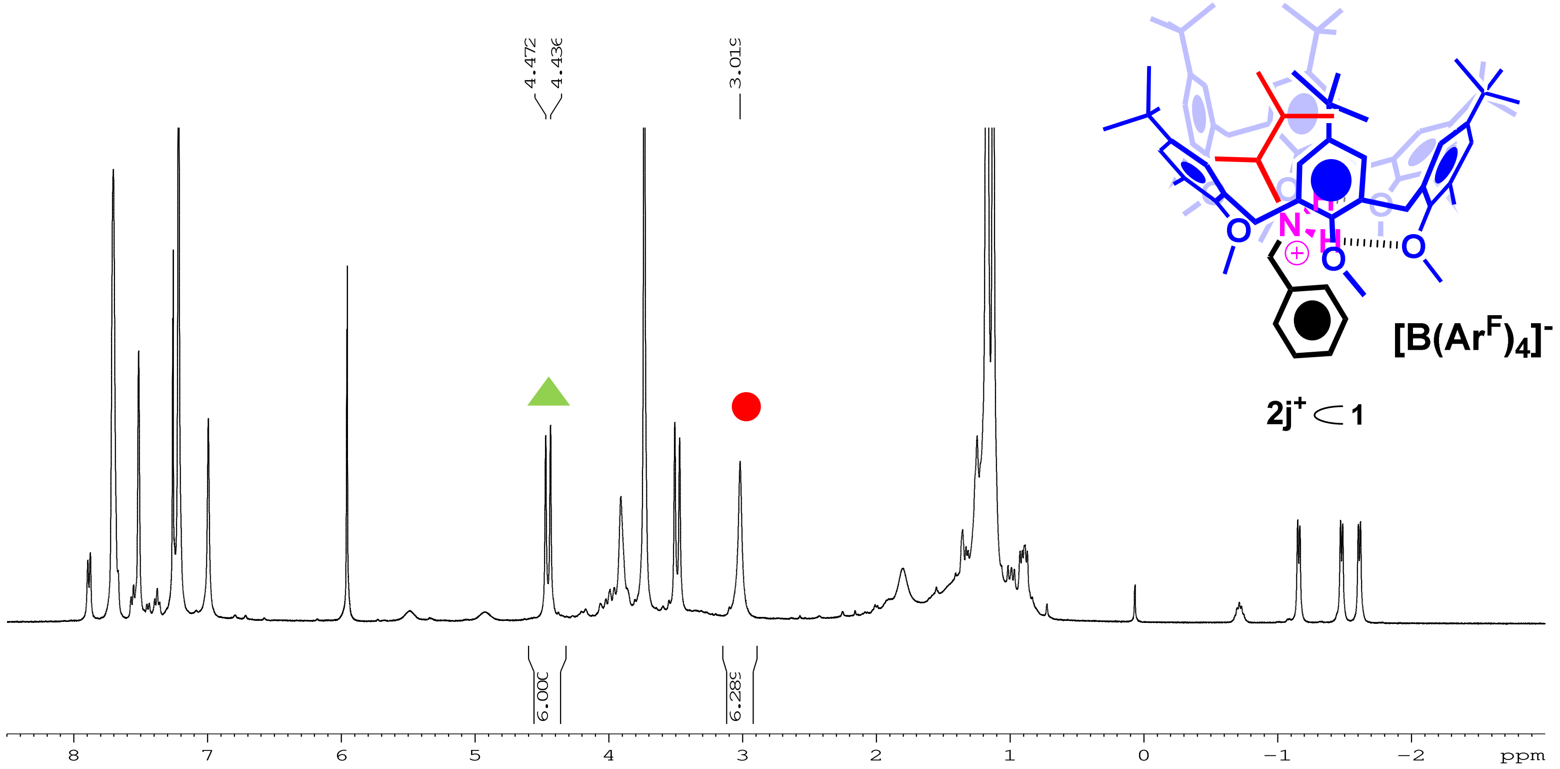

Figure S43. ^{1}H NMR spectrum ($CDCl_3$, 400 MHz, 298 K) of equimolar solution ($3.8 \cdot 10^{-3}$ M) of **1** and **$2j^+ \subset 1$.** The association constant K_{ass} value was calculated by integration of complexed (▲) and free (●) derivative **1**.

K_{ass} value of 2k$^+$⊂ 1

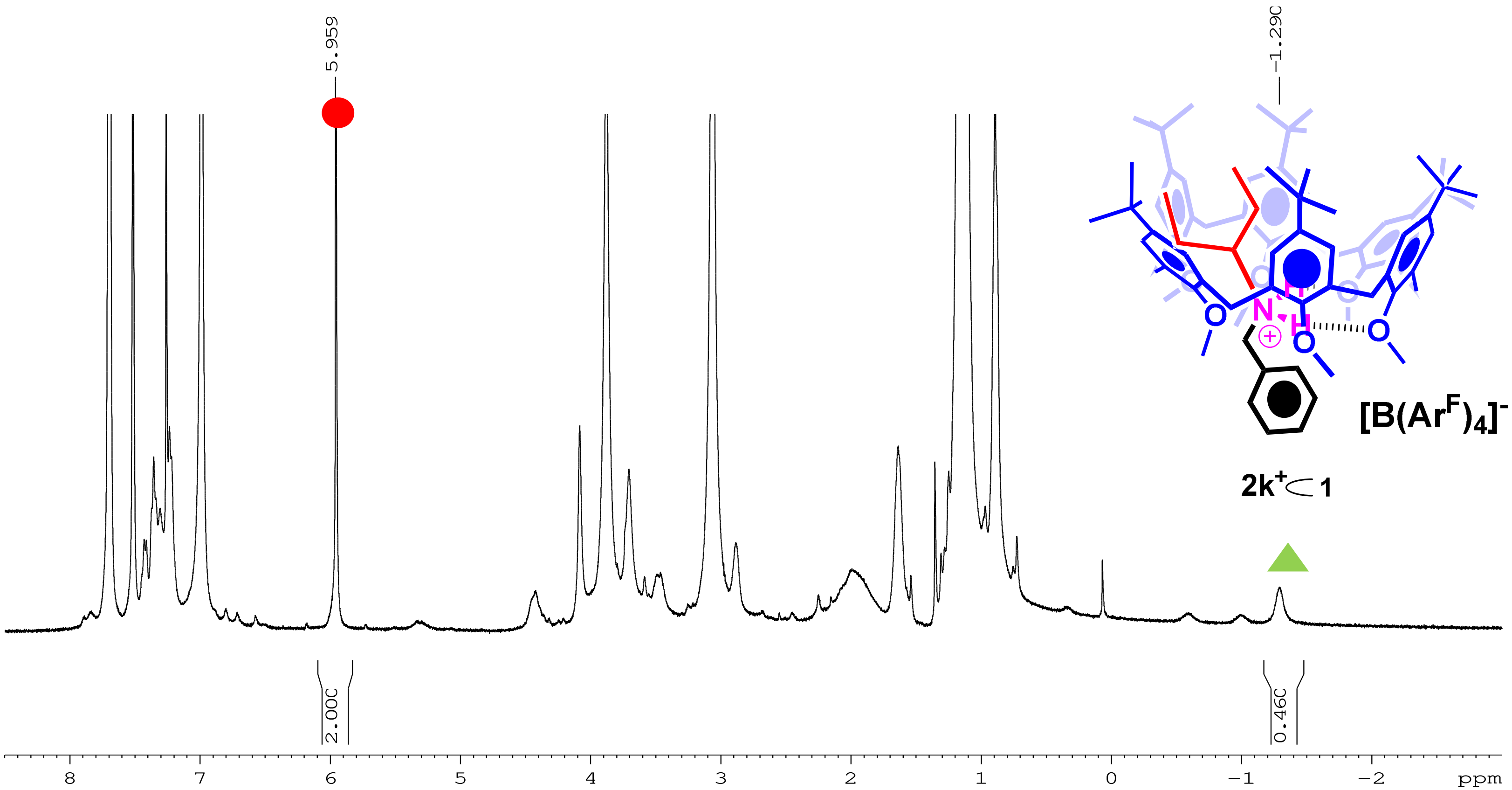

Figure S44. ^{1}H NMR spectrum ($CDCl_3$, 400 MHz, 298 K) of equimolar solution ($3.8 \cdot 10^{-3}$ M) of **1** and **2k$^+$⊂ 1**. The association constant $\boldsymbol{K_{ass}}$ value was calculated by integration of the complexed derivative **2k$^+$⊂ 1** (▲) and the signal of $CHCl_2CHCl_2$ (●).

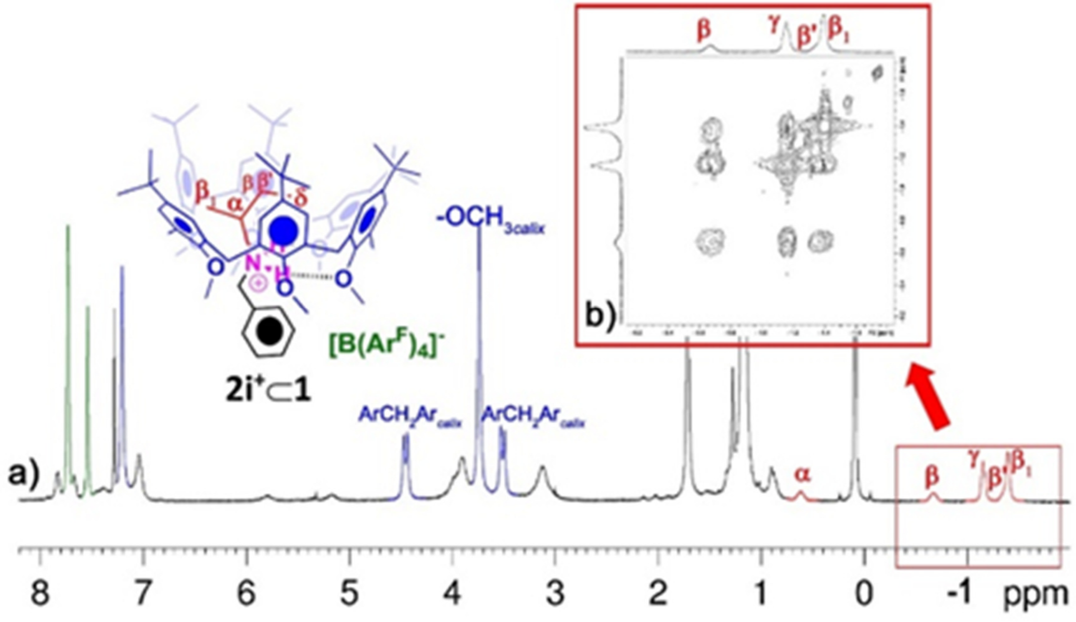

Figure S45. a) ^{1}H NMR and b) portion of 2D COSY spectra ($CDCl_3$, 400 MHz, 298 K) of pseudo[2]rotaxane **2i⁺⊂1** obtained by mixing **1** with 1 equivalent of $\mathbf{2i^+{\cdot}[B(Ar^F)_4]^-}$ (3.8 mM each one).

Computational details

All the screening procedures for the search of the most stable conformers have been made employing CREST software[3] which makes use of the xTB program[4]. All calculations executed with xTB were performed using the 6.3.2 release version of the program at GFN2-xTB level of theory, whereas the CREST version was the 2.10.2 with all parameters set to the default values. DFT calculations were performed with the Gaussian16 suite of programs[5] at the B3LYP-D3/6-31G(d,p) level of theory[6] with full geometry optimization. All stationary points were characterized by all real frequencies. Thermodynamic corrections were calculated at 298.15 K and 1 atm for the optimized geometries. Natural Bond Orbital (NBO) analyses were obtained with NBO 3.1 version implemented in Gaussian 16.

The *molecular surfaces* of the guest inside the cavity (S_{Guest}) were computed by YASARA software,[7] which also permits the direct measure of the *contact surface* between guest and host ($S_{Contact}$). The values of $V_{complexed\_Host}$ and V_{free_Host} were measured with the Caver analyst software employing 1,000,000 of number of samples.[8] In all the cases a probe of 1.0 Å was used.

---

[3] Pracht, P., Bohle, F., Grimme, S. Automated exploration of the low-energy chemical space with fast quantum chemical methods. *Physical Chemistry Chemical Physics* **22**, 7169-7192 (2020).

[4] Bannwarth, C., Caldeweyher, E., Ehlert, S., Hansen, A., Pracht, P., Seibert, J.*, et al.* Extended tight-binding quantum chemistry methods. *WIREs Computational Molecular Science* **n/a**, e01493.

[5] Frisch, M. J., Trucks, G. W., Schlegel, H. B., Scuseria, G. E., Robb, M. A., Cheeseman, J. R.*, et al.* Gaussian 16 Revision C.03.

[6] a) Tirado-Rives, J., Jorgensen, W. L. Performance of B3LYP density functional methods for a large set of organic molecules. *J. Chem. Theory Comput.* **4**, 297-306 (2008); b) Tirado-Rives, J., Jorgensen, W. L. Performance of B3LYP density functional methods for a large set of organic molecules. *J. Chem. Theory Comput.* **4**, 297-306 (2008).

[7] Krieger, E., Vriend, G. YASARA View—Molecular graphics for all devices—From smartphones to workstations. *Bioinform*. **30**, 2981–2982 (2014).

[8] Jurcik, A., Bednar, D., Byska, J., Marques, S. M., Furmanova, K., Daniel, L., Kokkonen, P., Brezovsky, J., Strnad, O., Stourac, J., Pavelka, A., Manak, M., Damborsky, J., Kozlikova, B. CAVER Analyst 2.0: Analysis and Visualization of Channels and Tunnels in Protein Structures and Molecular Dynamics Trajectories. *Bioinform*, **34**, 3586–3588, (2018).

Table S1. Superficies, volumes, and Gibbs free energies for the considered compounds.

2⊂1	Sup. Host	Sup. Guest	Sup. Contact G-H	Sup. H min	Vol. Host	Vol. Guest	*G* dist.
**2a⁺**	896.59	166.38	116.81	**808.94**	125.9	90.8	19.2
**2b⁺**	913.19	182.44	130.99		138	138	28.0
**2c⁺**	925.19	198.88	146.80		172.5	151.9	28.8
**2d⁺**	925.44	236.73	167.49		188.7	178.3	32.0
**2e⁺**	925.46	181.41	128.77		173.5	127.6	25.5
**2f⁺**	941.51	194.39	150.02		200.6	165	36.9
**2g⁺**	930.99	195.28	144.90		189.9	166.5	37.6
**2h⁺**	962.60	211.96	159.80		213.1	184.2	31.9
**2i⁺**	931.28	197.22	147.58		177.5	157.3	31.6
**2j⁺**	950.41	210.74	161.02		209.7	181.6	35.3
**2k⁺**	957.85	211.09	169.39		221	215.2	41.1

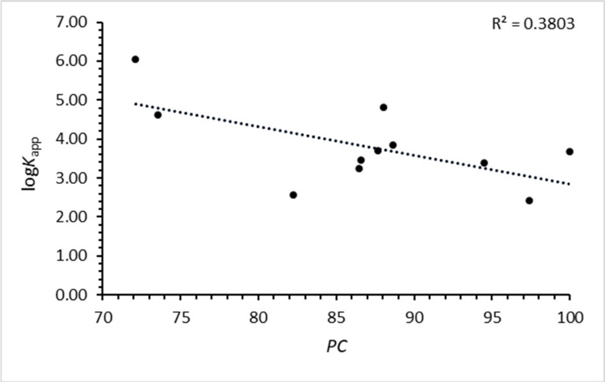

Figure S46. Linear fitting between the logK_{app} of pseudo[2]rotaxanes **2a–k⁺⊂1** and their packing coefficient (*PC*).

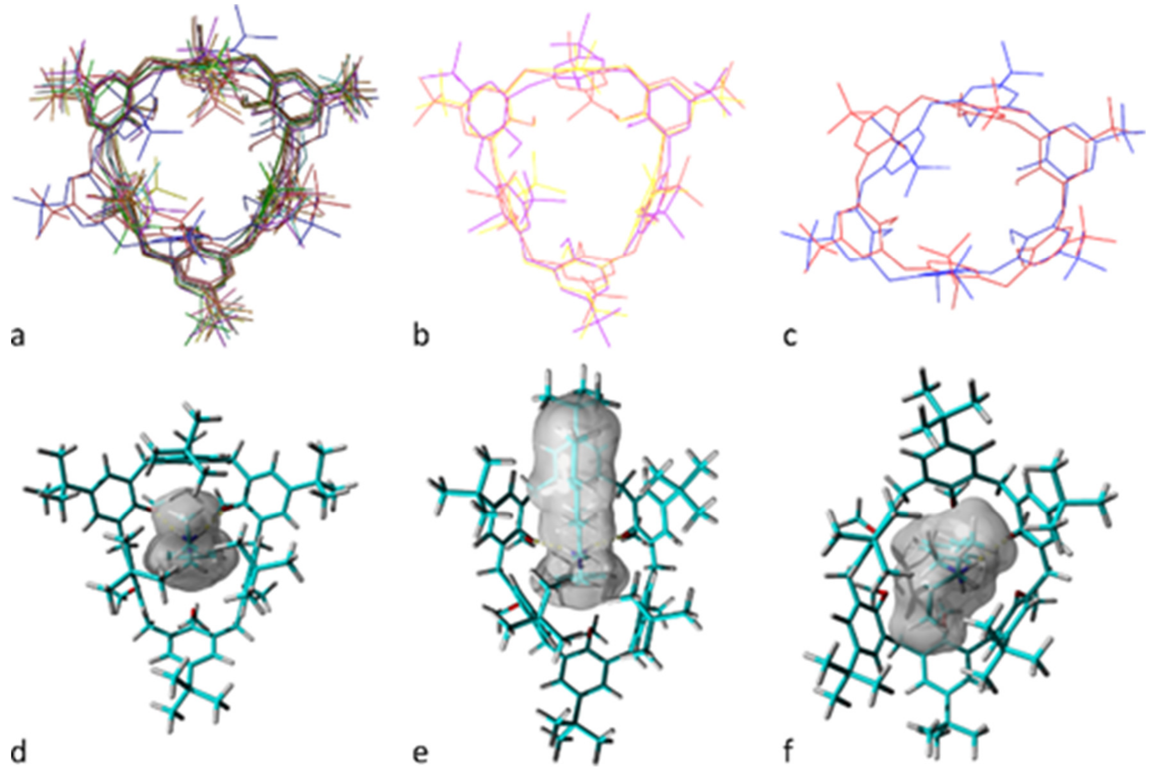

Figure S47. DFT-optimized structures, at B3LYP-D3/6-31G(d,p) level of theory, of: (a) superimposed calix[6]arene-wheels of **2a**$^{+}\subset$**1** (yellow), **2b**$^{+}\subset$**1** (coral), **2c**$^{+}\subset$**1** (orange), **2d**$^{+}\subset$**1** (blu), **2e**$^{+}\subset$**1** (light grey), **2f**$^{+}\subset$**1** (cyan), **2g**$^{+}\subset$**1** (green), **2h**$^{+}\subset$**1** (light orange), **2i**$^{+}\subset$**1** (pink), **2j**$^{+}\subset$**1** (purple), and **2k**$^{+}\subset$**1** (red) pseudorotaxanes (global minimum); (b) only **2a**$^{+}\subset$**1**, **2b**$^{+}\subset$**1**, and **2j**$^{+}\subset$**1**; (c) only **2d**$^{+}\subset$**1** and **2k**$^{+}\subset$**1**; (d) **2a**$^{+}\subset$**1** pseudorotaxane (top view); (e) **2d**$^{+}\subset$**1** pseudorotaxane (top view); (f) **2k**$^{+}\subset$**1** pseudorotaxane (top view).

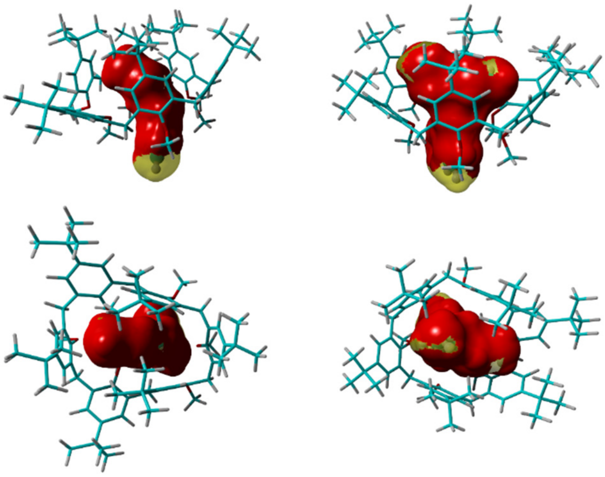

Figure S48. Representation of the $S_{contact}$ (in red) and the free molecular surface of the guest not in contact with the calixarene cavity (in yellow) for **2a**$^{+}\subset$**1** (left) and **2k**$^{+}$□**1** (right) pseudo[2]rotaxanes. The representations were built by the YASARA software starting from DFT-optimized structures of the pseudorotaxane complexes at the B3LYP-D3/6-31G(d,p) level of theory.

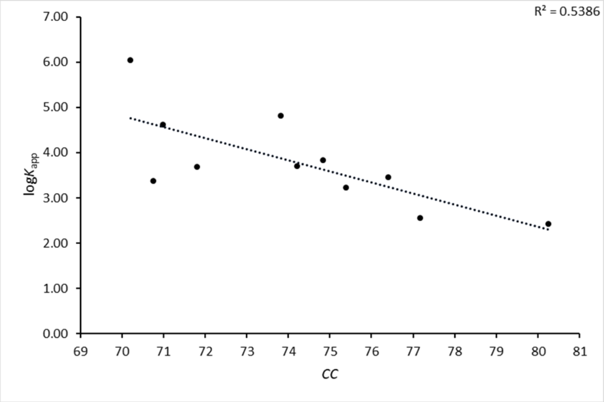

Figure S49. *CC* as a function of logK_{app} values for alkylbenzylammonium-based pseudorotaxane complexes of **1**.

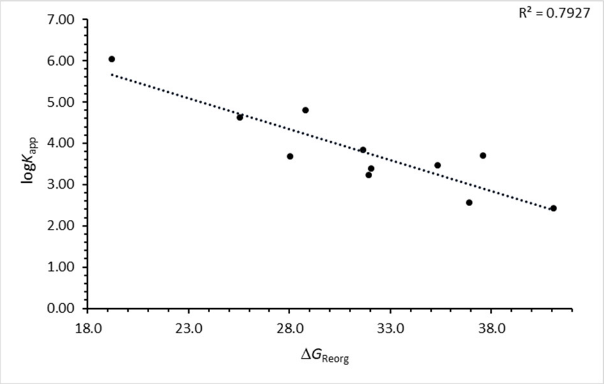

Figure S50. Linear regression analysis of ΔG_{Reorg} *vs.* logK_{app} values for $\mathbf{2}^{+}\subset\mathbf{1}$ alkylbenzylammonium-based pseudorotaxane complexes.

Table S2. Gibbs free energies, expressed in Hartree, and number of negative frequencies.

Structure	*G*	N.F.	Structure	*G*	N.F.
Host	–3251.812888	0			
**2a**$^{+}$	–405.775861	0	**2a**$^{+}$_Guest_SP	–405.770014	—
**2b**$^{+}$	–445.070188	0	**2b**$^{+}$_Guest_SP	–445.061061	—
**2c**$^{+}$	–484.363920	0	**2c**$^{+}$_Guest_SP	–484.355926	—
**2d**$^{+}$	–562.950600	0	**2d**$^{+}$_Guest_SP	–562.945490	—
**2e**$^{+}$	–445.075164	0	**2e**$^{+}$_Guest_SP	–445.066086	—
**2f**$^{+}$	–484.373556	0	**2f**$^{+}$_Guest_SP	–484.362094	—
**2g**$^{+}$	–484.366327	0	**2g**$^{+}$_Guest_SP	–484.350552	—
**2h**$^{+}$	–523.662452	0	**2h**$^{+}$_Guest_SP	–523.651513	—
**2i**$^{+}$	–484.369861	0	**2i**$^{+}$_Guest_SP	–484.356843	—
**2j**$^{+}$	–523.664144	0	**2j**$^{+}$_Guest_SP	–523.650827	—
**2k**$^{+}$	–523.663092	0	**2k**$^{+}$_Guest_SP	–523.647490	—

Structure	*G*	N.F.	Structure	*G*	N.F.
**2a**$^{+}$_Host_SP	–3251.788135	—	**2a**$^{+}$⊂**1**	–3657.669410	0
**2b**$^{+}$_Host_SP	–3251.777381	—	**2b**$^{+}$⊂**1**	–3696.957925	0
**2c**$^{+}$_Host_SP	–3251.774997	—	**2c**$^{+}$⊂**1**	–3736.253637	0
**2d**$^{+}$_Host_SP	–3251.766938	—	**2d**$^{+}$⊂**1**	–3814.842590	0
**2e**$^{+}$_Host_SP	–3251.781299	—	**2e**$^{+}$⊂**1**	–3696.960711	0
**2f**$^{+}$_Host_SP	–3251.765533	—	**2f**$^{+}$⊂**1**	–3736.257004	0
**2g**$^{+}$_Host_SP	–3251.768792	—	**2g**$^{+}$⊂**1**	–3736.253200	0
**2h**$^{+}$_Host_SP	–3251.772963	—	**2h**$^{+}$⊂**1**	–3775.541904	0
**2i**$^{+}$_Host_SP	–3251.775508	—	**2i**$^{+}$⊂**1**	–3736.256745	0
**2j**$^{+}$_Host_SP	–3251.769918	—	**2j**$^{+}$⊂**1**	–3775.546477	0
**2k**$^{+}$_Host_SP	–3251.763010	—	**2k**$^{+}$⊂**1**	–3775.547412	0

Cartesian coordinates of the full optimized structures for the complexes 2a–k^{+}⊂1

2a⊂1

```
---------------------------------------------------------------------
 Center     Atomic      Atomic             Coordinates (Angstroms)
 Number     Number       Type             X           Y           Z
 ---------------------------------------------------------------------
      1          8           0       -3.888494   -0.511854    2.064691
      2          6           0       -4.791615   -0.182396    1.064390
      3          6           0       -5.356860   -1.207071    0.290562
      4          6           0       -6.203271   -0.859380   -0.761029
      5          6           0       -6.511509    0.476527   -1.067204
      6          6           0       -5.938221    1.464486   -0.262564
      7          6           0       -5.063223    1.159060    0.790220
      8          6           0       -4.354812    2.256840    1.550592
      9          6           0       -3.058738    2.724680    0.895182
     10          6           0       -2.945996    2.856653   -0.496654
     11          6           0       -1.791135    3.349131   -1.109698
     12          6           0       -0.712711    3.695331   -0.280267
     13          6           0       -0.771825    3.567647    1.107164
     14          6           0       -1.957725    3.080569    1.683667
```

15	8	0	-1.986004	2.878000	3.059134
16	6	0	-2.536746	3.958741	3.820753
17	1	0	-2.458307	3.669399	4.870332
18	1	0	-1.978626	4.887968	3.655565
19	1	0	-3.589768	4.127600	3.567752
20	6	0	0.381984	3.958936	2.024114
21	6	0	1.700926	4.145806	1.319069
22	6	0	1.935788	5.322079	0.601533
23	6	0	3.097065	5.526965	-0.151007
24	6	0	4.059373	4.512256	-0.115783
25	6	0	3.885146	3.330043	0.613864
26	6	0	2.681299	3.145824	1.310216
27	8	0	2.433905	1.941903	1.984144
28	6	0	2.950096	1.892210	3.325682
29	1	0	2.531112	2.703306	3.932890
30	1	0	4.040047	1.953008	3.331155
31	1	0	2.647774	0.930056	3.740670
32	6	0	5.001103	2.307008	0.645599
33	6	0	4.711772	1.009070	-0.083036
34	6	0	4.443350	1.006985	-1.452348
35	6	0	4.237101	-0.177740	-2.176073
36	6	0	4.288232	-1.379274	-1.461045
37	6	0	4.555234	-1.420010	-0.086646
38	6	0	4.763645	-0.216911	0.594468
39	8	0	5.021504	-0.230331	1.962544
40	6	0	6.410153	-0.365633	2.291006
41	1	0	6.997142	0.465803	1.882274
42	1	0	6.821383	-1.307388	1.908443
43	1	0	6.476684	-0.356842	3.380900
44	6	0	4.671763	-2.752561	0.632254
45	6	0	3.499434	-3.678917	0.402263
46	6	0	3.634645	-4.774872	-0.456648
47	6	0	2.579285	-5.653952	-0.726986
48	6	0	1.373254	-5.418417	-0.059426

49	6	0	1.189293	-4.335325	0.804800
50	6	0	2.254953	-3.451911	1.007498
51	8	0	2.045454	-2.313655	1.789907
52	6	0	2.610990	-2.309967	3.122616
53	1	0	3.013115	-3.297015	3.364902
54	1	0	3.404752	-1.560409	3.166829
55	1	0	1.814057	-2.065835	3.829447
56	6	0	-0.161303	-4.104397	1.431887
57	6	0	-1.202716	-3.552010	0.466322
58	6	0	-0.880361	-3.183008	-0.843993
59	6	0	-1.836523	-2.664212	-1.726135
60	6	0	-3.145013	-2.527120	-1.250909
61	6	0	-3.509963	-2.875115	0.051996
62	6	0	-2.524568	-3.388141	0.904548
63	8	0	-2.825509	-3.687451	2.228440
64	6	0	-3.360740	-5.000785	2.431194
65	1	0	-2.666551	-5.775164	2.082796
66	1	0	-3.518682	-5.111353	3.505642
67	1	0	-4.316255	-5.125676	1.908163
68	6	0	-4.936239	-2.642626	0.525152
69	1	0	-5.007089	-2.897004	1.582964
70	1	0	-5.615767	-3.310872	-0.015627
71	1	0	-3.912867	-2.110340	-1.894937
72	6	0	-1.489934	-2.228466	-3.158856
73	6	0	-2.255507	-3.118804	-4.163434
74	1	0	-1.966417	-4.168592	-4.051148
75	1	0	-3.337389	-3.050542	-4.016194
76	1	0	-2.036250	-2.811902	-5.191825
77	6	0	0.017908	-2.344577	-3.454253
78	1	0	0.223744	-2.000930	-4.472458
79	1	0	0.369466	-3.378817	-3.378042
80	1	0	0.615872	-1.732884	-2.771078
81	6	0	-1.912909	-0.753662	-3.355415
82	1	0	-2.993766	-0.620296	-3.253658

83	1	0	-1.432982	-0.108144	-2.613852
84	1	0	-1.627217	-0.400006	-4.351665
85	1	0	0.143672	-3.306080	-1.167812
86	1	0	-0.064955	-3.427516	2.283305
87	1	0	-0.526942	-5.049952	1.847183
88	1	0	0.524573	-6.074705	-0.228329
89	6	0	2.685123	-6.808096	-1.735829
90	6	0	4.097221	-6.933543	-2.335726
91	1	0	4.389625	-6.030037	-2.880900
92	1	0	4.849396	-7.131006	-1.565032
93	1	0	4.123928	-7.766720	-3.044164
94	6	0	1.688697	-6.541525	-2.890034
95	1	0	1.924744	-5.599054	-3.396356
96	1	0	0.657498	-6.479641	-2.529387
97	1	0	1.737283	-7.346984	-3.630494
98	6	0	2.330616	-8.144244	-1.044683
99	1	0	1.314189	-8.139759	-0.640708
100	1	0	2.397906	-8.970051	-1.760547
101	1	0	3.019638	-8.351372	-0.219774
102	1	0	4.600343	-4.925030	-0.925417
103	1	0	4.806342	-2.571516	1.698517
104	1	0	5.581634	-3.258077	0.288290
105	1	0	4.124769	-2.324536	-1.965234
106	6	0	4.020219	-0.122905	-3.699187
107	6	0	3.802004	-1.520667	-4.308036
108	1	0	3.636904	-1.431124	-5.386061
109	1	0	2.925875	-2.017964	-3.879284
110	1	0	4.672288	-2.168255	-4.161512
111	6	0	5.275454	0.502180	-4.353147
112	1	0	5.444518	1.526129	-4.007142
113	1	0	5.163086	0.531718	-5.442220
114	1	0	6.169648	-0.083437	-4.117656
115	6	0	2.786521	0.744217	-4.041293
116	1	0	2.845344	1.742106	-3.597320

117	1	0	2.701667	0.866823	-5.126260
118	1	0	1.866438	0.270564	-3.688517
119	1	0	4.406305	1.966199	-1.961322
120	1	0	5.259712	2.080372	1.680072
121	1	0	5.890828	2.764635	0.199463
122	1	0	4.989977	4.629645	-0.658750
123	6	0	3.259615	6.813832	-0.975105
124	6	0	3.216535	8.038597	-0.032811
125	1	0	2.273539	8.095464	0.518803
126	1	0	3.320758	8.964011	-0.608849
127	1	0	4.031188	7.997817	0.697087
128	6	0	2.098050	6.906003	-1.993499
129	1	0	2.093695	6.038100	-2.661682
130	1	0	2.199902	7.807154	-2.607096
131	1	0	1.124570	6.953261	-1.496377
132	6	0	4.587179	6.845847	-1.753560
133	1	0	4.664172	6.012829	-2.460192
134	1	0	4.654020	7.773476	-2.329510
135	1	0	5.452233	6.812072	-1.083458
136	1	0	1.162202	6.084216	0.618305
137	1	0	0.466560	3.211546	2.814321
138	1	0	0.119992	4.895542	2.532499
139	1	0	0.198159	4.075812	-0.724404
140	6	0	-1.688942	3.582866	-2.627327
141	6	0	-0.415516	2.918325	-3.198863
142	1	0	-0.456023	1.832152	-3.086436
143	1	0	0.495443	3.275535	-2.710190
144	1	0	-0.323908	3.138747	-4.267501
145	6	0	-2.906009	3.019765	-3.385677
146	1	0	-3.835040	3.519691	-3.093261
147	1	0	-2.776087	3.175404	-4.460820
148	1	0	-3.026394	1.945066	-3.215262
149	6	0	-1.612830	5.108151	-2.877627
150	1	0	-1.556978	5.316859	-3.951469

151	1	0	-0.729186	5.546546	-2.404106
152	1	0	-2.496676	5.614136	-2.476547
153	1	0	-3.792478	2.559589	-1.100509
154	1	0	-4.122782	1.928500	2.563872
155	1	0	-5.026594	3.120297	1.639400
156	1	0	-6.147365	2.510980	-0.453491
157	6	0	-7.397390	0.797971	-2.281279
158	6	0	-7.640867	2.309338	-2.444638
159	1	0	-8.143929	2.736382	-1.571079
160	1	0	-8.280235	2.487845	-3.314271
161	1	0	-6.706121	2.856779	-2.607172
162	6	0	-6.690012	0.283684	-3.558302
163	1	0	-7.293706	0.504635	-4.445027
164	1	0	-5.713810	0.765764	-3.681606
165	1	0	-6.529662	-0.798216	-3.526173
166	6	0	-8.767578	0.098937	-2.131056
167	1	0	-8.666661	-0.987736	-2.058392
168	1	0	-9.401397	0.315419	-2.997450
169	1	0	-9.286547	0.448511	-1.232981
170	1	0	-6.619681	-1.659264	-1.366619
171	6	0	-4.472210	-0.759043	3.348018
172	1	0	-3.651855	-1.036422	4.011545
173	1	0	-5.199119	-1.578119	3.307607
174	1	0	-4.973086	0.137078	3.734637
175	6	0	-0.100639	0.168811	0.050295
176	6	0	1.147716	0.401340	-0.775406
177	1	0	1.836925	-0.444067	-0.713901
178	1	0	1.680861	1.300225	-0.461346
179	1	0	0.865097	0.533223	-1.818997
180	7	0	0.261785	-0.074380	1.490848
181	1	0	0.868233	-0.918187	1.548882
182	1	0	0.848427	0.714740	1.811686
183	6	0	-0.963349	-0.258333	2.356448
184	6	0	-0.607319	-0.533688	3.790659

185	6	0	-0.867056	-1.786324	4.361327
186	6	0	-0.500143	-2.043528	5.684884
187	6	0	0.127257	-1.054779	6.444602
188	6	0	0.358820	0.206892	5.889133
189	6	0	-0.018904	0.468975	4.572987
190	1	0	0.111061	1.462795	4.155935
191	1	0	0.818321	0.988489	6.486720
192	1	0	0.417372	-1.258184	7.470772
193	1	0	-0.705796	-3.016247	6.121178
194	1	0	-1.375401	-2.547136	3.775661
195	1	0	-1.556429	0.648343	2.259084
196	1	0	-1.534854	-1.073168	1.915521
197	1	0	-0.651594	-0.712700	-0.279315
198	1	0	-0.771488	1.027798	0.031482

2b⊂1

Center Number	Atomic Number	Atomic Type	Coordinates (Angstroms) X	Y	Z
1	8	0	2.635937	-2.167088	1.579011
2	6	0	3.836937	-2.176138	0.865011
3	6	0	4.681983	-1.069173	0.973011
4	6	0	5.884983	-1.063223	0.249011
5	6	0	6.248940	-2.101238	-0.605989
6	6	0	5.367895	-3.191201	-0.688989
7	6	0	4.176892	-3.259152	0.033011
8	6	0	3.260843	-4.449114	-0.140989
9	6	0	1.910857	-4.098058	-0.739989
10	6	0	1.821895	-3.201054	-1.807989
11	6	0	0.600908	-2.889004	-2.420989
12	6	0	-0.550118	-3.506956	-1.913989
13	6	0	-0.502156	-4.410958	-0.850989

14	6	0	0.737832	-4.698009	-0.267989
15	8	0	0.797797	-5.557012	0.826011
16	6	0	1.023740	-6.931021	0.485011
17	1	0	1.977735	-7.057061	-0.040989
18	1	0	1.049717	-7.487022	1.424011
19	1	0	0.215724	-7.317988	-0.147989
20	6	0	-1.761185	-5.109906	-0.378989
21	6	0	-2.959147	-4.210856	-0.162989
22	6	0	-4.157159	-4.493806	-0.818989
23	6	0	-5.327128	-3.739758	-0.631989
24	6	0	-5.250083	-2.670761	0.258011
25	6	0	-4.057069	-2.316811	0.908011
26	6	0	-2.917101	-3.094858	0.694011
27	8	0	-1.725086	-2.731907	1.331011
28	6	0	-1.385119	-3.526921	2.485011
29	1	0	-0.595097	-2.991954	3.011011
30	1	0	-2.250123	-3.625885	3.149011
31	1	0	-1.013160	-4.510937	2.191011
32	6	0	-4.025017	-1.075812	1.763011
33	6	0	-4.004963	0.233187	0.978011
34	6	0	-4.322960	0.301201	-0.376989
35	6	0	-4.324910	1.509201	-1.091989
36	6	0	-3.969862	2.665186	-0.398989
37	6	0	-3.628863	2.646172	0.961011
38	6	0	-3.657914	1.425173	1.640011
39	8	0	-3.269916	1.358157	2.975011
40	6	0	-4.330909	1.537201	3.919011
41	1	0	-5.119940	0.788234	3.785011
42	1	0	-4.774868	2.536219	3.831011
43	1	0	-3.886914	1.423182	4.910011
44	6	0	-3.236809	3.940155	1.657011
45	6	0	-1.988783	4.582104	1.078011
46	6	0	-2.078746	5.473107	0.007011
47	6	0	-0.949722	6.054061	-0.587989

48	6	0	0.293266	5.755009	-0.023989
49	6	0	0.434229	4.866003	1.049011
50	6	0	-0.715796	4.266051	1.576011
51	8	0	-0.589836	3.289046	2.552011
52	6	0	-0.656818	3.729048	3.910011
53	1	0	-1.609798	4.224088	4.128011
54	1	0	-0.564855	2.832045	4.525011
55	1	0	0.158211	4.422015	4.149011
56	6	0	1.805216	4.552946	1.618011
57	6	0	2.550170	3.433915	0.906011
58	6	0	2.806173	3.506905	-0.465989
59	6	0	3.526132	2.520875	-1.150989
60	6	0	3.973086	1.419856	-0.411989
61	6	0	3.760082	1.308865	0.966011
62	6	0	3.049124	2.330895	1.614011
63	8	0	2.790119	2.207906	2.976011
64	6	0	3.771144	2.805865	3.831011
65	1	0	3.453135	2.603878	4.856011
66	1	0	3.827189	3.888863	3.673011
67	1	0	4.764126	2.372824	3.666011
68	6	0	4.305033	0.141843	1.787011
69	1	0	3.570022	-0.133127	2.543011
70	1	0	5.191048	0.486806	2.335011
71	1	0	4.511053	0.624834	-0.908989
72	6	0	3.857139	2.704861	-2.642989
73	6	0	2.562148	2.903915	-3.461989
74	1	0	2.806155	3.072905	-4.515989
75	1	0	1.985183	3.765939	-3.114989
76	1	0	1.919111	2.022942	-3.398989
77	6	0	4.617089	1.496830	-3.223989
78	1	0	4.040051	0.571854	-3.134989
79	1	0	5.583083	1.347790	-2.728989
80	1	0	4.816096	1.662822	-4.286989
81	6	0	4.751191	3.958824	-2.794989

82	1	0	5.675187	3.851786	-2.218989
83	1	0	5.019198	4.109813	-3.845989
84	1	0	4.241229	4.861845	-2.446989
85	1	0	2.432208	4.367920	-1.008989
86	1	0	2.416254	5.461921	1.564011
87	1	0	1.714205	4.289950	2.670011
88	1	0	1.190285	6.222972	-0.412989
89	6	0	-1.115684	6.958068	-1.819989
90	6	0	-2.010634	8.168105	-1.466989
91	1	0	-3.004647	7.857146	-1.131989
92	1	0	-1.559609	8.766086	-0.667989
93	1	0	-2.141607	8.812110	-2.342989
94	6	0	0.232338	7.488012	-2.341989
95	1	0	0.901304	6.671984	-2.637989
96	1	0	0.066364	8.114018	-3.223989
97	1	0	0.745363	8.101990	-1.594989
98	6	0	-1.780718	6.135095	-2.949989
99	1	0	-1.898693	6.748100	-3.849989
100	1	0	-2.771733	5.775136	-2.661989
101	1	0	-1.167754	5.264070	-3.206989
102	1	0	-3.066736	5.718148	-0.370989
103	1	0	-4.065780	4.650190	1.567011
104	1	0	-3.095818	3.741150	2.718011
105	1	0	-3.940823	3.618185	-0.910989
106	6	0	-4.713910	1.507217	-2.579989
107	6	0	-4.681852	2.919215	-3.192989
108	1	0	-5.383824	3.594245	-2.691989
109	1	0	-4.967854	2.870227	-4.247989
110	1	0	-3.681833	3.361174	-3.142989
111	6	0	-6.144934	0.938276	-2.730989
112	1	0	-6.443934	0.935288	-3.783989
113	1	0	-6.866909	1.543306	-2.172989
114	1	0	-6.212976	-0.090721	-2.362989
115	6	0	-3.725947	0.616176	-3.366989

116	1	0	-4.004949	0.581187	-4.424989
117	1	0	-2.707931	1.011134	-3.296989
118	1	0	-3.713990	-0.410825	-2.990989
119	1	0	-4.572998	-0.615789	-0.894989
120	1	0	-4.904017	-1.077775	2.420011
121	1	0	-3.157018	-1.099848	2.422011
122	1	0	-6.120058	-2.052725	0.448011
123	6	0	-6.600142	-4.083705	-1.419989
124	6	0	-7.015203	-5.543688	-1.131989
125	1	0	-6.236232	-6.255720	-1.419989
126	1	0	-7.920213	-5.798650	-1.694989
127	1	0	-7.225209	-5.687679	-0.066989
128	6	0	-6.308135	-3.915717	-2.929989
129	1	0	-7.201145	-4.152680	-3.518989
130	1	0	-5.503162	-4.576751	-3.264989
131	1	0	-6.014092	-2.884729	-3.157989
132	6	0	-7.779104	-3.163656	-1.052989
133	1	0	-7.563060	-2.113665	-1.278989
134	1	0	-8.662116	-3.448620	-1.632989
135	1	0	-8.041107	-3.241645	0.007011
136	1	0	-4.172194	-5.340806	-1.497989
137	1	0	-2.036216	-5.868894	-1.120989
138	1	0	-1.541207	-5.660915	0.539011
139	1	0	-1.521109	-3.295916	-2.348989
140	6	0	0.534945	-1.977001	-3.656989
141	6	0	1.669989	-0.930048	-3.652989
142	1	0	1.552017	-0.248043	-4.499989
143	1	0	1.672014	-0.333048	-2.735989
144	1	0	2.655970	-1.392089	-3.751989
145	6	0	0.683909	-2.861007	-4.916989
146	1	0	1.643887	-3.388047	-4.913989
147	1	0	0.632934	-2.250005	-5.824989
148	1	0	-0.112122	-3.610974	-4.963989
149	6	0	-0.815024	-1.233945	-3.723989

150	1	0	-1.648052	-1.909910	-3.931989
151	1	0	-0.799993	-0.490946	-4.527989
152	1	0	-1.035002	-0.715936	-2.786989
153	1	0	2.740914	-2.744092	-2.156989
154	1	0	3.759813	-5.172135	-0.797989
155	1	0	3.109821	-4.967108	0.808011
156	1	0	5.606861	-4.022211	-1.345989
157	6	0	7.517942	-2.069291	-1.470989
158	6	0	8.362994	-0.807326	-1.220989
159	1	0	7.811032	0.106697	-1.466989
160	1	0	8.694997	-0.740339	-0.179989
161	1	0	9.255993	-0.830363	-1.851989
162	6	0	7.101941	-2.085273	-2.961989
163	1	0	7.988942	-2.057310	-3.603989
164	1	0	6.479977	-1.216247	-3.200989
165	1	0	6.534904	-2.985250	-3.215989
166	6	0	8.390890	-3.307327	-1.163989
167	1	0	8.696890	-3.318339	-0.112989
168	1	0	7.861851	-4.242305	-1.368989
169	1	0	9.294891	-3.296364	-1.781989
170	1	0	6.524019	-0.194249	0.357011
171	6	0	2.506897	-3.142083	2.639011
172	1	0	3.495883	-3.470124	2.973011
173	1	0	1.995917	-2.650062	3.468011
174	1	0	1.922861	-4.000058	2.299011
175	6	0	0.411034	0.151004	-0.011989
176	7	0	0.223004	-0.557988	1.315011
177	1	0	1.084981	-1.111024	1.479011
178	1	0	-0.555024	-1.239956	1.241011
179	6	0	-0.021956	0.397022	2.472011
180	6	0	0.081017	-0.257982	3.823011
181	6	0	1.319013	-0.357033	4.473011
182	6	0	1.412987	-0.966037	5.726011
183	6	0	0.267966	-1.469990	6.347011

184	6	0	-0.974028	-1.339938	5.724011
185	6	0	-1.068003	-0.729934	4.472011
186	1	0	-2.034997	-0.583894	4.004011
187	1	0	-1.871043	-1.702901	6.216011
188	1	0	0.341947	-1.942993	7.322011
189	1	0	2.376984	-1.036077	6.221011
190	1	0	2.195031	0.079930	4.008011
191	1	0	-1.010939	0.824063	2.333011
192	1	0	0.704077	1.199992	2.369011
193	6	0	-0.763929	1.049053	-0.380989
194	6	0	-0.450892	1.939040	-1.584989
195	1	0	-0.213916	1.359030	-2.480989
196	1	0	0.396135	2.595005	-1.369989
197	1	0	-1.314866	2.571076	-1.806989
198	1	0	-1.651955	0.438090	-0.572989
199	1	0	-1.009902	1.705063	0.455011
200	1	0	0.570001	-0.643002	-0.746989
201	1	0	1.335057	0.724966	0.082011

2c⊂1

Center Number	Atomic Number	Atomic Type	Coordinates (Angstroms) X	Y	Z
1	6	0	-0.500628	4.286945	1.572000
2	6	0	0.756303	4.781121	1.207000
3	6	0	0.822163	5.780130	0.226000
4	6	0	-0.316907	6.275971	-0.414000
5	6	0	-1.557837	5.776797	0.010000
6	6	0	-1.673700	4.797781	0.997000
7	6	0	-3.035627	4.273590	1.412000
8	6	0	-3.418445	2.978537	0.716000
9	6	0	-3.646445	2.979505	-0.666000

10	6	0	-3.996284	1.823456	-1.364000
11	6	0	-4.097117	0.629442	-0.628000
12	6	0	-3.903110	0.585469	0.752000
13	6	0	-3.565278	1.779516	1.416000
14	8	0	-3.341272	1.737547	2.790000
15	6	0	-4.494312	2.023386	3.589000
16	1	0	-5.312213	1.321272	3.386000
17	1	0	-4.182298	1.923430	4.631000
18	1	0	-4.857454	3.042335	3.414000
19	6	0	-4.078933	-0.685556	1.580000
20	6	0	-4.222754	-1.961576	0.790000
21	6	0	-5.411717	-2.226742	0.103000
22	6	0	-5.590557	-3.367767	-0.685000
23	6	0	-4.520431	-4.267618	-0.749000
24	6	0	-3.320460	-4.062450	-0.058000
25	6	0	-3.182623	-2.896430	0.709000
26	8	0	-1.993660	-2.634264	1.396000
27	6	0	-1.809557	-3.369238	2.625000
28	1	0	-1.058633	-2.827133	3.203000
29	1	0	-1.446416	-4.379187	2.425000
30	1	0	-2.742553	-3.395369	3.196000
31	6	0	-2.214316	-5.090295	-0.154000
32	6	0	-0.896392	-4.551110	-0.674000
33	6	0	-0.860500	-3.777105	-1.837000
34	6	0	0.338433	-3.297938	-2.379000
35	6	0	1.523478	-3.617772	-1.702000
36	6	0	1.529586	-4.387771	-0.537000
37	6	0	0.308650	-4.845942	-0.025000
38	8	0	0.292751	-5.568944	1.162000
39	6	0	0.415949	-6.985927	0.986000
40	1	0	0.389011	-7.428931	1.983000
41	1	0	1.363985	-7.243794	0.498000
42	1	0	-0.410995	-7.385043	0.386000
43	6	0	2.832638	-4.761589	0.144000

44	6	0	3.848481	-3.643447	0.175000
45	6	0	4.979488	-3.693288	-0.648000
46	6	0	5.940346	-2.676154	-0.661000
47	6	0	5.728194	-1.592183	0.198000
48	6	0	4.593178	-1.476342	1.007000
49	6	0	3.659324	-2.519473	0.988000
50	8	0	2.514314	-2.449633	1.794000
51	6	0	2.667407	-3.111612	3.062000
52	1	0	3.023550	-4.136562	2.928000
53	1	0	3.375331	-2.568513	3.700000
54	1	0	1.686409	-3.123749	3.537000
55	6	0	4.385004	-0.233371	1.837000
56	6	0	3.891834	0.985560	1.061000
57	6	0	4.169806	1.179598	-0.292000
58	6	0	3.766645	2.331542	-0.986000
59	6	0	3.037511	3.288440	-0.277000
60	6	0	2.731532	3.140397	1.082000
61	6	0	3.168694	1.983458	1.739000
62	8	0	2.832723	1.777411	3.074000
63	6	0	3.784654	2.270545	4.023000
64	1	0	3.391689	2.022490	5.011000
65	1	0	3.901502	3.357561	3.938000
66	1	0	4.764720	1.796682	3.889000
67	6	0	2.017378	4.244297	1.853000
68	1	0	2.721261	5.076396	1.979000
69	1	0	1.788430	3.867265	2.847000
70	1	0	2.690386	4.182391	-0.777000
71	6	0	4.205617	2.533604	-2.448000
72	6	0	3.538445	3.765510	-3.090000
73	1	0	3.827315	4.694551	-2.587000
74	1	0	2.446455	3.689357	-3.070000
75	1	0	3.850433	3.851554	-4.135000
76	6	0	3.859790	1.297555	-3.310000
77	1	0	4.241771	1.434609	-4.326000

78	1	0	2.778811	1.150404	-3.377000
79	1	0	4.300919	0.378617	-2.915000
80	6	0	5.739588	2.738818	-2.461000
81	1	0	6.261711	1.863891	-2.063000
82	1	0	6.094565	2.905868	-3.484000
83	1	0	6.022467	3.604858	-1.855000
84	1	0	4.722914	0.413676	-0.820000
85	1	0	3.682032	-0.431470	2.646000
86	1	0	5.336968	0.027762	2.318000
87	1	0	6.457082	-0.788081	0.236000
88	6	0	7.173349	-2.693981	-1.578000
89	6	0	7.231526	-3.958973	-2.454000
90	1	0	6.359537	-4.039095	-3.111000
91	1	0	7.293653	-4.868964	-1.849000
92	1	0	8.120521	-3.925849	-3.090000
93	6	0	7.118176	-1.460989	-2.512000
94	1	0	6.208178	-1.472116	-3.121000
95	1	0	7.134045	-0.522987	-1.948000
96	1	0	7.979175	-1.455869	-3.188000
97	6	0	8.458340	-2.634801	-0.721000
98	1	0	9.343341	-2.639678	-1.366000
99	1	0	8.520461	-3.498793	-0.051000
100	1	0	8.499214	-1.730796	-0.108000
101	1	0	5.096610	-4.562272	-1.286000
102	1	0	2.603686	-5.107621	1.153000
103	1	0	3.274758	-5.618527	-0.379000
104	1	0	2.477429	-3.266638	-2.077000
105	6	0	0.361324	-2.517934	-3.704000
106	6	0	-0.928792	-1.690115	-3.877000
107	1	0	-0.847883	-1.039104	-4.753000
108	1	0	-1.120880	-1.062142	-3.003000
109	1	0	-1.807703	-2.323238	-4.030000
110	6	0	1.569190	-1.559765	-3.770000
111	1	0	1.604097	-0.893761	-2.904000

112	1	0	2.521265	-2.096632	-3.817000
113	1	0	1.508103	-0.939774	-4.670000
114	6	0	0.466466	-3.533920	-4.865000
115	1	0	0.479393	-3.014918	-5.830000
116	1	0	-0.383438	-4.223039	-4.859000
117	1	0	1.381549	-4.127792	-4.783000
118	1	0	-1.806531	-3.554238	-2.319000
119	1	0	-2.052250	-5.560272	0.818000
120	1	0	-2.551204	-5.893342	-0.821000
121	1	0	-4.604305	-5.167629	-1.346000
122	6	0	-6.915527	-3.585953	-1.433000
123	6	0	-6.902348	-4.866951	-2.286000
124	1	0	-6.758222	-5.762931	-1.674000
125	1	0	-7.860333	-4.974085	-2.804000
126	1	0	-6.116351	-4.838841	-3.049000
127	6	0	-8.066512	-3.695114	-0.406000
128	1	0	-7.908393	-4.544092	0.267000
129	1	0	-8.152638	-2.792126	0.205000
130	1	0	-9.022491	-3.842248	-0.921000
131	6	0	-7.176695	-2.382989	-2.369000
132	1	0	-8.107675	-2.530120	-2.927000
133	1	0	-6.362712	-2.264875	-3.092000
134	1	0	-7.271826	-1.446003	-1.812000
135	1	0	-6.211819	-1.496854	0.186000
136	1	0	-3.238920	-0.773438	2.269000
137	1	0	-4.969949	-0.565680	2.209000
138	1	0	-4.351988	-0.289594	-1.142000
139	6	0	-4.302283	1.816413	-2.872000
140	6	0	-5.762217	1.348209	-3.084000
141	1	0	-6.464311	2.015110	-2.573000
142	1	0	-6.009217	1.344174	-4.151000
143	1	0	-5.920075	0.335187	-2.703000
144	6	0	-4.147478	3.209435	-3.510000
145	1	0	-3.126531	3.592578	-3.419000

146	1	0	-4.830580	3.938339	-3.062000
147	1	0	-4.382470	3.152402	-4.577000
148	6	0	-3.347146	0.839547	-3.593000
149	1	0	-3.570142	0.811515	-4.665000
150	1	0	-3.442003	-0.179467	-3.209000
151	1	0	-2.305189	1.149692	-3.471000
152	1	0	-3.538577	3.922520	-1.188000
153	1	0	-3.061606	4.123587	2.490000
154	1	0	-3.787733	5.034485	1.175000
155	1	0	-2.469890	6.159669	-0.438000
156	6	0	-0.257051	7.309979	-1.550000
157	6	0	-0.902966	6.698889	-2.816000
158	1	0	-0.369839	5.793963	-3.126000
159	1	0	-0.871066	7.415893	-3.644000
160	1	0	-1.949928	6.428742	-2.651000
161	6	0	1.187893	7.711181	-1.898000
162	1	0	1.181791	8.436180	-2.718000
163	1	0	1.781013	6.849264	-2.224000
164	1	0	1.696827	8.180253	-1.050000
165	6	0	-1.031229	8.582871	-1.137000
166	1	0	-1.002333	9.322875	-1.944000
167	1	0	-2.081199	8.367724	-0.921000
168	1	0	-0.590293	9.033932	-0.243000
169	1	0	1.801108	6.169267	-0.032000
170	8	0	-0.587481	3.230933	2.467000
171	6	0	-0.740530	3.580911	3.845000
172	1	0	-0.841398	2.640897	4.390000
173	1	0	-1.633615	4.192786	4.010000
174	1	0	0.132394	4.130034	4.217000
175	6	0	0.542002	-0.217909	-0.023000
176	7	0	0.147077	-0.756964	1.337000
177	1	0	-0.708841	-1.340084	1.255000
178	1	0	0.911167	-1.394858	1.623000
179	6	0	-0.064073	0.318006	2.387000

180	6	0	-0.142995	-0.238005	3.783000
181	6	0	1.027055	-0.598841	4.464000
182	6	0	0.963129	-1.126850	5.753000
183	6	0	-0.273849	-1.282023	6.384000
184	6	0	-1.441904	-0.888187	5.728000
185	6	0	-1.376977	-0.365178	4.435000
186	1	0	-2.276025	-0.022303	3.934000
187	1	0	-2.402891	-0.983321	6.224000
188	1	0	-0.324792	-1.689030	7.389000
189	1	0	1.876168	-1.404722	6.270000
190	1	0	1.986032	-0.432707	3.985000
191	1	0	-0.975147	0.849879	2.123000
192	1	0	0.766830	1.013122	2.298000
193	6	0	-0.269168	0.995977	-0.468000
194	1	0	-1.338135	0.757828	-0.512000
195	6	0	0.223761	1.503046	-1.831000
196	6	0	-0.285436	2.908975	-2.149000
197	1	0	-1.375442	2.955823	-2.094000
198	1	0	0.016521	3.220017	-3.155000
199	1	0	0.107462	3.638030	-1.435000
200	1	0	1.318759	1.518200	-1.824000
201	1	0	-0.074141	0.804005	-2.618000
202	1	0	-0.152281	1.803994	0.259000
203	1	0	0.445120	-1.058923	-0.714000
204	1	0	1.599965	0.043239	0.055000

2d⊂1

Center Number	Atomic Number	Atomic Type	Coordinates (Angstroms) X	Y	Z
1	6	0	0.437987	-0.049017	-0.020997
2	6	0	-1.194016	-0.204981	1.896003

3	6	0	-1.308029	-0.755978	6.161003
4	6	0	-1.333053	-1.863978	5.311003
5	6	0	-1.317049	-1.689978	3.926003
6	6	0	-1.276021	-0.399979	3.384003
7	6	0	-1.270996	0.711021	4.237003
8	6	0	-1.285000	0.533021	5.621003
9	7	0	0.256983	-0.222013	1.465003
10	6	0	1.911988	-0.002050	-0.390997
11	8	0	1.259919	-3.110035	2.137003
12	8	0	-2.539103	-4.075951	1.966003
13	8	0	-2.976942	3.140059	2.486003
14	8	0	-4.196007	0.201086	2.076003
15	8	0	1.276040	2.339964	2.851003
16	6	0	-5.199011	0.024109	1.133003
17	6	0	-2.355064	-2.360955	-1.864997
18	6	0	-2.487091	-3.533952	0.687003
19	6	0	-3.436059	-2.121931	-1.010997
20	6	0	-1.330083	-3.189978	-1.393997
21	6	0	-5.550040	-1.267884	0.726003
22	6	0	-5.338955	2.533112	0.945003
23	6	0	1.783057	3.067953	1.772003
24	6	0	-1.648933	3.525029	0.504003
25	6	0	-4.730066	-2.453902	1.170003
26	6	0	-2.881941	3.186057	1.098003
27	6	0	-6.559043	-1.414861	-0.228997
28	6	0	3.751910	-3.520091	-0.542997
29	6	0	-3.995948	2.872082	0.315003
30	6	0	1.127893	-4.280032	0.028003
31	6	0	3.006896	-4.144074	-1.544997
32	6	0	4.139040	2.314900	2.411003
33	6	0	-3.530072	-2.699929	0.259003
34	6	0	-0.283117	-4.731001	0.333003
35	6	0	-2.668939	3.242052	-1.709997
36	6	0	-0.528922	4.029004	1.405003

37	6	0	-3.869947	2.906079	-1.081997
38	6	0	-1.375096	-3.781977	-0.129997
39	6	0	1.434088	4.475961	-0.144997
40	6	0	3.225916	-3.254079	0.728003
41	6	0	3.612069	3.606912	0.308003
42	6	0	-5.813986	1.147122	0.570003
43	6	0	3.153055	2.994922	1.476003
44	6	0	2.770085	4.341931	-0.536997
45	6	0	0.910074	3.828972	0.982003
46	6	0	1.883909	-3.567049	0.972003
47	6	0	-1.563933	3.513028	-0.886997
48	6	0	-7.210019	-0.317846	-0.810997
49	6	0	-6.809990	0.957145	-0.395997
50	6	0	1.711886	-4.566046	-1.206997
51	6	0	4.125928	-2.710099	1.826003
52	6	0	5.963960	-1.257140	0.859003
53	6	0	6.583988	-0.028154	0.617003
54	6	0	5.972013	1.110859	1.157003
55	6	0	4.768012	1.053886	1.861003
56	6	0	4.154984	-0.193100	2.039003
57	6	0	4.757958	-1.361113	1.564003
58	8	0	2.892982	-0.265072	2.626003
59	6	0	1.280056	3.024964	4.113003
60	6	0	-3.156131	-5.365937	2.041003
61	6	0	-4.698006	0.242097	3.418003
62	6	0	1.324900	-3.980037	3.277003
63	6	0	2.853979	-0.410071	4.048003
64	6	0	-3.376914	4.371068	3.096003
65	6	0	-2.303052	-1.802956	-3.294997
66	6	0	-3.371028	-0.715932	-3.520997
67	6	0	-0.914038	-1.193987	-3.585997
68	6	0	-2.559078	-2.964950	-4.283997
69	6	0	3.550890	-4.396087	-2.958997
70	6	0	3.689856	-5.918090	-3.191997

71	6	0	2.564903	-3.806065	-3.994997
72	6	0	4.925905	-3.737117	-3.179997
73	6	0	7.910990	0.095816	-0.148997
74	6	0	7.819016	1.240818	-1.183997
75	6	0	8.261962	-1.199192	-0.905997
76	6	0	9.039997	0.406791	0.860003
77	6	0	3.330099	4.981918	-1.816997
78	6	0	3.954075	3.877904	-2.702997
79	6	0	4.416122	6.015894	-1.442997
80	6	0	-8.276024	-0.547823	-1.895997
81	6	0	-8.909995	0.768191	-2.381997
82	6	0	-9.400044	-1.449798	-1.336997
83	6	0	-7.612039	-1.241838	-3.108997
84	6	0	2.240115	5.692943	-2.639997
85	6	0	-2.531935	3.437049	-3.229997
86	6	0	-2.226902	4.931042	-3.503997
87	6	0	-1.371954	2.581023	-3.782997
88	6	0	-3.821943	3.067078	-3.985997
89	1	0	-1.699034	-0.997969	1.345003
90	1	0	-1.613995	0.752029	1.585003
91	1	0	-1.324032	-0.893978	7.237003
92	1	0	-1.380076	-2.865977	5.727003
93	1	0	-1.362069	-2.550977	3.267003
94	1	0	-1.302974	1.707022	3.807003
95	1	0	-1.293981	1.398022	6.278003
96	1	0	0.693964	-1.109023	1.765003
97	1	0	0.773000	0.514975	1.971003
98	1	0	2.380007	0.860940	0.090003
99	1	0	2.408968	-0.894061	-0.000997
100	1	0	-4.239045	-1.472913	-1.329997
101	1	0	-0.472087	-3.392997	-2.023997
102	1	0	-5.255953	2.623110	2.028003
103	1	0	-6.080939	3.267128	0.612003
104	1	0	-4.362064	-2.327910	2.191003

105	1	0	-5.361086	-3.350888	1.177003
106	1	0	-5.464989	1.018115	3.530003
107	1	0	4.775916	-3.224114	-0.733997
108	1	0	-0.441139	-5.702997	-0.150997
109	1	0	-0.407121	-4.892998	1.402003
110	1	0	-2.641896	5.166052	2.924003
111	1	0	-4.750952	2.672099	-1.668997
112	1	0	-4.184131	-5.338914	1.664003
113	1	0	0.771102	5.113975	-0.713997
114	1	0	-0.619928	3.764007	-1.348997
115	1	0	-7.270971	1.840155	-0.822997
116	1	0	3.560929	-2.663087	2.752003
117	1	0	4.928912	-3.440117	1.982003
118	1	0	4.938056	3.029883	2.633003
119	1	0	3.646035	2.091911	3.353003
120	1	0	-0.680898	5.110008	1.529003
121	1	0	-0.681932	3.591008	2.389003
122	1	0	4.666067	3.510889	0.067003
123	1	0	1.126874	-5.131032	-1.926997
124	1	0	-5.135028	-0.722893	3.704003
125	1	0	3.343959	-1.335082	4.371003
126	1	0	-6.811066	-2.421855	-0.548997
127	1	0	-2.589148	-6.111950	1.471003
128	1	0	-4.350907	4.705090	2.718003
129	1	0	0.581075	3.869980	4.106003
130	1	0	2.280064	3.397942	4.357003
131	1	0	0.965040	2.296971	4.861003
132	1	0	0.868912	-3.436027	4.105003
133	1	0	0.769879	-4.908024	3.101003
134	1	0	-3.166138	-5.645937	3.096003
135	1	0	-3.854001	0.469079	4.066003
136	1	0	2.360894	-4.231060	3.526003
137	1	0	3.336998	0.433918	4.554003
138	1	0	1.797978	-0.441047	4.328003

139	1	0	-3.452919	4.176070	4.168003
140	1	0	-4.385037	-1.120910	-3.450997
141	1	0	-3.279010	0.093066	-2.788997
142	1	0	-3.259018	-0.285935	-4.519997
143	1	0	-0.676021	-0.399992	-2.872997
144	1	0	-0.118055	-1.942005	-3.535997
145	1	0	-0.890029	-0.762987	-4.590997
146	1	0	-2.530070	-2.602951	-5.316997
147	1	0	-1.801095	-3.747967	-4.176997
148	1	0	-3.539088	-3.417928	-4.106997
149	1	0	4.391846	-6.359105	-2.475997
150	1	0	2.730845	-6.433068	-3.080997
151	1	0	4.061852	-6.117098	-4.201997
152	1	0	1.582893	-4.288043	-3.948997
153	1	0	2.419927	-2.734061	-3.827997
154	1	0	2.950900	-3.944073	-5.009997
155	1	0	5.257901	-3.911125	-4.206997
156	1	0	4.882929	-2.654116	-3.023997
157	1	0	5.689896	-4.152134	-2.514997
158	1	0	6.997012	1.067837	-1.882997
159	1	0	7.665038	2.214822	-0.711997
160	1	0	8.749017	1.302798	-1.757997
161	1	0	7.464955	-1.487174	-1.599997
162	1	0	9.176965	-1.051212	-1.487997
163	1	0	8.445943	-2.037196	-0.225997
164	1	0	9.999000	0.512770	0.342003
165	1	0	8.843018	1.338795	1.400003
166	1	0	9.136979	-0.395211	1.599003
167	1	0	4.778063	3.364886	-2.198997
168	1	0	3.206058	3.125921	-2.972997
169	1	0	4.350084	4.310896	-3.626997
170	1	0	1.786134	6.522953	-2.087997
171	1	0	2.679125	6.109933	-3.550997
172	1	0	4.831133	6.476885	-2.345997

173	1	0	4.000140	6.810903	-0.815997
174	1	0	5.243112	5.553876	-0.894997
175	1	0	-9.401983	1.306202	-1.565997
176	1	0	-8.168980	1.433175	-2.836997
177	1	0	-9.667999	0.553208	-3.141997
178	1	0	-9.883034	-0.979787	-0.474997
179	1	0	-10.163048	-1.622781	-2.102997
180	1	0	-9.022066	-2.425806	-1.018997
181	1	0	-8.351043	-1.424821	-3.896997
182	1	0	-6.818025	-0.613855	-3.527997
183	1	0	-7.170061	-2.204847	-2.834997
184	1	0	1.445100	5.002960	-2.942997
185	1	0	6.448035	2.079849	1.043003
186	1	0	6.424940	-2.174151	0.512003
187	1	0	-3.671940	3.202075	-5.060997
188	1	0	-3.022887	5.571060	-3.108997
189	1	0	-1.284895	5.242021	-3.041997
190	1	0	-2.145898	5.111041	-4.580997
191	1	0	-1.256950	2.748021	-4.859997
192	1	0	-0.415949	2.828002	-3.309997
193	1	0	-1.560978	1.518027	-3.625997
194	1	0	-4.659929	3.706097	-3.688997
195	1	0	-4.109967	2.025084	-3.818997
196	1	0	1.703970	-0.818045	-2.377997
197	1	0	1.610009	0.937957	-2.319997
198	1	0	-0.081992	0.874995	-0.292997
199	1	0	-0.078032	-0.892006	-0.490997
200	6	0	2.138990	0.068945	-1.901997
201	6	0	3.626992	0.155912	-2.253997
202	1	0	4.058012	1.050902	-1.786997
203	1	0	4.153973	-0.698100	-1.805997
204	6	0	3.876992	0.184906	-3.764997
205	1	0	3.487972	-0.739085	-4.211997
206	1	0	3.297011	1.002919	-4.213997

207	6	0	5.352996	0.347873	-4.131997
208	1	0	5.504995	0.310870	-5.214997
209	1	0	5.740017	1.308865	-3.775997
210	1	0	5.963978	-0.443140	-3.681997

2e⊂1

Center Number	Atomic Number	Atomic Type	Coordinates (Angstroms) X	Y	Z
1	8	0	-1.660430	3.050764	2.143986
2	6	0	-1.834575	4.065739	1.196986
3	6	0	-3.074597	4.222562	0.565986
4	6	0	-3.191734	5.180546	-0.448014
5	6	0	-2.125852	6.001698	-0.830014
6	6	0	-0.924830	5.853869	-0.123014
7	6	0	-0.750693	4.895894	0.878986
8	6	0	0.589328	4.746086	1.561986
9	6	0	1.555462	3.811224	0.848986
10	6	0	1.591475	3.720229	-0.549014
11	6	0	2.479596	2.871356	-1.213014
12	6	0	3.341708	2.089479	-0.430014
13	6	0	3.344699	2.146479	0.963986
14	6	0	2.445574	3.024351	1.591986
15	8	0	2.381573	3.031342	2.979986
16	6	0	3.179429	4.039456	3.611986
17	1	0	4.237444	3.932607	3.346986
18	1	0	2.843285	5.045408	3.333986
19	1	0	3.059449	3.901439	4.688986
20	6	0	4.275821	1.297612	1.823986
21	6	0	4.924983	0.161705	1.072986
22	6	0	6.019947	0.413862	0.239986
23	6	0	6.578087	-0.565059	-0.591014

24	6	0	6.014270	-1.845139	-0.532014
25	6	0	4.938314	-2.151293	0.307986
26	6	0	4.395168	-1.134370	1.103986
27	8	0	3.282207	-1.406530	1.894986
28	6	0	3.583281	-1.921486	3.195986
29	1	0	4.261403	-2.779390	3.143986
30	1	0	4.042170	-1.149421	3.827986
31	1	0	2.636327	-2.246622	3.628986
32	6	0	4.364514	-3.552375	0.334986
33	6	0	2.986532	-3.682572	-0.293014
34	6	0	2.762472	-3.258604	-1.609014
35	6	0	1.527495	-3.424780	-2.242014
36	6	0	0.500582	-4.031927	-1.504014
37	6	0	0.679643	-4.458901	-0.189014
38	6	0	1.932616	-4.269722	0.412986
39	8	0	2.121670	-4.648695	1.738986
40	6	0	2.540865	-6.007635	1.907986
41	1	0	2.654888	-6.169619	2.981986
42	1	0	3.498891	-6.191499	1.406986
43	1	0	1.796965	-6.709742	1.511986
44	6	0	-0.432253	-5.183060	0.547986
45	6	0	-1.817338	-4.593258	0.379986
46	6	0	-2.742240	-5.278390	-0.419014
47	6	0	-4.052303	-4.835578	-0.619014
48	6	0	-4.427470	-3.667631	0.050986
49	6	0	-3.538575	-2.931504	0.838986
50	6	0	-2.220510	-3.390316	0.985986
51	8	0	-1.341621	-2.608190	1.733986
52	6	0	-0.802536	-3.204113	2.937986
53	1	0	-1.396412	-4.075198	3.227986
54	1	0	-0.861643	-2.459122	3.729986
55	1	0	0.237506	-3.497965	2.775986
56	6	0	-4.016757	-1.661572	1.499986
57	6	0	-4.101930	-0.446585	0.580986

58	6	0	−4.188914	−0.556597	−0.807014
59	6	0	−4.298074	0.562387	−1.651014
60	6	0	−4.320254	1.820384	−1.050014
61	6	0	−4.240277	1.981396	0.338986
62	6	0	−4.132114	0.843411	1.141986
63	8	0	−4.001136	0.991430	2.519986
64	6	0	−5.228126	0.923254	3.256986
65	1	0	−4.966147	1.069292	4.306986
66	1	0	−5.919239	1.712156	2.939986
67	1	0	−5.716987	−0.049815	3.135986
68	6	0	−4.271476	3.371391	0.944986
69	1	0	−4.342464	3.289381	2.027986
70	1	0	−5.177549	3.883262	0.603986
71	1	0	−4.387382	2.718375	−1.654014
72	6	0	−4.380045	0.356376	−3.172014
73	6	0	−4.414236	1.690371	−3.938014
74	1	0	−5.293321	2.288245	−3.677014
75	1	0	−4.456208	1.498365	−5.014014
76	1	0	−3.518321	2.291499	−3.744014
77	6	0	−5.665932	−0.436808	−3.505014
78	1	0	−5.746909	−0.594820	−4.586014
79	1	0	−6.556009	0.105065	−3.171014
80	1	0	−5.668791	−1.418808	−3.024014
81	6	0	−3.148930	−0.446448	−3.653014
82	1	0	−3.090790	−1.429440	−3.178014
83	1	0	−3.199907	−0.605456	−4.736014
84	1	0	−2.219006	0.086684	−3.433014
85	1	0	−4.154773	−1.546592	−1.245014
86	1	0	−5.016730	−1.848715	1.910986
87	1	0	−3.381791	−1.418482	2.354986
88	1	0	−5.444523	−3.296776	−0.039014
89	6	0	−5.062199	−5.562722	−1.521014
90	6	0	−4.448023	−6.795634	−2.208014
91	1	0	−3.594062	−6.525512	−2.839014

92	1	0	-4.115916	-7.544587	-1.481014
93	1	0	-5.195955	-7.271741	-2.850014
94	6	0	-6.269133	-6.025894	-0.674014
95	1	0	-7.004060	-6.536999	-1.306014
96	1	0	-5.952034	-6.720849	0.109986
97	1	0	-6.775253	-5.183967	-0.192014
98	6	0	-5.548338	-4.588791	-2.620014
99	1	0	-6.255267	-5.089892	-3.289014
100	1	0	-4.707390	-4.227671	-3.222014
101	1	0	-6.056463	-3.717864	-2.195014
102	1	0	-2.404109	-6.194342	-0.891014
103	1	0	-0.467106	-6.216065	0.180986
104	1	0	-0.169243	-5.256023	1.601986
105	1	0	-0.474396	-4.183066	-1.956014
106	6	0	1.261434	-2.992818	-3.695014
107	6	0	2.485337	-2.312643	-4.334014
108	1	0	2.786209	-1.416600	-3.782014
109	1	0	2.246293	-2.006678	-5.357014
110	1	0	3.344433	-2.988521	-4.385014
111	6	0	0.076292	-2.000988	-3.732014
112	1	0	-0.131752	-1.692017	-4.762014
113	1	0	0.295164	-1.102956	-3.147014
114	1	0	-0.837645	-2.444118	-3.327014
115	6	0	0.904612	-4.239869	-4.536014
116	1	0	1.725715	-4.963752	-4.525014
117	1	0	0.009684	-4.741997	-4.156014
118	1	0	0.712571	-3.955897	-5.577014
119	1	0	3.589405	-2.789486	-2.130014
120	1	0	5.054609	-4.217276	-0.198014
121	1	0	4.313566	-3.915382	1.361986
122	1	0	6.411384	-2.642082	-1.149014
123	6	0	7.735034	-0.197893	-1.534014
124	6	0	7.258876	0.911039	-2.502014
125	1	0	6.398924	0.572916	-3.090014

126	1	0	8.060837	1.182153	-3.197014
127	1	0	6.964746	1.818997	-1.967014
128	6	0	8.208206	-1.398826	-2.373014
129	1	0	7.408262	-1.789940	-3.011014
130	1	0	8.577322	-2.213773	-1.743014
131	1	0	9.029162	-1.090708	-3.027014
132	6	0	8.932961	0.319278	-0.707014
133	1	0	8.670834	1.204240	-0.119014
134	1	0	9.290070	-0.449671	-0.015014
135	1	0	9.761921	0.594396	-1.368014
136	1	0	6.416803	1.424918	0.222986
137	1	0	5.057728	1.945724	2.238986
138	1	0	3.707874	0.927531	2.679986
139	1	0	4.031805	1.410578	-0.915014
140	6	0	2.567607	2.795368	-2.747014
141	6	0	3.930523	3.379563	-3.189014
142	1	0	4.024528	3.347577	-4.280014
143	1	0	4.763604	2.812682	-2.763014
144	1	0	4.030374	4.420577	-2.867014
145	6	0	2.475817	1.326355	-3.217014
146	1	0	1.512880	0.884218	-2.943014
147	1	0	2.570825	1.268369	-4.306014
148	1	0	3.264905	0.705468	-2.784014
149	6	0	1.442493	3.590208	-3.435014
150	1	0	1.523509	3.482219	-4.521014
151	1	0	1.498340	4.659216	-3.208014
152	1	0	0.451544	3.231066	-3.137014
153	1	0	0.890389	4.318129	-1.114014
154	1	0	1.051186	5.739152	1.635986
155	1	0	0.465379	4.388068	2.583986
156	1	0	-0.079922	6.494990	-0.361014
157	6	0	-2.220998	7.024684	-1.973014
158	6	0	-1.946200	8.442724	-1.423014
159	1	0	-2.683239	8.715618	-0.661014

160	1	0	-0.952211	8.520866	-0.972014
161	1	0	-2.003306	9.180715	-2.231014
162	6	0	-1.163947	6.672835	-3.047014
163	1	0	-1.328803	5.664812	-3.442014
164	1	0	-1.220048	7.379827	-3.881014
165	1	0	-0.146953	6.713981	-2.646014
166	6	0	-3.606998	7.023486	-2.644014
167	1	0	-3.847858	6.045452	-3.075014
168	1	0	-3.623102	7.755484	-3.456014
169	1	0	-4.400036	7.295373	-1.940014
170	1	0	-4.154748	5.279408	-0.935014
171	6	0	-1.967486	3.441720	3.493986
172	1	0	-1.685367	2.608761	4.135986
173	1	0	-3.035515	3.645568	3.611986
174	1	0	-1.399613	4.334802	3.775986
175	6	0	0.076929	0.540013	0.234986
176	6	0	0.756115	-0.763890	-0.157014
177	1	0	1.381090	-0.583801	-1.032014
178	1	0	0.022225	-1.530995	-0.413014
179	1	0	1.408171	-1.156797	0.622986
180	6	0	-0.934140	1.020868	-0.792014
181	1	0	-0.420161	1.170941	-1.744014
182	1	0	-1.389276	1.971803	-0.507014
183	1	0	-1.723034	0.283755	-0.945014
184	7	0	-0.621045	0.357913	1.587986
185	1	0	-1.044910	-0.586148	1.621986
186	6	0	0.328927	0.554049	2.737986
187	6	0	-0.193001	0.045974	4.059986
188	6	0	0.739057	-0.353893	5.026986
189	6	0	0.315125	-0.829953	6.266986
190	6	0	-1.048863	-0.917148	6.554986
191	6	0	-1.980921	-0.510282	5.600986
192	6	0	-1.558991	-0.023221	4.360986
193	1	0	-2.308035	0.287672	3.640986

194	1	0	-3.043912	-0.571433	5.815986
195	1	0	-1.379808	-1.298196	7.515986
196	1	0	1.049169	-1.139849	7.003986
197	1	0	1.800048	-0.290741	4.804986
198	1	0	0.525774	1.626077	2.779986
199	1	0	1.261999	0.052182	2.474986
200	1	0	-1.364147	1.067807	1.673986
201	1	0	0.816818	1.318118	0.411986

2f⊂1

Center Number	Atomic Number	Atomic Type	Coordinates (Angstroms) X	Y	Z
1	8	0	-2.693901	-2.168105	2.082057
2	6	0	-3.774893	-2.338156	1.223057
3	6	0	-3.921835	-3.556163	0.545057
4	6	0	-5.001828	-3.710214	-0.329943
5	6	0	-5.955875	-2.704259	-0.524943
6	6	0	-5.776932	-1.510250	0.184057
7	6	0	-4.683942	-1.289199	1.029057
8	6	0	-4.487006	0.054811	1.694057
9	6	0	-4.027059	1.190832	0.780057
10	6	0	-4.282060	1.205820	-0.590943
11	6	0	-3.903110	2.270838	-1.422943
12	6	0	-3.294162	3.370867	-0.819943
13	6	0	-2.996164	3.399881	0.549057
14	6	0	-3.337111	2.290865	1.333057
15	8	0	-2.902108	2.227885	2.654057
16	6	0	-3.833129	2.674842	3.645057
17	1	0	-4.112179	3.721828	3.480057
18	1	0	-4.741100	2.061799	3.651057
19	1	0	-3.324125	2.578866	4.606057

20	6	0	-2.312221	4.620913	1.141057
21	6	0	-0.937235	4.908978	0.564057
22	6	0	-0.790276	5.781985	-0.520943
23	6	0	0.457710	6.074044	-1.081943
24	6	0	1.582737	5.499097	-0.474943
25	6	0	1.487779	4.607093	0.597057
26	6	0	0.210794	4.304032	1.089057
27	8	0	0.067839	3.342026	2.086057
28	6	0	0.027816	3.842024	3.434057
29	1	0	-0.551228	4.768996	3.483057
30	1	0	1.038807	4.034072	3.808057
31	1	0	-0.449148	3.075001	4.040057
32	6	0	2.727809	3.990151	1.205057
33	6	0	3.336864	2.818180	0.442057
34	6	0	3.283869	2.718178	-0.947943
35	6	0	3.904918	1.676207	-1.652943
36	6	0	4.585963	0.713239	-0.906943
37	6	0	4.652961	0.759242	0.492057
38	6	0	4.027911	1.820213	1.156057
39	8	0	4.042909	1.868213	2.545057
40	6	0	5.157876	2.573266	3.099057
41	1	0	6.106898	2.106311	2.809057
42	1	0	5.048878	2.528261	4.185057
43	1	0	5.165826	3.622266	2.779057
44	6	0	5.374013	-0.336724	1.266057
45	6	0	4.948078	-1.721744	0.825057
46	6	0	5.652110	-2.394711	-0.179943
47	6	0	5.235169	-3.632730	-0.682943
48	6	0	4.080195	-4.192785	-0.120943
49	6	0	3.338165	-3.553820	0.876057
50	6	0	3.794106	-2.314798	1.349057
51	8	0	3.081075	-1.647832	2.342057
52	6	0	3.481092	-2.015813	3.670057
53	1	0	4.531080	-1.754764	3.850057

54	1	0	3.352143	-3.090819	3.839057
55	1	0	2.843066	-1.458843	4.357057
56	6	0	2.065194	-4.180880	1.398057
57	6	0	0.862192	-4.136937	0.458057
58	6	0	0.967181	-3.899932	-0.911943
59	6	0	-0.146819	-3.902984	-1.767943
60	6	0	-1.393806	-4.164043	-1.198943
61	6	0	-1.548795	-4.402051	0.174057
62	6	0	-0.416796	-4.376997	0.994057
63	8	0	-0.554788	-4.544004	2.367057
64	6	0	-0.440725	-5.897998	2.816057
65	1	0	-1.212694	-6.534035	2.365057
66	1	0	-0.573725	-5.879005	3.899057
67	1	0	0.543296	-6.321952	2.582057
68	6	0	-2.927782	-4.684116	0.744057
69	1	0	-2.830771	-4.909111	1.807057
70	1	0	-3.328739	-5.585135	0.267057
71	1	0	-2.289806	-4.170086	-1.808943
72	6	0	0.054170	-3.668975	-3.275943
73	6	0	0.887110	-2.389936	-3.528943
74	1	0	1.063104	-2.262927	-4.602943
75	1	0	1.865112	-2.425889	-3.038943
76	1	0	0.363068	-1.498960	-3.170943
77	6	0	-1.283837	-3.515038	-4.022943
78	1	0	-1.874876	-2.683066	-3.623943
79	1	0	-1.889794	-4.425067	-3.964943
80	1	0	-1.096846	-3.315029	-5.081943
81	6	0	0.813228	-4.882939	-3.862943
82	1	0	1.795233	-4.999893	-3.394943
83	1	0	0.250271	-5.807966	-3.701943
84	1	0	0.966222	-4.757932	-4.939943
85	1	0	1.949172	-3.701885	-1.322943
86	1	0	2.275244	-5.232870	1.632057
87	1	0	1.765173	-3.717894	2.339057

88	1	0	3.725240	-5.155802	-0.474943
89	6	0	5.964203	-4.361696	-1.822943
90	6	0	4.983212	-4.544742	-3.005943
91	1	0	4.613166	-3.576760	-3.359943
92	1	0	5.482235	-5.045719	-3.841943
93	1	0	4.118240	-5.153783	-2.724943
94	6	0	7.186166	-3.576638	-2.331943
95	1	0	7.933159	-3.430603	-1.544943
96	1	0	7.667192	-4.129615	-3.143943
97	1	0	6.902120	-2.594652	-2.725943
98	6	0	6.442268	-5.745673	-1.330943
99	1	0	6.953294	-6.281649	-2.138943
100	1	0	5.608298	-6.368713	-0.994943
101	1	0	7.142263	-5.641640	-0.495943
102	1	0	6.544088	-1.920669	-0.572943
103	1	0	6.455008	-0.231673	1.115057
104	1	0	5.179006	-0.194733	2.329057
105	1	0	5.058003	-0.123739	-1.403943
106	6	0	3.827920	1.637203	-3.188943
107	6	0	4.524860	2.896236	-3.755943
108	1	0	4.034817	3.814213	-3.415943
109	1	0	5.571858	2.938286	-3.441943
110	1	0	4.495861	2.889235	-4.850943
111	6	0	2.351920	1.626134	-3.647943
112	1	0	1.843963	0.719110	-3.307943
113	1	0	2.296919	1.650131	-4.741943
114	1	0	1.795879	2.489107	-3.268943
115	6	0	4.516979	0.390236	-3.774943
116	1	0	4.057022	-0.534786	-3.407943
117	1	0	4.425978	0.396231	-4.864943
118	1	0	5.583980	0.363286	-3.533943
119	1	0	2.736833	3.475152	-1.497943
120	1	0	3.491772	4.775187	1.290057
121	1	0	2.522825	3.653142	2.221057

122	1	0	2.574726	5.741144	-0.845943
123	6	0	0.632668	6.970052	-2.317943
124	6	0	1.507610	8.192094	-1.957943
125	1	0	1.036582	8.789071	-1.170943
126	1	0	1.646580	8.832100	-2.834943
127	1	0	2.499624	7.894141	-1.604943
128	6	0	-0.713356	7.478989	-2.864943
129	1	0	-0.541385	8.095997	-3.751943
130	1	0	-1.242385	8.096964	-2.132943
131	1	0	-1.370317	6.653958	-3.159943
132	6	0	1.325707	6.148085	-3.431943
133	1	0	0.728748	5.268057	-3.694943
134	1	0	1.450678	6.757091	-4.332943
135	1	0	2.317723	5.802132	-3.125943
136	1	0	-1.686298	6.240943	-0.922943
137	1	0	-2.949262	5.491883	0.953057
138	1	0	-2.248216	4.500916	2.220057
139	1	0	-2.993202	4.223881	-1.415943
140	6	0	-4.143106	2.170827	-2.937943
141	6	0	-3.578163	3.385854	-3.695943
142	1	0	-4.063207	4.318831	-3.389943
143	1	0	-3.750157	3.263845	-4.768943
144	1	0	-2.498168	3.492905	-3.541943
145	6	0	-3.447045	0.897860	-3.476943
146	1	0	-3.838002	-0.012159	-3.012943
147	1	0	-3.601041	0.811852	-4.557943
148	1	0	-2.369047	0.933911	-3.288943
149	6	0	-5.661101	2.080755	-3.212943
150	1	0	-6.106060	1.205734	-2.731943
151	1	0	-5.850097	2.000746	-4.288943
152	1	0	-6.179143	2.970731	-2.840943
153	1	0	-4.766019	0.343797	-1.030943
154	1	0	-5.438020	0.359766	2.151057
155	1	0	-3.769001	-0.035155	2.510057

156	1	0	-6.502970	-0.709285	0.075057
157	6	0	-7.159868	-2.862316	-1.467943
158	6	0	-7.143804	-4.208315	-2.214943
159	1	0	-7.200764	-5.056318	-1.524943
160	1	0	-8.008801	-4.269356	-2.881943
161	1	0	-6.244799	-4.321272	-2.827943
162	6	0	-8.465872	-2.774377	-0.644943
163	1	0	-8.509834	-3.572379	0.103057
164	1	0	-8.550917	-1.818381	-0.120943
165	1	0	-9.336867	-2.874418	-1.301943
166	6	0	-7.138921	-1.727315	-2.518943
167	1	0	-7.974916	-1.838354	-3.216943
168	1	0	-6.207921	-1.744271	-3.095943
169	1	0	-7.230968	-0.742319	-2.052943
170	1	0	-5.093783	-4.655218	-0.851943
171	6	0	-2.933880	-2.615116	3.422057
172	1	0	-3.326831	-3.636135	3.440057
173	1	0	-1.971880	-2.599071	3.935057
174	1	0	-3.646911	-1.954150	3.934057
175	6	0	-0.080013	0.207019	-0.133943
176	6	0	-1.158964	-0.820032	-0.463943
177	1	0	-2.158984	-0.406079	-0.330943
178	1	0	-1.056950	-1.117027	-1.507943
179	1	0	-1.064922	-1.722028	0.140057
180	6	0	-0.243073	1.480011	-0.966943
181	1	0	0.550893	2.201049	-0.757943
182	1	0	-1.209095	1.953965	-0.794943
183	1	0	-0.182060	1.217014	-2.024943
184	6	0	1.326014	-0.366915	-0.289943
185	1	0	1.422061	-1.357910	0.152057
186	1	0	1.548019	-0.467904	-1.351943
187	1	0	2.077983	0.292121	0.144057
188	7	0	-0.268033	0.635010	1.336057
189	1	0	-1.237048	0.948964	1.466057

190	1	0	0.275927	1.492036	1.530057
191	6	0	0.044016	-0.397975	2.399057
192	6	0	0.115985	0.255028	3.755057
193	6	0	1.334960	0.790086	4.196057
194	6	0	1.420931	1.393090	5.451057
195	6	0	0.292927	1.474036	6.271057
196	6	0	-0.928048	0.957979	5.827057
197	6	0	-1.016020	0.351975	4.572057
198	1	0	-1.963001	-0.044070	4.224057
199	1	0	-1.807051	1.017937	6.462057
200	1	0	0.361905	1.938040	7.250057
201	1	0	2.369912	1.800134	5.788057
202	1	0	2.211962	0.743127	3.557057
203	1	0	1.005037	-0.841930	2.148057
204	1	0	-0.734948	-1.156012	2.335057

2g⊂1

Center Number	Atomic Number	Atomic Type	Coordinates (Angstroms) X	Y	Z
1	8	0	-4.149930	1.453220	2.458996
2	6	0	-4.166915	1.708221	1.090996
3	6	0	-4.811969	0.810260	0.225996
4	6	0	-4.700957	1.010253	-1.151004
5	6	0	-3.992892	2.086210	-1.699004
6	6	0	-3.403837	2.985174	-0.804004
7	6	0	-3.479847	2.822179	0.583996
8	6	0	-2.871786	3.842142	1.540996
9	6	0	-1.558749	4.440063	1.090996
10	6	0	-1.538687	5.464062	0.131996
11	6	0	-0.349654	6.022990	-0.339004
12	6	0	0.841319	5.574918	0.250996

13	6	0	0.876258	4.570916	1.218996
14	6	0	-0.341777	3.986989	1.606996
15	8	0	-0.331841	2.929989	2.525996
16	6	0	-0.298816	3.344987	3.904996
17	1	0	0.176136	2.542958	4.471996
18	1	0	0.281240	4.263952	4.019996
19	1	0	-1.313805	3.522048	4.281996
20	6	0	2.192234	4.158836	1.843996
21	6	0	3.009172	3.132787	1.073996
22	6	0	3.325183	3.323767	-0.277004
23	6	0	4.162130	2.448717	-0.973004
24	6	0	4.695065	1.364685	-0.261004
25	6	0	4.391050	1.120703	1.077996
26	6	0	3.527104	2.012755	1.735996
27	8	0	3.149089	1.763778	3.050996
28	6	0	4.058119	2.272723	4.031996
29	1	0	3.651103	1.999748	5.007996
30	1	0	4.145186	3.364718	3.965996
31	1	0	5.057093	1.833663	3.921996
32	6	0	4.980978	-0.065333	1.828996
33	6	0	5.004900	-1.350334	1.031996
34	6	0	6.039885	-1.607397	0.129996
35	6	0	6.060816	-2.737398	-0.697004
36	6	0	5.019761	-3.658335	-0.540004
37	6	0	3.984773	-3.463273	0.381996
38	6	0	3.970843	-2.290272	1.145996
39	8	0	2.899859	-2.039207	1.996996
40	6	0	3.084831	-2.504218	3.338996
41	1	0	4.016854	-2.120274	3.769996
42	1	0	3.099764	-3.599219	3.384996
43	1	0	2.237853	-2.135167	3.917996
44	6	0	2.889710	-4.493206	0.542996
45	6	0	1.554735	-4.077126	-0.045004
46	6	0	1.474763	-3.617121	-1.366004

47	6	0	0.258785	-3.264047	-1.953004
48	6	0	-0.897222	-3.366977	-1.163004
49	6	0	-0.865249	-3.824979	0.153996
50	6	0	0.379729	-4.179054	0.701996
51	8	0	0.428704	-4.603057	2.026996
52	6	0	0.355618	-6.022053	2.202996
53	1	0	0.368607	-6.204054	3.279996
54	1	0	-0.566407	-6.433997	1.775996
55	1	0	1.213587	-6.524105	1.738996
56	6	0	-2.116260	-3.993903	1.010996
57	6	0	-3.375225	-3.429827	0.401996
58	6	0	-3.971264	-4.060791	-0.701004
59	6	0	-5.080231	-3.528724	-1.360004
60	6	0	-5.624160	-2.339691	-0.844004
61	6	0	-5.077120	-1.688724	0.259996
62	6	0	-3.941154	-2.243793	0.865996
63	8	0	-3.365113	-1.566828	1.942996
64	6	0	-3.914135	-1.936795	3.221996
65	1	0	-3.561091	-1.200816	3.940996
66	1	0	-5.007135	-1.930729	3.185996
67	1	0	-3.567195	-2.932816	3.511996
68	6	0	-5.624040	-0.364691	0.745996
69	1	0	-5.645038	-0.334690	1.834996
70	1	0	-6.658034	-0.256629	0.398996
71	1	0	-6.495133	-1.897639	-1.318004
72	6	0	-5.678269	-4.154688	-2.629004
73	6	0	-4.964348	-5.457731	-3.031004
74	1	0	-5.425373	-5.866703	-3.935004
75	1	0	-3.904338	-5.290795	-3.250004
76	1	0	-5.039394	-6.219727	-2.248004
77	6	0	-5.528208	-3.143697	-3.792004
78	1	0	-6.057152	-2.207665	-3.589004
79	1	0	-5.937234	-3.563672	-4.717004
80	1	0	-4.473194	-2.903761	-3.965004

81	6	0	-7.175288	-4.467597	-2.406004
82	1	0	-7.609315	-4.905571	-3.311004
83	1	0	-7.305331	-5.180590	-1.586004
84	1	0	-7.751234	-3.569563	-2.165004
85	1	0	-3.513319	-4.977819	-1.056004
86	1	0	-2.267324	-5.064894	1.197996
87	1	0	-1.928232	-3.545915	1.988996
88	1	0	-1.855206	-3.103919	-1.591004
89	6	0	0.133810	-2.849040	-3.430004
90	6	0	-0.757115	-1.595986	-3.577004
91	1	0	-0.327063	-0.743012	-3.046004
92	1	0	-0.849098	-1.319980	-4.633004
93	1	0	-1.769124	-1.753924	-3.195004
94	6	0	-0.522261	-4.019000	-4.202004
95	1	0	-0.627246	-3.768994	-5.263004
96	1	0	0.081684	-4.927036	-4.122004
97	1	0	-1.519275	-4.241940	-3.806004
98	6	0	1.502828	-2.538122	-4.064004
99	1	0	1.364848	-2.209114	-5.099004
100	1	0	2.026877	-1.742154	-3.525004
101	1	0	2.150775	-3.419162	-4.088004
102	1	0	2.400768	-3.533177	-1.922004
103	1	0	3.212654	-5.424226	0.060996
104	1	0	2.749696	-4.721198	1.599996
105	1	0	4.998706	-4.565334	-1.134004
106	6	0	7.186806	-2.905466	-1.730004
107	6	0	8.552804	-2.950549	-1.010004
108	1	0	8.599752	-3.796552	-0.317004
109	1	0	8.743859	-2.037560	-0.438004
110	1	0	9.363797	-3.061598	-1.738004
111	6	0	7.154879	-1.701464	-2.703004
112	1	0	7.937873	-1.802512	-3.462004
113	1	0	7.319936	-0.753474	-2.181004
114	1	0	6.188883	-1.639406	-3.215004

115	6	0	7.031728	-4.195457	-2.557004
116	1	0	7.071674	-5.088459	-1.926004
117	1	0	6.089727	-4.208400	-3.116004
118	1	0	7.846724	-4.268506	-3.283004
119	1	0	6.848929	-0.884446	0.067996
120	1	0	6.007993	0.183605	2.125996
121	1	0	4.406970	-0.200298	2.743996
122	1	0	5.336022	0.659646	-0.776004
123	6	0	4.492140	2.609697	-2.466004
124	6	0	3.834216	3.861737	-3.077004
125	1	0	2.741213	3.823803	-3.011004
126	1	0	4.096220	3.937721	-4.136004
127	1	0	4.175271	4.778716	-2.586004
128	6	0	3.982065	1.367728	-3.238004
129	1	0	4.228070	1.455713	-4.301004
130	1	0	2.896059	1.267793	-3.147004
131	1	0	4.435009	0.444700	-2.865004
132	6	0	6.022147	2.727604	-2.647004
133	1	0	6.273154	2.847589	-3.706004
134	1	0	6.415199	3.592580	-2.102004
135	1	0	6.544093	1.836573	-2.283004
136	1	0	2.895235	4.177793	-0.783004
137	1	0	2.804288	5.061799	1.957996
138	1	0	2.024210	3.764846	2.842996
139	1	0	1.783347	6.028861	-0.041004
140	6	0	-0.301591	7.066987	-1.464004
141	6	0	0.384487	8.356945	-0.959004
142	1	0	0.428532	9.100943	-1.760004
143	1	0	-0.169486	8.790979	-0.121004
144	1	0	1.409476	8.170883	-0.624004
145	6	0	0.508374	6.476938	-2.644004
146	1	0	0.041318	5.556966	-3.014004
147	1	0	0.553417	7.192935	-3.471004
148	1	0	1.535359	6.236876	-2.355004

149	6	0	-1.704568	7.433072	-1.981004
150	1	0	-1.620524	8.170067	-2.785004
151	1	0	-2.325542	7.875109	-1.195004
152	1	0	-2.227621	6.561103	-2.388004
153	1	0	-2.490666	5.821119	-0.241004
154	1	0	-3.594736	4.657186	1.675996
155	1	0	-2.753814	3.368135	2.514996
156	1	0	-2.849786	3.828141	-1.187004
157	6	0	-3.891882	2.243204	-3.224004
158	6	0	-5.309871	2.434290	-3.810004
159	1	0	-5.259864	2.545287	-4.899004
160	1	0	-5.955923	1.578329	-3.592004
161	1	0	-5.786817	3.328319	-3.397004
162	6	0	-3.252959	0.971165	-3.826004
163	1	0	-3.179954	1.061161	-4.915004
164	1	0	-3.839014	0.073201	-3.606004
165	1	0	-2.245969	0.818104	-3.430004
166	6	0	-3.028809	3.453152	-3.630004
167	1	0	-3.452752	4.396177	-3.267004
168	1	0	-2.004814	3.368090	-3.251004
169	1	0	-2.974805	3.518148	-4.721004
170	1	0	-5.180000	0.290282	-1.806004
171	6	0	-5.199892	2.083283	3.202996
172	1	0	-5.071911	1.775275	4.241996
173	1	0	-6.185911	1.761343	2.844996
174	1	0	-5.137826	3.175279	3.135996
175	6	0	-0.985000	0.300028	0.068996
176	7	0	-1.211004	0.226042	1.559996
177	1	0	-2.139031	-0.206902	1.733996
178	1	0	-1.216946	1.188042	1.929996
179	6	0	-0.185053	-0.575020	2.322996
180	6	0	-0.681074	-0.922990	3.705996
181	6	0	-0.508153	-2.226001	4.188996
182	6	0	-0.937173	-2.562975	5.474996

183	6	0	-1.549115	-1.607938	6.287996
184	6	0	-1.740037	-0.311926	5.804996
185	6	0	-1.309016	0.028048	4.521996
186	1	0	-1.503956	1.027059	4.148996
187	1	0	-2.227992	0.435103	6.423996
188	1	0	-1.883131	-1.872918	7.285996
189	1	0	-0.798234	-3.576983	5.836996
190	1	0	-0.053198	-2.982028	3.553996
191	1	0	0.731983	0.017924	2.356996
192	1	0	0.017893	-1.477033	1.744996
193	6	0	0.357041	0.970947	-0.294004
194	6	0	0.169099	1.933958	-1.475004
195	1	0	-0.549853	2.722002	-1.232004
196	1	0	1.121128	2.409901	-1.720004
197	1	0	-0.186933	1.408980	-2.368004
198	6	0	1.449978	-0.066119	-0.584004
199	1	0	1.655939	-0.716132	0.268996
200	1	0	2.382008	0.430824	-0.844004
201	1	0	1.159939	-0.709102	-1.418004
202	1	0	0.678078	1.579928	0.559996
203	1	0	-1.837966	0.856080	-0.321004
204	1	0	-1.045062	-0.726968	-0.303004

2h⊂1

Center Number	Atomic Number	Atomic Type	Coordinates (Angstroms) X	Y	Z
1	8	0	-2.577761	2.571261	3.073988
2	6	0	-2.855799	2.697178	1.714988
3	6	0	-3.726526	1.778920	1.109988
4	6	0	-3.943557	1.883855	-0.270012
5	6	0	-3.342853	2.880034	-1.047012

6	6	0	-2.480118	3.771290	-0.400012
7	6	0	-2.212094	3.693370	0.967988
8	6	0	-1.266386	4.674651	1.645988
9	6	0	-0.007473	4.966025	0.858988
10	6	0	-0.013765	5.951023	-0.131012
11	6	0	1.109149	6.241356	-0.917012
12	6	0	2.290351	5.559707	-0.608012
13	6	0	2.355639	4.589727	0.400988
14	6	0	1.182737	4.259378	1.089988
15	8	0	1.183046	3.221378	2.025988
16	6	0	1.483923	3.634468	3.372988
17	1	0	1.167616	4.668374	3.539988
18	1	0	0.938119	2.975306	4.050988
19	1	0	2.554951	3.539786	3.571988
20	6	0	3.685829	3.950122	0.741988
21	6	0	3.913235	2.586189	0.118988
22	6	0	3.902281	2.430186	-1.269012
23	6	0	4.148648	1.195259	-1.881012
24	6	0	4.385973	0.100330	-1.045012
25	6	0	4.393943	0.203332	0.349988
26	6	0	4.167568	1.464265	0.918988
27	8	0	4.165526	1.606264	2.303988
28	6	0	5.459460	1.828649	2.874988
29	1	0	6.125714	0.974847	2.701988
30	1	0	5.309421	1.958604	3.948988
31	1	0	5.922192	2.731786	2.459988
32	6	0	4.655300	-0.998590	1.253988
33	6	0	4.175693	-2.322733	0.701988
34	6	0	4.931890	-2.984508	-0.277012
35	6	0	4.511244	-4.175633	-0.872012
36	6	0	3.316410	-4.735988	-0.398012
37	6	0	2.536229	-4.126220	0.585988
38	6	0	2.971865	-2.901090	1.112988
39	8	0	2.185676	-2.265324	2.081988

40	6	0	2.490809	-2.712233	3.419988
41	1	0	1.688706	-2.366472	4.068988
42	1	0	3.442684	-2.291951	3.759988
43	1	0	2.550133	-3.804216	3.451988
44	6	0	1.253423	-4.778601	1.050988
45	6	0	0.025336	-4.487966	0.198988
46	6	0	0.097300	-4.364945	-1.189012
47	6	0	-1.032771	-4.126280	-1.985012
48	6	0	-2.264802	-4.022646	-1.333012
49	6	0	-2.386759	-4.168683	0.054988
50	6	0	-1.233693	-4.389340	0.814988
51	8	0	-1.331660	-4.500369	2.196988
52	6	0	-1.445260	-5.846403	2.670988
53	1	0	-1.540277	-5.788431	3.756988
54	1	0	-0.559084	-6.440140	2.416988
55	1	0	-2.331113	-6.340666	2.253988
56	6	0	-3.759764	-4.151091	0.702988
57	6	0	-4.510153	-2.842313	0.575988
58	6	0	-5.611184	-2.738641	-0.272012
59	6	0	-6.366534	-1.559865	-0.389012
60	6	0	-5.975855	-0.477749	0.398988
61	6	0	-4.844843	-0.519413	1.229988
62	6	0	-4.118489	-1.709197	1.311988
63	8	0	-2.974467	-1.784857	2.101988
64	6	0	-3.197330	-2.245923	3.438988
65	1	0	-3.853070	-3.122118	3.458988
66	1	0	-2.221244	-2.533633	3.834988
67	1	0	-3.647564	-1.459057	4.057988
68	6	0	-4.414212	0.723715	1.971988
69	1	0	-3.743137	0.468915	2.792988
70	1	0	-5.300348	1.181452	2.432988
71	1	0	-6.538130	0.448084	0.369988
72	6	0	-7.542553	-1.495214	-1.376012
73	6	0	-8.263957	-0.136429	-1.337012

74	1	0	-7.595202	0.687770	-1.611012
75	1	0	-9.091957	-0.137675	-2.053012
76	1	0	-8.683020	0.074447	-0.348012
77	6	0	-8.570225	-2.599520	-1.041012
78	1	0	-8.130928	-3.599389	-1.102012
79	1	0	-9.409236	-2.563769	-1.745012
80	1	0	-8.968264	-2.468638	-0.030012
81	6	0	-6.996489	-1.712052	-2.808012
82	1	0	-6.505200	-2.683906	-2.912012
83	1	0	-7.812501	-1.670295	-3.537012
84	1	0	-6.266719	-0.936835	-3.067012
85	1	0	-5.891924	-3.613724	-0.850012
86	1	0	-4.367530	-4.939271	0.242988
87	1	0	-3.653686	-4.413059	1.755988
88	1	0	-3.172858	-3.834916	-1.896012
89	6	0	-0.887789	-4.066237	-3.516012
90	6	0	-0.574365	-5.492144	-4.026012
91	1	0	-0.470364	-5.496113	-5.117012
92	1	0	0.358747	-5.870867	-3.596012
93	1	0	-1.375158	-6.189382	-3.757012
94	6	0	0.266931	-3.122894	-3.926012
95	1	0	0.367928	-3.111864	-5.016012
96	1	0	1.229025	-3.438608	-3.513012
97	1	0	0.076627	-2.100951	-3.591012
98	6	0	-2.174937	-3.566620	-4.200012
99	1	0	-3.015735	-4.247870	-4.037012
100	1	0	-2.462232	-2.573705	-3.838012
101	1	0	-2.016958	-3.497573	-5.280012
102	1	0	1.069324	-4.447656	-1.659012
103	1	0	1.412745	-5.864554	1.071988
104	1	0	1.019334	-4.480671	2.071988
105	1	0	2.970691	-5.682091	-0.803012
106	6	0	5.272446	-4.854407	-2.021012
107	6	0	4.351455	-4.886681	-3.265012

108	1	0	4.064153	-3.871766	-3.562012
109	1	0	3.435624	-5.454953	-3.079012
110	1	0	4.867594	-5.354527	-4.110012
111	6	0	6.560221	-4.099024	-2.396012
112	1	0	7.064374	-4.614875	-3.218012
113	1	0	6.348917	-3.077087	-2.730012
114	1	0	7.262207	-4.049816	-1.557012
115	6	0	5.653874	-6.297294	-1.621012
116	1	0	6.304875	-6.299100	-0.741012
117	1	0	6.187021	-6.790135	-2.440012
118	1	0	4.773054	-6.903556	-1.388012
119	1	0	5.869755	-2.531229	-0.576012
120	1	0	5.736322	-1.072269	1.435988
121	1	0	4.193240	-0.798728	2.221988
122	1	0	4.541263	-0.875624	-1.481012
123	6	0	4.171682	1.079266	-3.415012
124	6	0	4.431111	-0.363657	-3.889012
125	1	0	5.402222	-0.736368	-3.548012
126	1	0	4.431122	-0.398657	-4.983012
127	1	0	3.656316	-1.053887	-3.536012
128	6	0	5.301414	1.982602	-3.964012
129	1	0	5.351436	1.910617	-5.056012
130	1	0	6.272503	1.682890	-3.558012
131	1	0	5.139102	3.033554	-3.706012
132	6	0	2.819545	1.540864	-4.006012
133	1	0	2.560243	2.556787	-3.696012
134	1	0	2.860550	1.525877	-5.100012
135	1	0	2.009743	0.875624	-3.692012
136	1	0	3.692021	3.306124	-1.876012
137	1	0	4.481630	4.623358	0.404988
138	1	0	3.785855	3.865152	1.823988
139	1	0	3.205283	5.789979	-1.142012
140	6	0	1.002843	7.269325	-2.054012
141	6	0	0.593435	8.644203	-1.478012

142	1	0	-0.370553	8.601917	-0.964012
143	1	0	1.340327	9.005425	-0.764012
144	1	0	0.507215	9.382178	-2.283012
145	6	0	2.329794	7.436719	-2.815012
146	1	0	2.661073	6.494818	-3.265012
147	1	0	2.202578	8.161681	-3.625012
148	1	0	3.127683	7.808956	-2.165012
149	6	0	-0.073014	6.789005	-3.058012
150	1	0	0.190277	5.809083	-3.472012
151	1	0	-0.163224	7.496979	-3.889012
152	1	0	-1.057989	6.702713	-2.589012
153	1	0	-0.934929	6.503749	-0.291012
154	1	0	-1.799667	5.620492	1.806988
155	1	0	-1.019270	4.282724	2.631988
156	1	0	-1.980346	4.538438	-0.978012
157	6	0	-3.615900	3.038952	-2.553012
158	6	0	-4.477559	1.890696	-3.114012
159	1	0	-4.633603	2.037650	-4.187012
160	1	0	-3.997269	0.915839	-2.978012
161	1	0	-5.465548	1.853403	-2.642012
162	6	0	-4.370296	4.370728	-2.775012
163	1	0	-3.779550	5.225904	-2.431012
164	1	0	-4.583339	4.516665	-3.839012
165	1	0	-5.320298	4.378446	-2.231012
166	6	0	-2.284911	3.075348	-3.339012
167	1	0	-1.643157	3.902539	-3.024012
168	1	0	-2.484949	3.203288	-4.408012
169	1	0	-1.722635	2.146515	-3.207012
170	1	0	-4.590341	1.157663	-0.739012
171	6	0	-3.426992	3.347009	3.926988
172	1	0	-3.323310	4.419039	3.719988
173	1	0	-3.109931	3.143103	4.951988
174	1	0	-4.477907	3.060696	3.806988
175	6	0	-0.594958	-0.132150	0.621988

176	7	0	0.410963	0.134149	1.723988
177	1	0	0.939708	0.993306	1.521988
178	1	0	1.084197	-0.653651	1.786988
179	6	0	-0.295092	0.318939	3.046988
180	6	0	0.590987	0.053202	4.235988
181	6	0	0.048180	-0.596959	5.351988
182	6	0	0.836256	-0.851725	6.474988
183	6	0	2.178141	-0.465326	6.490988
184	6	0	2.721949	0.182835	5.380988
185	6	0	1.934870	0.446602	4.259988
186	1	0	2.392724	0.940738	3.410988
187	1	0	3.765860	0.481146	5.379988
188	1	0	2.794202	-0.670143	7.360988
189	1	0	0.404406	-1.357853	7.332988
190	1	0	-0.991727	-0.910268	5.337988
191	1	0	-1.147890	-0.360314	3.033988
192	1	0	-0.689395	1.337822	3.038988
193	1	0	-1.421163	0.559604	0.796988
194	1	0	-0.959656	-1.147259	0.792988
195	6	0	-0.041012	0.052014	-0.795012
196	6	0	1.129273	-0.908638	-1.038012
197	1	0	1.496245	-0.814529	-2.063012
198	1	0	0.831581	-1.946726	-0.880012
199	1	0	1.976207	-0.687386	-0.387012
200	6	0	0.397554	1.511145	-1.008012
201	1	0	1.284477	1.771408	-0.421012
202	1	0	0.668505	1.676225	-2.053012
203	1	0	-0.401654	2.211907	-0.755012
204	6	0	-1.210911	-0.290333	-1.739012
205	1	0	-2.071103	0.357411	-1.549012
206	1	0	-0.904953	-0.149242	-2.780012
207	1	0	-1.528602	-1.329428	-1.616012

2i⊂1

Center Number	Atomic Number	Atomic Type	Coordinates (Angstroms) X	Y	Z
1	8	0	-0.993629	3.171966	2.112087
2	6	0	-1.042794	4.143258	1.111010
3	6	0	-2.266369	4.495194	0.528391
4	6	0	-2.257111	5.370850	-0.564773
5	6	0	-1.079367	5.931804	-1.065990
6	6	0	0.107762	5.632859	-0.383697
7	6	0	0.155750	4.742861	0.692276
8	6	0	1.467315	4.458910	1.395344
9	6	0	2.349717	3.412566	0.728522
10	6	0	2.526313	3.378351	-0.661603
11	6	0	3.334556	2.424992	-1.284113
12	6	0	3.969863	1.480210	-0.465982
13	6	0	3.841075	1.481965	0.922152
14	6	0	3.024672	2.464723	1.508365
15	8	0	2.827122	2.438163	2.885061
16	6	0	3.729134	3.256695	3.637220
17	1	0	3.629967	4.312710	3.359604
18	1	0	3.457938	3.131793	4.687602
19	1	0	4.769395	2.944528	3.488560
20	6	0	4.576182	0.480508	1.810513
21	6	0	4.971129	-0.786325	1.090310
22	6	0	6.112372	-0.805837	0.283064
23	6	0	6.442499	-1.896568	-0.531073
24	6	0	5.592070	-3.007115	-0.481158
25	6	0	4.462732	-3.048375	0.344138
26	6	0	4.159026	-1.926809	1.125598
27	8	0	3.005954	-1.924296	1.905684
28	6	0	3.187474	-2.411854	3.239177
29	1	0	3.456845	-3.472873	3.246344

30	1	0	2.231135	-2.287999	3.752039
31	1	0	3.960521	-1.841160	3.768539
32	6	0	3.586681	-4.280142	0.386080
33	6	0	2.206955	-4.114792	-0.227559
34	6	0	2.051081	-3.616659	-1.527299
35	6	0	0.799213	-3.536063	-2.148080
36	6	0	-0.313969	-3.971351	-1.413691
37	6	0	-0.202811	-4.465606	-0.114505
38	6	0	1.069053	-4.523410	0.475231
39	8	0	1.196869	-4.975507	1.785619
40	6	0	1.375558	-6.391682	1.905964
41	1	0	1.463687	-6.606578	2.972855
42	1	0	2.286206	-6.721032	1.391133
43	1	0	0.519874	-6.940124	1.494063
44	6	0	-1.411901	-5.029232	0.607745
45	6	0	-2.694874	-4.237143	0.475491
46	6	0	-3.752945	-4.790640	-0.257688
47	6	0	-4.997076	-4.166819	-0.387368
48	6	0	-5.163243	-2.954360	0.289313
49	6	0	-4.131644	-2.345174	1.008468
50	6	0	-2.883165	-2.980484	1.074918
51	8	0	-1.853455	-2.316925	1.743063
52	6	0	-1.353495	-2.945341	2.945509
53	1	0	-0.413665	-3.465430	2.744221
54	1	0	-1.190982	-2.158523	3.683728
55	1	0	-2.090262	-3.649838	3.342077
56	6	0	-4.379653	-1.016702	1.678417
57	6	0	-4.276361	0.192840	0.756899
58	6	0	-4.498444	0.098205	-0.615729
59	6	0	-4.464948	1.213023	-1.467809
60	6	0	-4.195733	2.453792	-0.889170
61	6	0	-3.946383	2.595401	0.483457
62	6	0	-3.988879	1.459333	1.295951
63	8	0	-3.674985	1.570106	2.646836

64	6	0	-4.800538	1.758687	3.512051
65	1	0	-5.484343	0.903097	3.476203
66	1	0	-5.353878	2.667249	3.246268
67	1	0	-4.399221	1.859594	4.522676
68	6	0	-3.586210	3.955815	1.049502
69	1	0	-3.560266	3.900855	2.136800
70	1	0	-4.373843	4.669171	0.784129
71	1	0	-4.140509	3.344713	-1.503276
72	6	0	-4.727792	1.019912	-2.970429
73	6	0	-6.175643	0.511156	-3.164463
74	1	0	-6.898910	1.228729	-2.764345
75	1	0	-6.389882	0.367217	-4.228828
76	1	0	-6.335848	-0.446252	-2.659786
77	6	0	-3.742807	-0.026821	-3.543913
78	1	0	-3.929906	-0.174558	-4.612649
79	1	0	-3.847778	-0.999502	-3.054424
80	1	0	-2.704935	0.297342	-3.424494
81	6	0	-4.560063	2.328755	-3.763538
82	1	0	-5.272532	3.094102	-3.439447
83	1	0	-3.548786	2.738226	-3.665183
84	1	0	-4.739574	2.142877	-4.826551
85	1	0	-4.697738	-0.879796	-1.035026
86	1	0	-3.696953	-0.885315	2.519076
87	1	0	-5.390078	-1.033477	2.107584
88	1	0	-6.123021	-2.446728	0.257220
89	6	0	-6.149070	-4.744658	-1.223943
90	6	0	-7.370184	-5.006233	-0.312839
91	1	0	-7.123597	-5.729022	0.471282
92	1	0	-7.720398	-4.090441	0.172427
93	1	0	-8.202022	-5.409939	-0.899591
94	6	0	-6.533593	-3.721311	-2.319291
95	1	0	-6.877755	-2.775950	-1.888848
96	1	0	-5.678962	-3.504460	-2.969094
97	1	0	-7.343630	-4.115012	-2.942147

98	6	0	-5.764636	-6.066399	-1.912883
99	1	0	-5.511794	-6.844929	-1.185908
100	1	0	-4.914964	-5.938363	-2.591663
101	1	0	-6.608280	-6.432186	-2.505565
102	1	0	-3.578032	-5.750133	-0.730534
103	1	0	-1.162663	-5.171324	1.659619
104	1	0	-1.601782	-6.035174	0.214164
105	1	0	-1.303882	-3.938785	-1.858435
106	6	0	0.607980	-3.042859	-3.593094
107	6	0	1.911944	-2.488801	-4.194531
108	1	0	2.310741	-1.661674	-3.599983
109	1	0	1.725099	-2.113616	-5.205398
110	1	0	2.684809	-3.260178	-4.269020
111	6	0	-0.460373	-1.925322	-3.642015
112	1	0	-1.428730	-2.261748	-3.261562
113	1	0	-0.611121	-1.591150	-4.673981
114	1	0	-0.154074	-1.060021	-3.048686
115	6	0	0.132698	-4.230863	-4.462346
116	1	0	-0.007004	-3.913372	-5.501515
117	1	0	0.868042	-5.041493	-4.448498
118	1	0	-0.818622	-4.634973	-4.103316
119	1	0	2.945156	-3.291923	-2.047408
120	1	0	3.467262	-4.609200	1.419138
121	1	0	4.105305	-5.090353	-0.139953
122	1	0	5.802187	-3.880915	-1.087198
123	6	0	7.677024	-1.823666	-1.444109
124	6	0	7.518319	-0.627096	-2.412334
125	1	0	6.614230	-0.734310	-3.021464
126	1	0	8.378134	-0.565530	-3.087705
127	1	0	7.452187	0.323916	-1.875435
128	6	0	7.859141	-3.100640	-2.284263
129	1	0	8.003605	-3.984045	-1.654081
130	1	0	8.744620	-3.001078	-2.919039
131	1	0	7.001303	-3.280843	-2.940577

132	6	0	8.944130	-1.622762	-0.581669
133	1	0	9.081498	-2.460343	0.109466
134	1	0	9.831928	-1.557580	-1.219594
135	1	0	8.891970	-0.704289	0.010332
136	1	0	6.738132	0.082137	0.271899
137	1	0	3.946396	0.258925	2.673393
138	1	0	5.479088	0.964078	2.205639
139	1	0	4.589232	0.718507	-0.921234
140	6	0	3.581831	2.396004	-2.801913
141	6	0	3.239677	0.998982	-3.367522
142	1	0	2.182912	0.762250	-3.213739
143	1	0	3.436809	0.965415	-4.444049
144	1	0	3.832976	0.208679	-2.898585
145	6	0	5.077591	2.692685	-3.064039
146	1	0	5.723443	1.945095	-2.594722
147	1	0	5.355235	3.674891	-2.668610
148	1	0	5.284864	2.686226	-4.139550
149	6	0	2.738286	3.442266	-3.553138
150	1	0	2.982909	4.462013	-3.238855
151	1	0	2.937212	3.375537	-4.626907
152	1	0	1.665703	3.285698	-3.403419
153	1	0	2.000536	4.110038	-1.259057
154	1	0	1.278817	4.136785	2.419044
155	1	0	2.029138	5.399788	1.457841
156	1	0	1.034438	6.107035	-0.692619
157	6	0	-1.043096	6.838736	-2.305864
158	6	0	-2.437652	7.031072	-2.928980
159	1	0	-3.129680	7.519890	-2.235625
160	1	0	-2.360132	7.665594	-3.816707
161	1	0	-2.877079	6.077830	-3.242055
162	6	0	-0.483505	8.227507	-1.922455
163	1	0	-1.116194	8.708655	-1.169967
164	1	0	0.530275	8.161728	-1.516753
165	1	0	-0.446247	8.877502	-2.802894

166	6	0	-0.125888	6.186254	-3.367959
167	1	0	0.899921	6.071382	-3.005841
168	1	0	-0.091603	6.802537	-4.272458
169	1	0	-0.495558	5.193221	-3.644979
170	1	0	-3.210726	5.621357	-1.014739
171	6	0	-1.165574	3.633796	3.468597
172	1	0	-2.190558	3.444458	3.796456
173	1	0	-0.939624	4.701487	3.535669
174	1	0	-0.476325	3.068835	4.097866
175	6	0	0.082447	0.630508	0.114396
176	6	0	0.845001	-0.605782	-0.334144
177	1	0	0.191894	-1.481958	-0.362307
178	1	0	1.230466	-0.437198	-1.340203
179	1	0	1.698722	-0.828242	0.303606
180	6	0	-1.052624	1.041240	-0.824950
181	6	0	-0.567643	1.835327	-2.038692
182	1	0	-0.063035	2.751326	-1.720234
183	1	0	0.123464	1.263510	-2.663342
184	1	0	-1.418890	2.122874	-2.660735
185	1	0	-1.756503	1.671208	-0.279946
186	1	0	-1.609416	0.151040	-1.137815
187	1	0	0.764203	1.471569	0.255935
188	7	0	-0.517328	0.405855	1.500214
189	1	0	-0.967022	1.298019	1.778397
190	1	0	-1.231299	-0.337320	1.448607
191	6	0	0.498324	0.032556	2.543651
192	6	0	0.053225	0.351156	3.950336
193	6	0	1.032162	0.700142	4.890659
194	6	0	0.676761	0.984812	6.208835
195	6	0	-0.662726	0.933695	6.602615
196	6	0	-1.641827	0.594545	5.668994
197	6	0	-1.287341	0.301352	4.350763
198	1	0	-2.071890	0.082849	3.636614
199	1	0	-2.686296	0.555155	5.963342

200	1	0	-0.939519	1.161071	7.627296
201	1	0	1.444576	1.256246	6.927005
202	1	0	2.067065	0.775235	4.574475
203	1	0	0.716476	-1.027372	2.403620
204	1	0	1.408917	0.581181	2.312085

2j⊂1

Center Number	Atomic Number	Atomic Type	Coordinates (Angstroms) X	Y	Z
1	8	0	3.740950	1.196210	2.664027
2	6	0	4.157956	1.076229	1.342027
3	6	0	4.249902	2.225234	0.545027
4	6	0	4.628909	2.082251	-0.791973
5	6	0	4.919967	0.835265	-1.356973
6	6	0	4.802019	-0.285740	-0.529973
7	6	0	4.428015	-0.194758	0.815027
8	6	0	4.364072	-1.421761	1.718027
9	6	0	3.934132	-2.698781	1.036027
10	6	0	4.862165	-3.410738	0.261027
11	6	0	4.523219	-4.570753	-0.432973
12	6	0	3.218242	-5.052814	-0.253973
13	6	0	2.269212	-4.408859	0.541027
14	6	0	2.629155	-3.191842	1.153027
15	8	0	1.703120	-2.435885	1.876027
16	6	0	1.235146	-3.004907	3.122027
17	1	0	0.258168	-3.472952	2.987027
18	1	0	1.955180	-3.738873	3.496027
19	1	0	1.155108	-2.190911	3.843027
20	6	0	0.923242	-5.068921	0.747027
21	6	0	-0.270788	-4.407977	0.084027
22	6	0	-0.223805	-4.046975	-1.267973

23	6	0	-1.350827	-3.582027	-1.950973
24	6	0	-2.540833	-3.444083	-1.216973
25	6	0	-2.629818	-3.785087	0.133027
26	6	0	-1.480795	-4.272034	0.774027
27	8	0	-1.545779	-4.618037	2.121027
28	6	0	-1.936715	-5.975055	2.361027
29	1	0	-2.936706	-6.177101	1.959027
30	1	0	-1.225683	-6.679022	1.912027
31	1	0	-1.947709	-6.110055	3.444027
32	6	0	-3.943821	-3.718148	0.894027
33	6	0	-4.771878	-2.485187	0.621027
34	6	0	-5.811877	-2.519236	-0.315973
35	6	0	-6.593929	-1.395272	-0.603973
36	6	0	-6.305984	-0.221259	0.103027
37	6	0	-5.256988	-0.133210	1.022027
38	6	0	-4.492934	-1.280174	1.275027
39	8	0	-3.409938	-1.206123	2.148027
40	6	0	-3.730926	-1.467138	3.518027
41	1	0	-4.498958	-0.776174	3.888027
42	1	0	-2.810932	-1.323096	4.090027
43	1	0	-4.086878	-2.494155	3.659027
44	6	0	-4.893050	1.195807	1.640027
45	6	0	-3.936088	2.012852	0.779027
46	6	0	-4.031088	2.023848	-0.611973
47	6	0	-3.187125	2.798887	-1.417973
48	6	0	-2.213161	3.569932	-0.777973
49	6	0	-2.078162	3.594939	0.615027
50	6	0	-2.949125	2.808898	1.382027
51	8	0	-2.794123	2.769905	2.765027
52	6	0	-3.562169	3.744869	3.479027
53	1	0	-3.291216	4.762882	3.175027
54	1	0	-4.637162	3.597819	3.319027
55	1	0	-3.331162	3.609880	4.538027
56	6	0	-1.058203	4.483986	1.314027

57	6	0	0.276790	4.639048	0.620027
58	6	0	0.408750	5.492055	-0.485973
59	6	0	1.623741	5.675111	-1.149973
60	6	0	2.751770	5.049164	-0.598973
61	6	0	2.676809	4.215160	0.517027
62	6	0	1.414821	3.969102	1.081027
63	8	0	1.296864	3.035096	2.108027
64	6	0	1.510842	3.518106	3.451027
65	1	0	0.576845	3.443062	4.015027
66	1	0	1.848793	4.557122	3.432027
67	1	0	2.263871	2.886141	3.921027
68	6	0	3.935838	3.602219	1.099027
69	1	0	3.852841	3.545215	2.184027
70	1	0	4.774807	4.269258	0.876027
71	1	0	3.727762	5.222209	-1.040973
72	6	0	1.760702	6.509118	-2.432973
73	6	0	0.415675	7.103055	-2.889973
74	1	0	-0.002357	7.787035	-2.143973
75	1	0	-0.323289	6.322020	-3.095973
76	1	0	0.558648	7.672062	-3.812973
77	6	0	2.286745	5.587142	-3.559973
78	1	0	2.385719	6.148147	-4.495973
79	1	0	1.599784	4.752110	-3.731973
80	1	0	3.268765	5.169188	-3.317973
81	6	0	2.755648	7.669164	-2.204973
82	1	0	3.748665	7.307210	-1.924973
83	1	0	2.864621	8.261169	-3.119973
84	1	0	2.403617	8.334148	-1.409973
85	1	0	-0.474275	6.023013	-0.818973
86	1	0	-1.497250	5.483966	1.427027
87	1	0	-0.910185	4.094993	2.321027
88	1	0	-1.530188	4.163964	-1.366973
89	6	0	-3.379125	2.796878	-2.942973
90	6	0	-3.255057	1.354884	-3.481973

91	1	0	-2.259038	0.950930	-3.283973
92	1	0	-3.985026	0.681850	-3.023973
93	1	0	-3.418056	1.334876	-4.563973
94	6	0	-2.339165	3.673926	-3.664973
95	1	0	-1.316149	3.331974	-3.473973
96	1	0	-2.412214	4.723923	-3.360973
97	1	0	-2.507164	3.632919	-4.744973
98	6	0	-4.790150	3.340812	-3.270973
99	1	0	-4.949151	3.357805	-4.353973
100	1	0	-5.574121	2.718776	-2.827973
101	1	0	-4.914197	4.359806	-2.890973
102	1	0	-4.783059	1.399812	-1.078973
103	1	0	-5.809077	1.779765	1.796027
104	1	0	-4.436044	1.059829	2.620027
105	1	0	-6.895026	0.673714	-0.078973
106	6	0	-7.702929	-1.393324	-1.667973
107	6	0	-7.340977	-0.360307	-2.761973
108	1	0	-6.381966	-0.608262	-3.229973
109	1	0	-8.106978	-0.345343	-3.543973
110	1	0	-7.264025	0.653697	-2.354973
111	6	0	-9.049947	-1.004387	-1.016973
112	1	0	-9.009994	-0.012385	-0.557973
113	1	0	-9.328914	-1.723400	-0.239973
114	1	0	-9.845948	-0.990424	-1.769973
115	6	0	-7.869865	-2.767332	-2.341973
116	1	0	-8.664867	-2.717369	-3.091973
117	1	0	-6.953850	-3.082289	-2.852973
118	1	0	-8.145829	-3.542344	-1.619973
119	1	0	-6.001833	-3.459244	-0.820973
120	1	0	-4.540779	-4.602176	0.637027
121	1	0	-3.719817	-3.792138	1.958027
122	1	0	-3.435851	-3.072125	-1.704973
123	6	0	-1.347841	-3.290027	-3.461973
124	6	0	-2.346796	-4.251074	-4.151973

125	1	0	-2.070747	-5.295061	-3.969973
126	1	0	-2.352804	-4.082074	-5.233973
127	1	0	-3.366803	-4.105121	-3.784973
128	6	0	0.039169	-3.500963	-4.098973
129	1	0	0.796139	-2.845927	-3.654973
130	1	0	-0.008841	-3.273965	-5.167973
131	1	0	0.379217	-4.535947	-3.995973
132	6	0	-1.784909	-1.834048	-3.727973
133	1	0	-1.057942	-1.127014	-3.317973
134	1	0	-1.859917	-1.647051	-4.803973
135	1	0	-2.757919	-1.609093	-3.282973
136	1	0	0.722200	-4.166931	-1.782973
137	1	0	0.995290	-6.090918	0.357027
138	1	0	0.722247	-5.176931	1.813027
139	1	0	2.921285	-5.976828	-0.740973
140	6	0	5.498253	-5.307708	-1.364973
141	6	0	6.883222	-4.637643	-1.405973
142	1	0	7.539248	-5.190613	-2.084973
143	1	0	7.361222	-4.627621	-0.420973
144	1	0	6.825174	-3.607646	-1.773973
145	6	0	5.677322	-6.767700	-0.888973
146	1	0	4.731347	-7.315744	-0.886973
147	1	0	6.367346	-7.299667	-1.552973
148	1	0	6.087323	-6.799681	0.125027
149	6	0	4.912253	-5.295735	-2.796973
150	1	0	5.586277	-5.814704	-3.486973
151	1	0	4.781205	-4.269741	-3.154973
152	1	0	3.939276	-5.793781	-2.841973
153	1	0	5.872147	-3.022691	0.211027
154	1	0	5.363080	-1.583714	2.145027
155	1	0	3.703062	-1.207792	2.557027
156	1	0	4.987065	-1.267732	-0.937973
157	6	0	5.382971	0.738287	-2.820973
158	6	0	5.618039	-0.718702	-3.263973

159	1	0	5.933040	-0.738688	-4.311973
160	1	0	6.404062	-1.200666	-2.674973
161	1	0	4.708068	-1.321745	-3.179973
162	6	0	4.324942	1.361237	-3.759973
163	1	0	3.391969	0.789194	-3.731973
164	1	0	4.093894	2.396227	-3.492973
165	1	0	4.687943	1.358254	-4.792973
166	6	0	6.713935	1.513349	-2.970973
167	1	0	7.078938	1.449366	-4.000973
168	1	0	7.482955	1.099385	-2.309973
169	1	0	6.594886	2.572343	-2.722973
170	1	0	4.693867	2.979254	-1.399973
171	6	0	4.807946	1.293260	3.615027
172	1	0	5.445905	2.160290	3.403027
173	1	0	5.425988	0.389289	3.618027
174	1	0	4.339940	1.417238	4.594027
175	6	0	-0.006023	0.612035	0.142027
176	6	0	-1.103976	-0.387016	-0.204973
177	1	0	-1.426984	-0.214031	-1.231973
178	1	0	-1.980981	-0.271057	0.430027
179	1	0	-0.753928	-1.419000	-0.131973
180	1	0	-0.439069	1.609015	0.253027
181	6	0	1.153974	0.683089	-0.873973
182	6	0	0.779929	1.644072	-2.012973
183	1	0	-0.096054	1.288031	-2.565973
184	1	0	0.558883	2.644062	-1.629973
185	1	0	1.607925	1.731111	-2.719973
186	6	0	1.566038	-0.693891	-1.409973
187	1	0	1.820071	-1.396880	-0.612973
188	1	0	2.448034	-0.596850	-2.044973
189	1	0	0.767059	-1.145929	-2.001973
190	1	0	2.015954	1.123130	-0.361973
191	7	0	0.557991	0.320062	1.533027
192	1	0	1.171954	1.117090	1.778027

193	6	0	-0.514004	0.215012	2.585027
194	6	0	-0.034018	0.506034	3.985027
195	6	0	1.271994	0.252095	4.419027
196	6	0	1.639982	0.515112	5.740027
197	6	0	0.709958	1.034069	6.641027
198	6	0	-0.593055	1.296008	6.212027
199	6	0	-0.962042	1.036991	4.892027
200	1	0	-1.964053	1.267944	4.546027
201	1	0	-1.321074	1.708974	6.903027
202	1	0	0.997948	1.237082	7.668027
203	1	0	2.655991	0.310159	6.065027
204	1	0	2.024011	-0.106870	3.726027
205	1	0	-0.946958	-0.784009	2.496027
206	1	0	-1.294037	0.925975	2.317027
207	1	0	1.113032	-0.551913	1.521027

2k⊂1

Center Number	Atomic Number	Atomic Type	Coordinates (Angstroms) X	Y	Z
1	8	0	2.293041	2.595901	2.101021
2	6	0	3.134052	3.002877	1.063021
3	6	0	2.709082	4.029889	0.206021
4	6	0	3.461089	4.303868	-0.935979
5	6	0	4.632069	3.596835	-1.247979
6	6	0	5.069043	2.652822	-0.317979
7	6	0	4.348034	2.342843	0.844021
8	6	0	4.912005	1.341827	1.844021
9	6	0	5.025965	-0.082176	1.333021
10	6	0	6.078955	-0.455206	0.488021
11	6	0	6.207918	-1.749210	-0.022979
12	6	0	5.239891	-2.686182	0.363021

13	6	0	4.168901	-2.362152	1.199021
14	6	0	4.079938	-1.050150	1.689021
15	8	0	3.021948	-0.698120	2.521021
16	6	0	3.309943	-0.864128	3.917021
17	1	0	4.220958	-0.322154	4.198021
18	1	0	2.456954	-0.465104	4.465021
19	1	0	3.442913	-1.923132	4.170021
20	6	0	3.112871	-3.392122	1.525021
21	6	0	2.046866	-3.590092	0.449021
22	6	0	2.312872	-3.385100	-0.910979
23	6	0	1.360865	-3.633073	-1.905979
24	6	0	0.105851	-4.096037	-1.492979
25	6	0	-0.211155	-4.306028	-0.148979
26	6	0	0.771853	-4.045056	0.818021
27	8	0	0.466848	-4.214047	2.164021
28	6	0	0.814812	-5.496057	2.698021
29	1	0	0.501812	-5.492048	3.744021
30	1	0	1.894807	-5.676088	2.646021
31	1	0	0.295789	-6.301042	2.165021
32	6	0	-1.571170	-4.858990	0.242021
33	6	0	-2.748145	-3.959956	-0.088979
34	6	0	-3.328146	-3.999940	-1.362979
35	6	0	-4.417123	-3.194909	-1.711979
36	6	0	-4.959100	-2.385894	-0.705979
37	6	0	-4.423098	-2.319909	0.580021
38	6	0	-3.283120	-3.085941	0.868021
39	8	0	-2.630116	-2.930960	2.085021
40	6	0	-3.082140	-3.775947	3.146021
41	1	0	-4.143135	-3.607917	3.369021
42	1	0	-2.481132	-3.516964	4.020021
43	1	0	-2.941170	-4.835951	2.906021
44	6	0	-5.071073	-1.437890	1.639021
45	6	0	-5.177032	0.022113	1.245021
46	6	0	-6.244019	0.476143	0.467021

47	6	0	-6.348982	1.801146	0.021021
48	6	0	-5.357956	2.693118	0.437021
49	6	0	-4.277968	2.288087	1.234021
50	6	0	-4.190006	0.949085	1.617021
51	8	0	-3.092018	0.522053	2.363021
52	6	0	-3.348019	0.478061	3.781021
53	1	0	-3.201991	1.467057	4.231021
54	1	0	-2.644039	-0.227959	4.218021
55	1	0	-4.373028	0.150090	3.979021
56	6	0	-3.209940	3.275057	1.635021
57	6	0	-2.105934	3.485026	0.606021
58	6	0	-2.332939	3.319032	-0.765979
59	6	0	-1.349931	3.582004	-1.726979
60	6	0	-0.112918	4.050969	-1.266979
61	6	0	0.158087	4.239961	0.091021
62	6	0	-0.838922	3.921990	1.025021
63	8	0	-0.551919	4.004982	2.384021
64	6	0	-0.934884	5.231992	3.018021
65	1	0	-0.456860	6.091979	2.533021
66	1	0	-2.020880	5.374023	3.002021
67	1	0	-0.596886	5.163983	4.053021
68	6	0	1.476105	4.848924	0.525021
69	1	0	1.440110	5.047925	1.595021
70	1	0	1.577132	5.819921	0.029021
71	1	0	0.677089	4.281947	-1.975979
72	6	0	-1.586937	3.378011	-3.232979
73	6	0	-0.604967	2.304983	-3.759979
74	1	0	-0.783995	1.340988	-3.272979
75	1	0	-0.734971	2.165987	-4.837979
76	1	0	0.438041	2.582954	-3.580979
77	6	0	-1.336899	4.710004	-3.976979
78	1	0	-1.507903	4.582009	-5.050979
79	1	0	-0.310889	5.064975	-3.845979
80	1	0	-2.011877	5.491023	-3.614979

81	6	0	-3.022950	2.915052	-3.543979
82	1	0	-3.255977	1.950058	-3.081979
83	1	0	-3.766929	3.645073	-3.205979
84	1	0	-3.144953	2.797055	-4.624979
85	1	0	-3.308948	2.971060	-1.073979
86	1	0	-3.688912	4.246070	1.819021
87	1	0	-2.747948	2.976044	2.577021
88	1	0	-5.401927	3.736119	0.144021
89	6	0	-7.505970	2.204179	-0.906979
90	6	0	-7.420994	1.359176	-2.200979
91	1	0	-8.224986	1.635199	-2.890979
92	1	0	-6.463990	1.520149	-2.708979
93	1	0	-7.515024	0.288179	-1.994979
94	6	0	-8.856978	1.940217	-0.204979
95	1	0	-9.686970	2.215240	-0.862979
96	1	0	-8.980007	0.886220	0.061021
97	1	0	-8.939961	2.530219	0.714021
98	6	0	-7.445928	3.691177	-1.301979
99	1	0	-6.520921	3.932151	-1.835979
100	1	0	-7.521909	4.347179	-0.427979
101	1	0	-8.280921	3.930200	-1.965979
102	1	0	-7.016039	-0.237835	0.199021
103	1	0	-6.080084	-1.818862	1.835021
104	1	0	-4.505076	-1.531906	2.562021
105	1	0	-5.816083	-1.762869	-0.934979
106	6	0	-5.011122	-3.146892	-3.128979
107	6	0	-4.869081	-1.705896	-3.677979
108	1	0	-5.293079	-1.638884	-4.684979
109	1	0	-3.815073	-1.412926	-3.732979
110	1	0	-5.386060	-0.976882	-3.047979
111	6	0	-4.292149	-4.103913	-4.095979
112	1	0	-3.229142	-3.857943	-4.193979
113	1	0	-4.376178	-5.146910	-3.773979
114	1	0	-4.739147	-4.028900	-5.091979

115	6	0	-6.507133	-3.532850	-3.082979
116	1	0	-6.636161	-4.545846	-2.689979
117	1	0	-6.940132	-3.497838	-4.087979
118	1	0	-7.084113	-2.851833	-2.449979
119	1	0	-2.910165	-4.688952	-2.086979
120	1	0	-1.715197	-5.811985	-0.279979
121	1	0	-1.564176	-5.074990	1.308021
122	1	0	-0.656155	-4.307015	-2.233979
123	6	0	1.664869	-3.471081	-3.405979
124	6	0	1.599830	-4.865079	-4.073979
125	1	0	1.827832	-4.788086	-5.141979
126	1	0	0.605817	-5.313051	-3.974979
127	1	0	2.322810	-5.549100	-3.617979
128	6	0	3.061886	-2.875121	-3.656979
129	1	0	3.856868	-3.525143	-3.278979
130	1	0	3.174914	-1.892124	-3.188979
131	1	0	3.222889	-2.753125	-4.732979
132	6	0	0.622895	-2.543052	-4.070979
133	1	0	-0.403114	-2.891023	-3.915979
134	1	0	0.694924	-1.526054	-3.673979
135	1	0	0.796897	-2.493057	-5.150979
136	1	0	3.291882	-3.018127	-1.184979
137	1	0	3.610844	-4.356136	1.693021
138	1	0	2.604878	-3.140108	2.456021
139	1	0	5.307862	-3.708184	0.000021
140	6	0	7.340906	-2.161242	-0.975979
141	6	0	8.273940	-0.985268	-1.319979
142	1	0	8.771951	-0.587283	-0.429979
143	1	0	7.732963	-0.167253	-1.807979
144	1	0	9.053930	-1.322291	-2.008979
145	6	0	6.724891	-2.687225	-2.293979
146	1	0	6.098866	-3.568207	-2.127979
147	1	0	7.514883	-2.971247	-2.997979
148	1	0	6.104913	-1.920207	-2.768979

149	6	0	8.184875	-3.279266	-0.320979
150	1	0	8.990866	-3.592289	-0.993979
151	1	0	8.636884	-2.929279	0.613021
152	1	0	7.580850	-4.162249	-0.091979
153	1	0	6.822976	0.293773	0.246021
154	1	0	5.911015	1.687799	2.134021
155	1	0	4.295006	1.350844	2.739021
156	1	0	5.994027	2.120796	-0.491979
157	6	0	5.372077	3.874814	-2.564979
158	6	0	5.834119	5.348801	-2.603979
159	1	0	6.519125	5.564781	-1.777979
160	1	0	4.990139	6.041825	-2.528979
161	1	0	6.355125	5.559786	-3.542979
162	6	0	6.608052	2.971779	-2.740979
163	1	0	7.089058	3.187765	-3.698979
164	1	0	7.351056	3.139758	-1.954979
165	1	0	6.337021	1.909786	-2.738979
166	6	0	4.407069	3.602841	-3.743979
167	1	0	4.061040	2.563851	-3.732979
168	1	0	4.911075	3.785827	-4.697979
169	1	0	3.525088	4.250866	-3.704979
170	1	0	3.120112	5.097878	-1.594979
171	6	0	2.508059	3.226895	3.372021
172	1	0	3.576060	3.281865	3.605021
173	1	0	2.082087	4.234907	3.390021
174	1	0	2.005041	2.608909	4.116021
175	6	0	0.290982	0.498958	-0.306979
176	6	0	-0.592048	-0.536017	-0.999979
177	6	0	-2.094040	-0.277975	-0.928979
178	1	0	-2.352013	0.706033	-1.324979
179	1	0	-2.471042	-0.335964	0.093021
180	1	0	-2.635062	-1.033959	-1.500979
181	1	0	-0.270048	-0.549026	-2.044979
182	1	0	-0.358076	-1.534024	-0.616979

183	6	0	1.786973	0.189915	-0.442979
184	6	0	2.372987	0.694899	-1.762979
185	1	0	3.432980	0.430869	-1.821979
186	1	0	1.867975	0.259913	-2.630979
187	1	0	2.301018	1.783901	-1.831979
188	1	0	2.334986	0.649900	0.380021
189	1	0	1.945942	-0.887089	-0.355979
190	1	0	0.079010	1.497964	-0.695979
191	7	0	-0.061015	0.625968	1.180021
192	1	0	0.625004	1.282948	1.585021
193	1	0	-0.989003	1.057994	1.285021
194	6	0	-0.057051	-0.656032	1.954021
195	6	0	-0.099045	-0.440031	3.445021
196	6	0	-0.132078	-1.593030	4.243021
197	6	0	-0.159075	-1.487030	5.632021
198	6	0	-0.151039	-0.228030	6.243021
199	6	0	-0.124007	0.918969	5.451021
200	6	0	-0.102009	0.816969	4.056021
201	1	0	-0.119983	1.734969	3.476021
202	1	0	-0.130979	1.900970	5.914021
203	1	0	-0.169037	-0.146029	7.325021
204	1	0	-0.181100	-2.385029	6.241021
205	1	0	-0.124105	-2.566031	3.758021
206	1	0	0.852934	-1.192058	1.687021
207	1	0	-0.923068	-1.239008	1.640021
